# Using the Intervention Mapping Approach to Develop a Mental Health Intervention: A Case Study on Improving the Reporting Standards for Developing Psychological Interventions

**DOI:** 10.3389/fpsyg.2021.648678

**Published:** 2021-10-05

**Authors:** Joep van Agteren, Matthew Iasiello, Kathina Ali, Daniel B. Fassnacht, Gareth Furber, Lydia Woodyatt, Alexis Howard, Michael Kyrios

**Affiliations:** ^1^Wellbeing and Resilience Centre, Lifelong Health Theme, South Australian Health and Medical Research Institute, Adelaide, SA, Australia; ^2^Órama Institute for Mental Health and Wellbeing, Adelaide, SA, Australia; ^3^College of Nursing and Health Science, Flinders University, Adelaide, SA, Australia; ^4^College of Education, Psychology and Social Work, Flinders University, Adelaide, SA, Australia; ^5^Health Counselling and Disability Services, Flinders University, Adelaide, SA, Australia

**Keywords:** wellbeing, intervention research, intervention development, mental health promotion and prevention, mental health – state of emotional and social well-being

## Abstract

Replicating or distilling information from psychological interventions reported in the scientific literature is hindered by inadequate reporting, despite the existence of various methodologies to guide study reporting and intervention development. This article provides an in-depth explanation of the scientific development process for a mental health intervention, and by doing so illustrates how intervention development methodologies can be used to improve development reporting standards of interventions. Intervention development was guided by the Intervention Mapping approach and the Theoretical Domains Framework. It relied on an extensive literature review, input from a multi-disciplinary group of stakeholders and the learnings from projects on similar psychological interventions. The developed programme, called the “Be Well Plan”, focuses on self-exploration to determine key motivators, resources and challenges to improve mental health outcomes. The programme contains an online assessment to build awareness about one’s mental health status. In combination with the exploration of different evidence-based mental health activities from various therapeutic backgrounds, the programme teaches individuals to create a personalised mental health and wellbeing plan. The use of best-practice intervention development frameworks and evidence-based behavioural change techniques aims to ensure optimal intervention impact, while reporting on the development process provides researchers and other stakeholders with an ability to scientifically interrogate and replicate similar psychological interventions.

## Introduction

Psychological interventions, being activities or groups of activities aimed to change behaviours, feelings and emotional states (Hodges et al., 2011), come in many shapes and sizes. A popular delivery method is in the form of programmes consisting of several interacting components and procedures, which per definition makes them “complex interventions” (Moore et al., 2015). This complexity is often lost in academic publications, as articles for instance are bound to word limits or have a primary focus on presenting outcome data as opposed to theoretical rationale and methodological insights (O’Cathain et al., 2019). Despite various welcome initiatives such the Template for Intervention Description and Replication (TIDieR) the intervention literature typically lacks in-depth descriptions of psychological interventions and the way they were created (Pino et al., 2012; Candy et al., 2018).

These reporting challenges are problematic for the scientific method as they make it difficult to replicate interventions, interpret which underlying intervention components are effective and draw robust conclusions about how these interventions have been developed (Chalmers and Glasziou, 2009; Hoffmann et al., 2017). More importantly, these challenges are avoidable as robust intervention development methodologies already exist that can be used to scientifically describe the components of complex behavioural and psychological interventions (Michie et al., 2011b; Eldredge et al., 2016; Garba and Gadanya, 2017). Scientific articles that purely describe the development of interventions using such methodologies can mainly be found in research on health behaviours, including smoking (van Agteren et al., 2018b), nutrition (Rios et al., 2019), physical activity (Boekhout et al., 2017), AIDS (Wolfers et al., 2007), and oral hygiene (Scheerman et al., 2018) to name a few. Despite their potential merit, the application of similar methodologies has yet to receive traction in psychological science.

Rigour in reporting standards is particularly important for new and emerging scientific areas in gaining scientific credibility and facilitating replication. The last decades have seen the introduction of a range of new psychological interventions, as well as the re-purposing of existing interventions, specifically aimed at promoting mental wellbeing, as opposed to addressing mental disorder *per se* (Slade, 2010). Improving outcomes of mental wellbeing is a protective factor against the onset of mental illness (Keyes et al., 2010; Iasiello et al., 2019), aids in disease recovery and chronic disease self-management and is associated with improved health service utilisation (Lamers et al., 2012; Slade et al., 2017). Above all, feeling mentally well is an important outcome in its own right, for individuals, families, communities, and society (Diener and Seligman, 2018; Diener et al., 2018). As a result, psychological interventions are increasingly in demand by health organisations, educational providers, workforces, and governments looking at wellbeing initiatives. Considering this interest, the individual and societal benefits of improving wellbeing, and fair criticism that have been drawn toward the lack of rigour in wellbeing research (Heintzelman and Kushlev, 2020), it is important to adequately describe the development of any interventions aimed at improving outcomes of mental wellbeing (Diener, 2003; Gable and Haidt, 2005; Kristjánsson, 2012).

The aim of the current article is to be a case study that firstly describes the application of a rigorous intervention development framework, the Intervention Mapping (IM) approach (Eldredge et al., 2016), to guide development of a theory- and evidence-based mental health intervention, designed to be used with both clinical and non-clinical populations. Secondly, as a result, it aims to act as a case study on the complexity that underpins scientific mental health interventions, and the detail that needs to be considered when aiming to replicate or modify them.

## Materials and Methods

**FIGURE 1:**
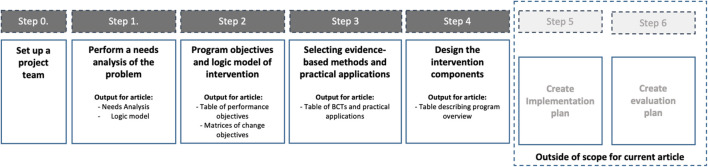
Visualisation of the Intervention Mapping process and the outputs at each of the steps covered in the intervention development methodology.

The IM approach guides intervention development in a series of six steps. This article follows the standard structure of the IM methodology. The methods below explain each of the four steps and how they were used for the development of the intervention in this article: the Be Well Plan. For a description of the methodology see Kok et al. (2016). Figure 1 visualises the methodology steps and its outputs at each stage, which are presented in the results below. The large bulk of programme development across the four steps was conducted by a small project team (JA, MI, KA, DF, and GF) who interacted with a larger multi-disciplinary project working group that among others included psychologists, counsellors, mental health researchers, and end-users, throughout the development life cycle. This group was crucial in informing and validating each of the IM steps, such as the exact objectives of the programme. The role for each of the members is described in more detail in Supplementary Appendix 1.

### Step 1: Determine the Problem That Needs to Be Solved by the Intervention *via* a Thorough Needs Analysis

The first step of IM involved performing a needs analysis related to the problem that the programme aims to solve. The needs analysis focuses on determining the problem that needs to be changed and subsequently defining the exact scope of the intervention. The needs analysis firstly draws on an extensive study of the scientific literature on mental health and wellbeing interventions. Secondly, it is underpinned by findings and data (published and unpublished) from previous wellbeing studies our research group conducted across population groups including in the general community, within workforces such as health professionals, with older adults, carers, and disadvantaged youths (Raymond et al., 2018, 2019; van Agteren et al., 2018a; Bartholomaeus et al., 2019). All data that was being used to underpin the needs analysis was subject to ethics approvals issued by the Flinders University Social and Behavioural Research Ethics Committee (SBREC), project numbers (PN) 7834, 7891, 7350, 7358, 7221, 7218, and 8579.

The IM framework uses the PRECEDE-PROCEED model (Gielen et al., 2008) to summarise and structure the results of the needs analysis into an actionable logic model. In highly simplified terms, the model gets you to (1) determine the key problem that the needs analysis indicates one needs to solve, (2) the overarching behavioural and environmental outcomes (or targets) one needs to meet to improve the problem, and (3) defining the underlying determinants of those behavioural and environmental outcomes. An example could be, “people with problematic mental health (the problem), may not consistently use psychological activities in their day-to-day lives (the outcome/target) as they do not have enough knowledge (the determinant) of the benefits of using such activities.”

Rather than arbitrarily coming up with determinants that we wished to change, we relied on the Theoretical Domains Framework (Cane et al., 2012) to guide our choice. The TDF is a framework that synthesises 14 unique determinants (e.g., knowledge, skills, and beliefs) stemming from 33 behaviour change and implementation theories. It provides a comprehensive and intuitive theory-based overview of relevant behaviour change determinants that intervention developers can use. IM requires developers to prioritise and choose only the relevant TDF determinants by assigning which determinants (1) are actually related to the problem and (2) can actually be changed. An explanation for the choice of each determinant is provided in Supplementary Appendix 1.

The result of step 1 is a logic model of change that summarises the problem, the outcomes and the determinants, which will be used to underpin the intervention.

### Step 2: Define the Objectives the Intervention Needs to Meet and What the Intervention Needs to Change to Meet Those Objectives

After determining the problem that needs to be addressed, IM continuous to delineate what needs to change to solve the problem. Firstly, each target area (e.g., the lack of participation in psychological activities) identified in the needs analysis were rewritten into desired behavioural and environmental outcomes (e.g., engaging in regular use of psychological activities). Secondly, these outcomes were subsequently broken into sub-objectives called performance objectives (e.g., demonstrates knowledge on how to improve mental health). Finally, these performance objectives were broken down further into so-called change objectives. These are very specific objectives that need to be achieved in order for the performance objective to be realised (e.g., *increasing* knowledge of malleability of mental health). A change objective consisted of linking performance objective with determinants from the Theoretical Domains Framework (e.g., knowledge, skill, and beliefs about capabilities). The final output of step 2 was a collection of matrices, so-called matrices of change, depicting each change objective per performance objective (placed in the rows) and determinant (placed in the columns).

### Step 3: Select Behaviour Change Techniques and Practical Applications of Those Techniques That Will Be Used to Achieve the Change Objectives

In step 3, a new table is created by placing the change objectives on individual rows and matching them with evidence-based “behaviour change techniques” (BCTs) (Kok et al., 2016). BCTs are theoretical strategies (e.g., goal setting, modelling, and active learning) that have been empirically proven to be able to change individual behaviour. The IM framework comes with an extensive summary of BCTs and how they can be used to create impactful interventions. It gets programme developers to match their change objectives with individual BCTs, thereby aiming to improve the chance that actual behaviour and environmental change in line with the change objective will be achieved.

The final part of step 3 is translating the theoretical BCTs into so-called “practical applications,” referring to the proposed real-world application of each BCT. For example, to achieve the change objective “Demonstrating knowledge on malleability of mental health,” the programme draws on the BCT “active learning” which can be achieved *via* the “practical application” of showing an engaging video on epigenetic changes that can alter our mental health (Schiele et al., 2020). The result is a line-by-line itemised list (or blueprint) of practical applications that need to be incorporated into the programme design in step 4.

### Step 4: Design and Develop the Actual Intervention Components Based of the Practical Applications Identified in the Previous Step

In step 4, the programme designers created the actual intervention based on the blueprint established in step 3. This process was guided *via* various project team meetings. A subgroup of project team members (JA, MI, KA, DF, LW, and GF) created a programme delivery framework, outlining the proposed intervention sessions, their underpinning rationale and the delivery format. This framework was evaluated and approved by the larger multi-disciplinary project team that included end-users over a series of meetings. The subgroup continued by creating a detailed narrative for the programme, which was subsequently translated into an interactive programme. The narrative and programme content is presented in the results below.

After developing the first iteration of the programme, two small-scale in-person test runs with university students (*n* = 30) and colleagues of the project team members (*n* = 7) were conducted by JA, MI, KA, GF, and AH. Feedback from these test runs was used to iterate the programme delivery, not to determine impact on outcomes (i.e., they were test runs not evaluation studies). After iteration, each of the five session sessions were recorded on video and the programme was subsequently tested in an online delivery format, i.e., delivered *via* video conferencing software, resulting in the programme presented in this manuscript.

### Steps 5 and 6: Adoption, Implementation, and Evaluation Plans

After finishing the design and development of the intervention, IM concludes with two additional steps, the development of an adoption and implementation plan (step 5) as well as an evaluation plan (step 6). These two steps are outside the scope of this article, as the aim here is to describe the development and design process. The actual evaluation of the programme on outcomes will be covered in subsequent publications, including a pre-post pilot study in university students and general community members (*n* = 89; van Agteren et al., 2021a) and a randomised controlled study in university students. A brief description of the evaluation approach is provided in the discussion of this manuscript.

## Results

### Step 1: The Needs Analysis of the Be Well Plan Programme

The results from the needs analysis are presented in a narrative format, combining the findings from the literature review and interrogation of qualitative and quantitative data from previous projects on wellbeing interventions our project team conducted. The needs analysis for this specific programme is structured around four distinct themes, which are outlined below. Supporting material underpinning the needs analysis can be found in Supplementary Appendix 1.

#### Theme 1: There Is a Need for Mental Health Interventions to Incorporate a Specific Focus on Positive and Adaptive States, in Addition to Taking Psychological Distress Into Account

Psychological interventions for mental health are often thought of to be synonymous to interventions aimed at treating or preventing mental illness or psychological distress. This is reflected in research on psychological interventions such as Cognitive Behavioural Therapy (CBT), Acceptance and Commitment Therapy (ACT) and mindfulness, with studies largely focussing on their effectiveness in improving outcomes of illness and psychological distress (Hofmann et al., 2010, 2012; Swain et al., 2013). There is however general agreement that optimal mental health does not equate to a mere absence of symptoms of mental illness as it also requires participants to demonstrate high levels of mental wellbeing, e.g., finding meaning in life, working on positive relationships, and building positive emotions (Jahoda, 1958; Smith, 1959; Fontana et al., 1980; Wilkinson and Walford, 1998; Greenspoon and Saklofske, 2001; Keyes, 2003, 2005; Suldo and Shaffer, 2008). A significant body of research has found that mental wellbeing should not be seen as the mere opposite of mental illness (Iasiello et al., 2020). Studies in Western and non-Western populations demonstrate that people who exhibit psychological distress or show symptoms of mental illness have varying levels of mental wellbeing (Peter et al., 2011; Seow et al., 2016; Bariola et al., 2017; Teismann et al., 2017; Xiong et al., 2017).

There are numerous ways of building outcomes of mental wellbeing, including spending time in nature (Howell et al., 2011; Korpela et al., 2016; Passmore and Holder, 2017), being physically active (Penedo and Dahn, 2005; Windle, 2014), doing yoga (Ivtzan and Papantoniou, 2014; Sharma et al., 2017), and spending more time engaging in social relationships (Keyes, 1998; Gallagher and Vella-Brodrick, 2008) among others. Psychological interventions such as CBT, ACT and mindfulness in addition to be effective for outcomes of mental illness (Hofmann et al., 2012; Öst, 2014; Goldberg et al., 2018) have joined this list in being able to improve outcomes of mental wellbeing, in addition to being effective for distress. A recent systematic review conducted by authors of the current article examined 419 studies (*n* = 53,288 included in meta-analysis) which clearly demonstrated their impact in both healthy populations and populations with mental illness or physical illness (van Agteren et al., 2021b). The significant findings were dependent on the specific target population (e.g., clinical versus non-clinical populations) and other moderators, most notably intervention intensity.

Psychological interventions are not simply beneficial for improving mental health outcomes in the moment. For instance, by improving outcomes of wellbeing, they can both increase the likelihood of recovery from mental illness or can prevent the onset of illness in the future (Keyes et al., 2010; Wood and Joseph, 2010; Grant et al., 2013; Lamers et al., 2015; Iasiello et al., 2019). By focussing on improving wellbeing it makes them a viable avenue for individuals seeking to reduce symptoms of distress (Gilbert, 2012; Schotanus-Dijkstra et al., 2019) and to build resilience to future adversity (Fritz et al., 2018). In other words, by teaching psychological skills that take future distress *and* wellbeing into account, participants can be taught techniques that aim to help them withstand adversity or stress (i.e., cope with) without succumbing to more serious mental health problems (Davydov et al., 2010; Harms et al., 2018). A deliberate focus on developing this resilience, or in other words improving adaptative states, could strengthen the impact of mental health interventions, for those with and without current distress (Roy et al., 2007; Fritz et al., 2018).

#### Theme 2: There Is a Need for Mental Health Interventions to Target Malleable Non-psychological Determinants of Mental Health in Psychological Interventions

Our mental health is not simply determined by our thinking patterns, but rather is influenced by a myriad of bio-psycho-social influences. While not all these influences are within the control of behavioural or psychological interventions, or feasible in light of the focus for our intervention, the team determined that two aspects were. Firstly, stimulating positive change related to our physical health will be beneficial to our mental health, as both are intrinsically linked, which is demonstrated by a considerable body of scientific evidence on the importance of health promotive factors such as physical activity, nutrition and sleep, all of which can be positively addressed using behavioural interventions (Valois et al., 2004; Penedo and Dahn, 2005; Chu and Richdale, 2009; Deslandes et al., 2009; Oddy et al., 2009; Rethorst et al., 2009; Nanri et al., 2010; Gradisar et al., 2011; Rienks et al., 2013; Bernert et al., 2014; Dalton and Logomarsino, 2014; Jacka et al., 2015). Inclusion of, at minimum, rudimentary techniques that could be used to stimulate positive health behaviours was deemed necessary for our intervention. Secondly, the training needed to incorporate elements of our social environment into the intervention. Stimulating small positive change in our social environment can lead to improved mental health (Kawachi and Berkman, 2001; Santini et al., 2015; Verduyn et al., 2017). Similarly, feeling isolated and lonely exerts strong negative influence on wellbeing and mental health (Arslan, 2018; Wang et al., 2018).

#### Theme 3: Personalising the Mental Health Intervention to Match Individual Participant Needs Will Drive Impact and Is Feasible in Scalable Intervention Formats

In-person psychological mental health interventions outside the clinical setting tend to come in predictable formats. They often are delivered in groups (as this cost-effective), are delivered over multiple sessions, with content tending to come from (a combination of compatible) therapeutic paradigms. The content typically tends to be similar for all participants, despite the fact that personalising or tailoring interventions to individual needs might improve outcomes of interventions or improve the feasibility of its implementation (Norcross and Wampold, 2011). To improve tailoring, intervention developers often adjust the content of interventions to fit specific target *populations* such as students, older adults, or workforces (Waters, 2011; Shiralkar et al., 2013; Proyer et al., 2014; Robertson et al., 2015). While tailoring to group-needs is a right step in the direction, it is still removed from addressing the needs and preferences of *individuals* within each population group (Schork, 2015).

One potential way to achieve tailoring to individual needs is to allow participants to work on specific resources and barriers that are relevant to their unique lives. Rather than utilising an approach based on a singular therapeutic model (e.g., CBT versus ACT) the intervention could focus on modelling the approach by recent innovations such as process-based interventions; the intervention could incorporate a range of effective intervention techniques that target known “theoretically derived and empirically supported processes that are responsible for positive treatment change” rather than focussing on a specific illness, medical diagnosis or set therapeutic paradigm (Hayes and Hofmann, 2018; Hofmann and Hayes, 2019). These techniques can come from varying evidence-based interventions, for instance those identified in our systematic review on psychological interventions to improve mental wellbeing (van Agteren et al., 2021b).

By facilitating tailoring to individual circumstances engagement with the intervention can be stimulated, as participants in mental health training offerings may resonate differently to different components of an intervention. This is reflected in responses to training feedback in previous projects the team conducted, see Supplementary Appendix 1. The training delivered in these projects consisted of skills stemming from CBT, mindfulness techniques and positive psychology. At the end of the training participants voices different preferences for different skills, with an eclectic response pattern noted. Allowing participants to experiment with different evidence-based techniques in an effective manner has furthermore become much more within reach with the rise of technology (Clough and Casey, 2015; Dinesen et al., 2016; Naslund et al., 2016; Naslund, 2017; Berrouiguet et al., 2018). For instance, technology can help guide activity recommendations based on an individual’s response to scientific questionnaires for mental health and wellbeing. This can allow a participant to experiment with different techniques, without the requirement for a trainer or therapist to guide choice of activities, ultimately facilitating them to independently form a personalised strategy for good mental health and wellbeing.

#### Theme 4: In Order to Facilitate Lasting Change, There Is a Need for Mental Health Interventions to Leverage a Focus on Behaviour Change

In order for mental health interventions to “stick,” individual participants need to change their behaviour, aligned to the goals of the intervention (Kok et al., 2016). Simply providing activities to build resources and remove challenges to good mental health may not be sufficient, for example, due to a discrepancy between intention to change behaviour and actual behaviour change (Atkins et al., 2017). Reliance on the IM approach stimulated an explicit focus on behaviour change, ultimately asking intervention developers to select key underpinning determinants that are related to the problem behaviour.

Interventions can broach this in numerous ways, depending on the determinants they consider to be the focus for the intervention. As part of the needs analysis, the project team focused on several determinants that were (1) deemed important for mental health and (2) were considered to be malleable and within reach of the current intervention. For instance, teaching *skills* to deal with stressors, adversity or negative social influence aids in improving the chance of engaging in wellbeing activities (Fritz et al., 2018). *Knowledge* has been found to be one of the essential ingredients for psychological skills to be developed (Jorm, 2012; Oades, 2017), and *self-efficacy* helps in the execution of skills (Leamy et al., 2011; Trompetter et al., 2017). Often there is resistance or stigma toward mental health and wellbeing activities (Clement et al., 2015; Thornicroft et al., 2016), indicating the need to focus on changing *beliefs about the effectiveness* of wellbeing behaviours and *beliefs about the consequences* of implementing those behaviours (Sheeran et al., 2016). Finally, *goal-setting* aids in strategy formation and achievement of physical health improvements as well as behavioural regulation *via* self-monitoring (Wollburg and Braukhaus, 2010; Michie et al., 2011a). A further justification for why the project team chose these determinants over others can be found in Supplementary Appendix 1.

#### Use the Needs Analysis to Craft a Visual Logic Model for the Intervention

**FIGURE 2:**
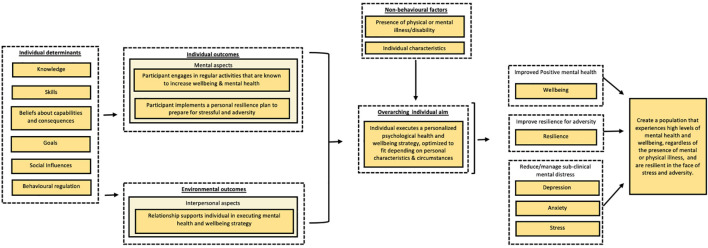
Simplified overview of the needs analysis for the Be Well Plan. The specific explanatory role of variables (e.g., mediating or moderating) is not taken into account in this figure. The model merely serves to outline the variability of behaviours and determinants that influence wellbeing and resilience using the PRECEDE-PROCEED model outline. Determinants are based on the Theoretical Domains Framework (TDF).

The project team subsequently set out to construct a logic model for the intervention based on the findings from the needs analysis, see Figure 2. The team followed the general structure for logic models as set out in IM and the PRECEDE-PROCEED model (Green and Kreuter, 1999). The key focus for the intervention was to help participants promote their mental health, pointing to the need for an intervention that would be able to target positive, adaptive, and distress states. The needs analysis pointed to the desire for an intervention that allowed participants to develop a personalised mental health and wellbeing strategy or “plan,” allowing participants to take their unique characteristics and health status into account. The key objective was to get participants to change their behaviour by actively engaging in evidence-based activities. These activities firstly should allow individuals to build or leverage resources that can promote mental health in the now and secondly build resources that can help the individual cope with stressors in the future. It should thirdly aim to engage the social environment as a mechanism to support the individual. To achieve the objective, and ultimately behaviour change, the intervention would target specific behaviour change determinants that were derived from the Theoretical Domains Framework Domains (Atkins et al., 2017), including knowledge, skills, beliefs about capabilities and consequences, goals, social influences, and behavioural regulation.

### Step 2: Definition of Programme Objectives

Step 2 required the project team to create programme outcomes based on the needs analysis and the logic model. The outcomes were: participant engages in regular activities that are known to increase wellbeing and mental health, participant implements a personal resilience plan to prepare for stressful periods and adversity, participant engages relationship supports in executing their mental health and wellbeing strategy. These outcomes were further specified into performance objectives, see Table 1. Change objectives were formulated for each of the performance objectives in line with the chosen TDF determinants mentioned earlier. All matrices of change can be found in Supplementary Appendix 1.

**TABLE 1:** Behavioural and environmental outcomes, and performance objectives (PO) for Complete Mental Health.

**Behavioural outcome 1: Engages in regular activities that are known to increase the mental health and wellbeing of the individual**	
PO 1.1	Develops understanding of mental health, and its relationship to mental illness and mental wellbeing
PO 1.2	Understands that good psychological health can actively be achieved *via* different intervention types, regardless of physical or mental illness
PO 1.3	Understands that personal characteristics influence which interventions should be selected to improve mental health
PO 1.4	Understands that good psychological health requires a life-course approach
PO 1.5	Is aware of personal psychological health profile (wellbeing, resilience, and psychological distress)
PO 1.6	Creates overview of resources and challenges for their psychological health
PO 1.6.1	Determines which resources and challenges are currently present, which can be improved on using the programme and which ones are out of scope
PO 1.6.2	Determines when other programmes or services for mental health and mental wellbeing need to be considered
PO 1.7	Determines personal motivators for wanting to engage in activities that promote psychological health
PO 1.8	Develops a personal psychological health strategy
PO 1.8.1	Identifies barriers and enablers to implementing a personal psychological health strategy
PO 1.9	Maintains use of personal psychological health strategy over time
PO 1.10	Evaluates implementation of psychological health strategy
PO 1.10.1	Judges whether psychological health strategy is being executed successfully
PO 1.10.2	Re-evaluates psychological health strategy, if not effective achieving personal outcomes
PO 1.10.3	Contacts professional care when mental health and wellbeing symptoms impact personal life
PO 1.10.4	Adjusts psychological health strategy and returns to PO 1.8
**Behavioural outcome 2: Implements a personal resilience plan to prepare for stressors and adversity**	
PO 2.1	Understands the concept of resilience and its relationship to psychological health
PO 2.2	Understands the impact of different stressor types or adversities on psychological health (chronic versus acute, foreseen versus unforeseen)
PO 2.3	Understands how effective use of psychological health strategies can lead to post adversity growth and better psychological health
PO 2.4	Understand that their personal characteristics influence which interventions they should be considering to build resilience
PO 2.5	Is aware of personal resilience status
PO 2.6	Identifies potential resources and challenges for their resilience
PO 2.6.1	Determines which resources and challenges are currently present, which can be improved on using the programme and which ones are out of scope
PO 2.7.	Identifies personal motivators for regularly engaging in resilience strategy
PO 2.8	Develops a personal resilience plan
PO 2.9	Practices resilience strategies on a regular basis regardless of presence of adversity
PO 2.10	Uses resilience strategies when facing personal adversity
PO 2.11	Evaluates implementation of resilience plan
PO 2.11.1	Judges whether resilience strategy is being executed successfully
PO 2.11.2	Re-evaluates resilience strategies if they are not effective
PO 2.11.3	Contacts professional care when mental health symptoms impact personal life
PO 2.11.4	Adjusts resilience strategy and returns to PO 2.8
PO 2.12	Identifies personal strength and growth in dealing with adversity
**Environmental outcome 1: Identified relationship support helps individual in executing their mental health and wellbeing strategy**	
PO 3.1	Identified relationship support develops an understanding of the personal psychological health strategy of the training participant
PO 3.2	Identified relationship support participates in individual’s psychological health activities when requested
PO 3.4	Identified relationship support checks up if individual is practicing use of strategies over time
PO 3.5	Identified relationship support reminds individual of thinking about mental health strategies when stress or adversity hits
PO 3.6	Identified relationship support determines whether engaging in training may be beneficial for themselves

### Step 3: Evidence-Based Behaviour Change Techniques and Practical Applications

The change objectives developed for each of the performance objectives in step 2 were placed in a new table. In step 3 these change objectives were matched to evidence-based BCT’s (10) in Table 2. The table mentions each specific BCT as well as the psychological theories they come from. For each BCT, the programme team then constructed practical applications that were to be implemented in the programme. The result is a line-by-line theoretical blueprint for the programme.

**TABLE 2:** Combined table for behavioural outcome 1 (engages in regular activities that are known to increase wellbeing and mental health of the individual), behavioural outcome 2 (practices resilience activities to prepare for times of stress and adversity), and environmental outcome 1 (relationship supports individual in striving for more wellbeing and resilience).

	PO	Change objectives	BCT	Theory	Practical applications
	**Demonstrates understanding of (higher order comprehension)**				
	K1.1a	– Mental health, wellbeing and mental illness	1. *Persuasive Communication (6.5)* 2. *Elaboration (6.6)* 3. *Using Imagery (6.6)* 4. *Arguments (6.9)* 5. *Repeated Exposure (6.9)* 6. *Cultural Similarity (6.9)*	1. *CPM, ELM, DIT* 2. *IPT, ELM* 3. *TIP* 4. *CPM, ELM* 5. *TL* 6. *CPM*	1. *An image/video/text that displays the relationship of mental health, wellbeing & mental illness. Use real-world examples throughout to make it easier to comprehend.* 2. *Provide facts about psychological health and how it affects everyone on a day-to-day basis (includes definitions) and getting participants to relate this to their own psychological health.* 3. *Use physical fitness and physical health as analogies to the relationship between mental illness and wellbeing.* 4. *Discuss the evidence that underpins dual-factor models, wellbeing and mental illness individually (includes definitions) and get participant to understand the differences and how these differences apply to their own mental health.* 5. *Show visualisation of mental health, wellbeing and mental illness repeatedly throughout the programme and reaffirm notion that psychological health is relevant to all of us irrespective of clinical symptoms during different sessions.* 6. *Where possible use examples, statistics and support material that is relevant to the participant group (e.g., students, workforces, etc.).*
	K1.2b	– The way different intervention types influence psychological health	1. *Persuasive Communication (6.5)* 2. *Active learning (6.5)* 3. *Elaboration (6.6)* 4. *Using Imagery (6.6)*	1. *CPM, ELM, DIT* 2. *ELM, SCT* 3. *IPT, ELM* 4. *TIP*	1. *Provide a brief explanation how different intervention types improve relevant outcomes, and what their scientific evidence is (on a high level).* 2. *Ask participants to guess which intervention type across the board influences most mental health outcomes.* 3. *Provide explanatory videos that shed insight into background mechanics of interventions (e.g., video on epigenetic influences on our life).* 4. *Use examples of healthy pot-plants versus garden-grown plants to explain that psychological health can be influenced by genetics and environment, and that this impacts intervention impact.*
	K1.2c	– Current evidence-status for individual intervention types on improving psychological health	1. *Persuasive Communication (6.5)* 2. *Advance Organisers (6.6)* 3. *Arguments (6.9)*	1. *CPM, ELM, DIT* 2. *TIP* 3. *CPM, ELM*	1. *Give a general evidence overview of the main intervention types and their impact on psychological health. Include information about the benefits of improving psychological health for everyone.* 2. *Use symbols, way of organising and colours to indicate evidence status for individual techniques.* 3. *Provide high level summary of evidence for each activity proposed, split per main target outcome. Provide access to background research and get participants to realise that different types of interventions have a different impact depending on the outcome and other characteristics.*
	K1.3a	– Personal characteristics that have a proven association with *psychological health*	1. *Persuasive Communication (6.5)* 2. *Elaboration (6.6)*	1. *CPM, ELM, DIT* 2. *IPT, ELM*	1. *Briefly list scientific evidence on personal characteristics that impact psychological health and their relation.* 2. *Use videos (e.g. on impact of stress) to indicate that biopsychosocial influences all play a role in how we feel.*
	K1.3b	– Personal characteristics that are known to *impact interventions* for psychological health	1. *Persuasive Communication (6.5)* 2. *Advance Organisers (6.6)*	1. *CPM, ELM, DIT* 2. *TIP*	1. *Provide explanation on differential response for different people and the need for one-size fits all solutions based on background research. Place in larger frame of resources and challenges.* 2. *Create groupings were possible to make information processing easier (e.g., psychological, health, interpersonal).*
	K1.4a	– The presence of fluctuations in psychological health outcomes throughout life	1. *Persuasive Communication (6.5)* 2. *Active learning (6.5)* 3. *Elaboration (6.6)* 4. *Using Imagery (6.6)*	1. *CPM, ELM, DIT* 2. *ELM, SCT* 3. *IPT, ELM* 4. *TIP*	1. *Provide high-level scientific evidence on daily mood and wellbeing fluctuations.* 2. *Embed measurement and reporting on mental health into programme, showing fluctuations over time.* 3. *Stimulate conversation between attendees regarding observed fluctuations.* 4. *Use the example of the weather and climate or alternatively progress in sports and physical health to explain how individual fluctuations in mental health work.*
	K1.4b	– Psychological health over the life-course requires a committed approach	1. *Active learning (6.5)* 2. *Modelling (6.5)* 3. *Using Imagery (6.6)*	1. *ELM, SCT* 2. *SCT, TL* 3. *TIP*	1. *Ask participants to reflect on whether anything that plays a big role in life comes overnight.* 2. *Get trainers to talk about their own life course approach to building mental health.* 3. *Use analogy of marathon (or other big accomplishment) to explain that this journey is long. Use imagery to explain it is not a linear trajectory and that we will have wins and losses in this journey.*
	K1.5b	– Psychological health consists of different personal outcomes	1. *Arguments (6.9)* 2. *Advance Organisers (6.6)*	1. *CPM, ELM* 2. *TIP*	1. *Use definitions on mental health and distinct sub-outcomes to indicate the role that each outcome plays.* 2. *Break up definitions and guide participants through each relevant sub-outcome to create understanding of differences.*
	K1.6d	– Importance of social relationships	1. *Persuasive communication (6.5)* 2. *Repeated Exposure (6.9)*	1. *CPM, ELM, DIT* 2. *TL*	1. *Provide clear rationale for the importance of social environment in our mental health and wellbeing, by referring to theories, evidence and examples throughout the programme.* 2. *Repeat importance of social environment throughout sessions.*
	K1.6.1a	– Scope of the programme	1. *Persuasive communication (6.5)* 2. *Repeated Exposure (6.9)*	1. *CPM, ELM, DIT* 2. *TL*	1. *Clearly articulate that the main focus of the programme is to build mental health, not to focus on mental illness. Explain the difference between those concepts clearly.* 2. *Repeat information on scope throughout course.*
	K1.6.1b	– Types of challenges that cannot be altered by a psychological skills training	1. *Persuasive communication (6.5)*	1. *CPM, ELM, DIT*	1. *Provide clear rationale for focus on mental health and not mental illness or other problems that may contribute to problems in mental health (e.g. housing, finances) from start of the programme. Point to presence of external influences that can shape mental health and indicate that improvement potential differs per person and their environment.*
	K1.7b	– The role that values play in steering positive human behaviours	1. *Persuasive Communication (6.5)* 2. *Active Learning (6.6)* 3. *Discussion (6.6)* 4. *Using Imagery (6.6)* 5. *Arguments (6.9)*	1. *CPM, ELM, DIT* 2. *ELM, SCT* 3. *ELM* 4. *TIP* 5. *CPM, ELM*	1. *Give insight into scientific evidence on role of values and life goals, and how values relate to positive health behaviours and goal attainment. Share different theories that they play a role in (e.g. ACT).* 2. *Get participants to identify their own values in past behaviour and link this to new goals they set related to their mental health and wellbeing.* 3. *Talk through individual values with other participants.* 4. *Ask participants to think about a time when they relied on their values to steer positive behaviour.* 5. *Provide scientific arguments for the role of values in positive human behaviour and indicate that everybody has certain values they hold, which they can use to influence their mental health.*
	K1.8a	– The importance of developing a strategy of sufficient intensity	1. *Persuasive Communication (6.5)* 2. *Elaboration (6.6)* 3. *Using Imagery (6.6)* 4. *Arguments (6.9)*	1. *CPM, ELM, DIT* 2. *IPT, ELM* 3. *TIP* 4. *CPM, ELM*	1. *Provide scientific evidence on developing a strategy of sufficient intensity across different outcomes. Rhetorically, ask participants to think of the last time they nailed an important life skill (driving a car, having sex, learning a new language) in one go.* 2. *Ask participants to place the importance of investing in their mental health by developing a strategy of sufficient intensity in the context of their own life, motivations, and values.* 3. *Use physical activity as an example to explain how it is important to train sufficiently when trying to run a marathon (your life). Alternatively use medicine as an example.* 4. *Provide the results of the systematic review to develop scientific trust in presented findings and need to fully commit to developing a strategy.*
	K.1.8.1	– How barriers can influence successful execution of the psychological health strategy	1. *Persuasive Communication (6.5)* 2. *Repeated Exposure (6.9)*	1. *CPM, ELM, DIT* 2. *TL*	1. *Provide information on various barriers, both theoretical and from the trainer’s own experience, and how participants need to be aware of them to be successful in executing the strategy.* 2. *Repeat information and cues on reflecting on barriers throughout the programme (e.g. during reflection on how previous weeks went at start of each session).*
	K1.9	– Improving or maintaining psychological health requires an ongoing commitment	1. *Persuasive Communication (6.5)* 2. *Using Imagery (6.6)* 3. *Arguments (6.9)*	1. *CPM ELM, DIT* 2. *TIP* 3. *CPM, ELM*	1. *Provide information on typical trajectory of improvement, indicating that sometimes a deterioration may happen before positive change occurs.* 2. *Use analogies of sports or other areas to indicate that improvement comes with ups and downs.* 3. *Provide realistic optimism as an approach to indicating that success can happen despite failures, but requires ongoing commitment.*
	K1.10.1a	Different people require different strategies to see effective change in outcomes	1. *Persuasive Communication (6.5)* 2. *Active Learning (6.5)* 3. *Modelling (6.5)* 4. *Using Imagery (6.6)*	1. *CPM, ELM, DIT* 2. *ELM, SCT* 3. *SCT, TL* 4. *TIP*	1. *Provide examples of difference in people responding to different strategies, both in effectiveness as well as implementation and liking.* 2. *Let people interact with their own wellbeing scores and compare changes with other participants.* 3. *Over course of the programme get participants to discuss their personal strategy, showing that individuals will gravitate to and need different activities depending on their life’s circumstances* 4. *Provide analogy of the way psychologists and counsellors work in finding ways to work with their clients.*
	K1.10.3a, K2.8.2b, K2.11.3a	Personal situation/outcome that warrants professional support	1. *Persuasive Communication (6.5)* 2. *Using Imagery (6.6)* 3. *Consciousness Raising (6.7)* 4. *Personalise Risk & framing (6.7)* 5. *Repeated Exposure (6.9)*	1. *CPM, ELM, DIT* 2. *TIP* 3. *HBM, PAPM, TTM* 4. *PAPM* 5. *TL*	1. *Provide scientific evidence on differing levels of symptoms and the effectiveness of different techniques on dealing with symptoms, leading participants to understand they can’t take everything on themselves.* 2. *Use analogy of going to the GP and ED for severe physical illness and the need to go to the pharmacy yourself when it is minor. Use analogy of various fires to indicate that you can deal with some fires but not with all (i.e. you need to call the fire department).* 3. *Make it clear that not understanding about severity of symptoms can impact them in forming an effective strategy and thus in being able to improve their mental health.* 4. *Provide information on the long-term impact of not acting on their mental health, and how this may impact the participant’s future* 5. *Repeat symptoms message and need to know which symptoms can be manageable or not throughout programme.*
	K1.10.4	Adjusting a strategy may lead to better outcomes over the life-course	1. *Persuasive Communication (6.5)* 2. *Using Imagery (6.6)* 3. *Repeated Exposure (6.9)*	1. *CPM, ELM, DIT* 2. *TIP* 3. *TL*	1. *Ensure that the participant knows that creating and tweaking a strategy is the core principle of the programme and generally underpins growth.* 2. *Provide analogies such as sports and adjusting training to improve outcomes.* 3. *Reinforce the message each session by allowing participants to experiment with their strategy.*
	K2.1b	– Resilience as an outcome	1. *Persuasive Communication (6.5)* 2. *Active learning (6.5)* 3. *Discussion (6.6)* 4. *Elaboration (6.6)* 5. *Arguments (6.9)*	1. *CPM, ELM, DIT* 2. *ELM, SCT* 3. *ELM* 4. *IPT, ELM* 5. *CPM, ELM*	1. *Provide scientific definitions of resilience and place in context of its malleability (i.e. resilience as an outcome you can change).* 2. *Ask participants to think of a time where they felt they were resilient to stress or stressful events.* 3. *Get participants to talk through learned information on resilience and other mental health outcomes.* 4. *Get participants to reflect on concept of resilience and how this relates to their life.* 5. *Throughout programme provide scientific information on why resilience is malleable.*
	K2.2c	– How stress can lead to growth	1. *Persuasive Communication (6.5)* 2. *Modelling (6.5)* 3. *Elaboration (6.6)* 4. *Using Imagery (6.6)*	1. *CPM, ELM, DIT* 2. *SCT, TL* 3. *ELM* 4. *TIP*	1. *Provide information on scientific evidence regarding post stress growth and the positive consequences of stress* 2. *Get trainers to provide an example where they felt they went through stress and grew afterward.* 3. *Ask people to think about a time where they felt they grew after going through stress* 4. *Use bushfires as an analogue of a completely destructive force, but nature recovering afterward. Alternatively use examples of physical activity when someone suffered an injury and miraculously recovered.*
	K2.6b	That resources and barriers for resilience can change over time	1. *Persuasive Communication (6.5)* 2. *Modelling (6.5)* 3. *Using Imagery (6.6)*	1. *CPM, ELM, DIT* 2. *SCT, TL* 3. *TIP*	1. *Provide information on the transient nature of resources and barriers in one’s life and that we can actively work on them.* 2. *The trainer will provide the group with an example where their own resources and barriers shifted in life and how it impacted an outcome.* 3. *Use analogy of the life course to show that we all evolve and as a consequence our resources shift.*
	K2.8b	What stressor types can and cannot be managed individually	1. *Facilitation (6.5)* 2. *Modelling (6.5)* 3. *Using Imagery (6.6)*	1. *SCT* 2. *SCT, TL* 3. *TIP*	1. *Recommendations based on clinical cut-offs are presented within online measurement and accompanied by explanatory text. They indicate which symptom levels recommend being seen a professional* 2. *Trainer provides example from their own life in which they had to decide whether to get help or not to deal with stressors (where applicable).* 3. *Use medical or fire analogy to indicate that some events can be managed personally and some benefit from professional help.*
	K2.10b	Individual differences in capacity to deal with adversity	1. *Persuasive Communication (6.5)* 2. *Using Imagery (6.6)*	1. *CPM, ELM, DIT* 2. *TIP*	1. *Provide scientific literature that indicates individual differences in coping and their determinants (e.g., genomics video) and the role of perception in stress.* 2. *Use analogy from nature that highlights differences between people and being able to deal with stress.*
	K2.10c	That individual judgement may need to be supplemented with input from social environment	1. *Persuasive Communication (6.5)*	1. *CPM, ELM, DIT*	1. *Provide scientific evidence on important role of social support and positive relationships, as well as the environment in general. Explain the role that the mind plays in interpreting symptoms and that an outsider’s perspective can help in overcoming biases.*
	**Lists/describes (descriptive knowledge)**				
	K1.1b	– Behavioural and non-behavioural factors and outcomes that are associated with good psychological health outcomes	1. *Persuasive Communication (6.5)* 2. *Active learning (6.5)* 2. *Arguments (6.9)* 3. *Repeated exposure (6.9)*	1. *CPM, ELM, DIT* 2. *ELM, SCT* 3. *CPM, ELM* 4. *TL*	1. *List various scientifically derived behavioural and non-behavioural factors.* 2. *Ask participants to choose from a large set of associated variables and ask which ones they care about or has impacted their own personal lives.* 3. *Provide scientific evidence that indicates the role of each factor in relationship to psychological health via links to resources page.* 4. *Repeat behavioural and non-behavioural factors information throughout course.*
	K1.1c	– Positive outcomes associated with good psychological health	1. *Persuasive Communication (6.5)* 2. *Advance organisers (6.6)* 3. *Active learning (6.5)* 4. *Arguments (6.9)* 2. *Repeated Exposure (6.9)*	1. *CPM, ELM, DIT* 3. *TIP* 4. *ELM, SCT* 5. *CPM, ELM* 6. *TL*	1. *List scientific evidence to support positive outcomes of working on good psychological health.* 2. *Break the positive outcomes down into subsets of groups to facilitate better information processing.* 3. *Ask participants to choose from a large set of associated variables and ask which ones they care about or has impacted their own personal lives.* 4. *Provide scientific references that indicate their association with psychological health.* 5. *Repeat information on positive outcomes throughout sessions.*
	K1.2a	– Evidence-based psychological interventions to build psychological health	1. *Persuasive Communication (6.5)* 2. *Advance Organisers (6.6)* 3. *Active learning (6.5)*	1. *CPM, ELM, DIT* 2. *TIP* 3. *ELM, SCT*	1. *List scientific evidence on evidence-based intervention types to build psychological health.* 2. *Group interventions into different sub-types to aid in retention.* 3. *Create a short puzzle/quiz that gets people to reflect on the impact of specific interventions on specific outcomes.*
	K1.6a	– Common resources and challenges for good psychological health	1. *Persuasive Communication (6.5)* 2. *Advance organisers (6.6)* 3. *Active learning (6.5)*	1. *CPM, ELM, DIT* 2. *TIP* 3. *ELM, SCT*	1. *List scientific evidence on commonly understood resources and challenges.* 2. *Group resources and challenges into clearly understandable groups.* 3. *Ask participants to reflect on resources and challenges and their importance to the participant.*
	K1.7a	– List of motivators that drive human (health) behaviour	1. *Persuasive Communication (6.5)* 2. *Active learning (6.5)*	1. *CPM, ELM, DIT* 2. *ELM, SCT*	1. *List common motivators for health behaviour change and their scientific evidence.* 2. *Ask participants to think of their own motivators related to psychological health and refer back to these motivations throughout the course.*
	K1.7b	– Values that drive human (health) behaviour	1. *Persuasive Communication (6.5)*	1. *CPM, ELM, DIT*	1. *Provide list of values including the definition of values. Provide scientific background to values, virtues and strengths and how they lead to improved mental health.*
	K1.7c	– What a growth mindset is and how it aids in mental health improvement	1. *Persuasive Communication (6.5)* 2. *Arguments (6.9)*	1. *CPM, ELM, DIT* 2. *CPM, ELM*	1. *Provide examples on a growth mindset versus a fixed mindset and how both relate to improvements in outcomes. Focus is on malleability, not the theory per se.* 2. *Relate growth mindset back to scientific evidence on change in mental health outcomes and role of nature/nurture (e.g. via epigenetics video)*
	K1.8a	– Different activities that can be used to improve resources and offset barriers to psychological health	1. *Persuasive Communication (6.5)* 2. *Advance organisers (6.6)* 3. *Arguments (6.9)*	1. *CPM, ELM, DIT* 2. *TIP* 3. *CPM, ELM*	1. *List variety of recommended activities that are known to improve psychological health.* 2. *Group activities into easily understood topic areas (feeling, doing, communicating, thinking).* 3. *Provide scientific rationale for each individual activity and the way we currently understand they impact mental health (outcomes).*
	K1.8d	– Possible strategies they can consider to grow social connections as part of strategy	1. *Facilitation (6.5)*	1. *SCT*	1. *Provide examples and exercises that participants can use to involve their social network in the programme.*
	K1.10.3b, K2.11.3b	– Contact information for professional support	1. *Facilitation (6.5)* 2. *Repeated Exposure (6.9)*	1. *SCT* 2. *TL*	1. *Provide clear overview of professional support contacts.* 2. *Repeatedly show the contact information for professional support throughout the programme.*
	K2.1a, K2.2a	– The concept of stress and stressors, and their consequences	1. *Persuasive Communication (6.5)* 2. *Repeated Exposure (6.9)* *Elaboration (6.6)*	1. *CPM, ELM, DIT* 2. *TL* 3. *TIP, ELM*	1. *Provide the definition of stress and a variety of examples ranging from mild to big adversity, and how they impact individuals differently.* 2. *Repeat definitions throughout the course.* 3. *Explore concept of eustress and how this applies to the individual.*
	K2.1c	– Positive outcomes associated with improved resilience	1. *Persuasive Communication (6.5)* 2. *Repeated Exposure (6.9)*	1. *CPM, ELM, DIT* 3. *TL*	1. *Provide scientific evidence on positive outcomes associated with resilience.* 2. *Repeatedly frame the benefits of high resilience being a positive outcome.*
	K2.2b	– How stressors can be appraised differently and how this impacts stress levels	1. *Persuasive Communication (6.5)*	1. *CPM, ELM, DIT*	1. *Touch upon various stressors and the fact that stressors influence people differently, which partly depends on their level of severity and other variables.*
	K2.3b	– Evidence-based activities that can boost resilience	1. *Persuasive Communication (6.5)* 2. *Advance organisers (6.6)* 3. *Repeated Exposure (6.9)* 2. *Facilitation (6.5)*	1. *CPM, ELM, DIT* 2. *TIP* 3. *TL* 4. *SCT*	1. *Provide scientific evidence for list of activities that boost resilience.* 2. *Group activities into categories (feeling and thinking, doing, communicating).* 3. *Refer to different activities throughout course.* 4. *Provide overview of evidence via website or other resources.*
	K2.3c	– Positive effects associated with engaging in resilience activities	1. *Persuasive Communication (6.5)* 2. *Repeated Exposure (6.9)*	1. *CPM, ELM, DIT* 2. *TL*	1. *Provide scientific evidence on positive outcomes associated with resilience and their flow-on effects on other mental health outcomes.* 2. *Repeatedly frame the benefits of resilience and the fact that it is malleable.*
	K2.4	– Role of biology, psychology and social circumstances on resilience	1. *Persuasive Communication (6.5)* 2. *Active Learning (6.5)*	1. *CPM, ELM, DIT* 2. *ELM, SCT*	1. *Provide scientific evidence on characteristics that influence resilience (and other mental health outcomes).* 2. *Integrate knowledge by linking resilience to other videos on mental health used in the programme.*
	K2.6a	– Common resources and challenges for resilience	1. *Persuasive Communication (6.5)* 2. *Repeated Exposure (6.9)*	1. *CPM, ELM, DIT* 2. *TL*	1. *Provide scientific evidence on common resources and challenges for resilience and coping with stress.* 2. *Repeat the common resources and challenges throughout the course.*
	K2.8a	– Activities that can be used for personal resilience strategy	1. *Persuasive Communication (6.5)* 2. *Advance Organisers (6.6)*	1. *CPM, ELM, DIT* 2. *TIP*	1. *List scientific evidence on evidence-based interventions types to build resilience.* 2. *Group interventions into different sub-types to aid in retention.*
	K2.8b	– What stressor types can and cannot be managed individually	1. *Persuasive Communication (6.5)*	1. *CPM, ELM, DIT*	1. *Provide information on aspects that are in scope and out of scope to be self-managed.*
	K2.10a	– The mental and physical health symptoms associated with unhealthy reactions to stress	1. *Persuasive Communication (6.5)* 2. *Active Learning (6.5)* 3. *Repeated Exposure (6.9)*	1. *CPM, ELM, DIT* 2. *ELM, SCT* 3. *TL*	1. *List scientifically derived symptoms that can come from adversity and stress.* 2. *Use video to explain symptoms associated with stress.* 3. *Repeat information on stress symptoms throughout programme.*
	K2.11.2	– Barriers to executing psychological health strategies	1. *Active Learning (6.5)* 2. *Repeated Exposure (6.6)*	1. *IPT, ELM* 3. *TL*	1. *Ask participants to reflect on barriers that have inhibited them from executing health behaviours in the past.* 2. *Repeat the need to reflect on barriers throughout the programme.*
	**Explains (procedural knowledge)**				
	K1.5a, K2.5	– How to access psychological outcome assessment methods to build psychological profile and resilience	1. *Advance organisers (6.6)* 2. *Modelling (6.5)* 3. *Guided practice (6.11)*	1. *TIP* 2. *SCT, TL* 3. *SCT, TSR*	1. *Break up steps of accessing the platform into simple steps.* 2. *Have a trainer explain how to access the measurement tools.* 3. *Develop video that explains how to access the platform and how to conduct the measurement.*
	K1.6.1c	– Where to find help for psychological issues out of scope of the programme	1. *Advance Organisers (6.5)* 2. *Facilitation (6.5)*	1. *TIP* 2. *SCT*	1. *Create a process for participants to find information on additional out-of-scope resources.* 2. *Create overview for relevant professional support references.*
	K1.8b	– How to access activities that can be used improve psychological health	1. *Advance Organisers (6.6)* 2. *Guided practice (6.11)* 3. *Modelling (6.5)*	1. *TIP* 2. *SCT, TSR* 3. *SCT, TL*	1. *Visualise the steps of accessing the activities in a simple diagram. Use colours, ordering and symbols to guide participants to activities within the booklet.* 2. *Have trainers demonstrate how to access each activity and use it to form a strategy.* 3. *Have trainers demonstrate use of the support material to the participant.*
	**Identifies**				
	K1.6b, K1.6c, K2.6c, K2.6d	– Personal resources and barriers for their psychological health and resilience	1. *Tailoring (6.5)* 2. *Modelling (6.5)* 3. *Providing Cues (6.6)* 2. *Framing (6.7)* 3. *Public commitment (6.8)* 4. *Arguments (6.9)*	1. *TTM, PAPM, PMT, CPM* 2. *SCT, TL* 3. *TIP* 4. *PMT* 5. *TAIHB* 6. *CPM, ELM*	1. *Ask participants to reflect on personal circumstances and select different types of resources and barriers that apply to their own life.* 2. *Let trainers select types of barriers and resources that applied to their own psychological health out of list of options.* 3. *Indicate the consequence of not identifying personal resources, i.e., they can still do the course, but the results will be suboptimal.* 4. *Use a gain frame to indicate that identifying resources will lead to positives and that not identifying it will come at a cost.* 5. *Get participants to talk to other participants about their own resources and how it shaped their strategy.* 6. *Provide scientific rationale for the importance of selecting personal resources and barriers.*
	K1.8c	– Specific strategies that contribute positively to their psychological health	1. *Persuasive Communication (6.5)* 2. *Modelling (6.5)* 3. *Discussion (6.6)* 4. *Consciousness Raising (6.7)* 5. *Framing (6.7)* 6. *Public Commitment (6.8)*	1. *CPM, ELM, DIT* 2. *SCT, TL* 3. *ELM* 4. *HBM, PAPM, TTM* 5. *PMT* 6. *TAIHB*	1. *Provide scientific rationale for individual psychological strategies and when to consider them.* 2. *Have a trainer demonstrate how they selected a strategy that was matched to their own wellbeing profile.* 3. *Share one of the strategies the participant chose with another participant and explain why this was matched to their personal circumstances.* 4. *Point to consequences of not selecting strategies that relate to their own psychological health (e.g., suboptimal outcomes) and their wellbeing profile.* 5. *Use a gain frame to indicate that identifying strategies will lead to positives and that not identifying it will come at a cost.* 6. *Get participants to pledge to identify and explore tailored strategies on a weekly basis.*
	K1.8.1	– How barriers can influence successful execution of the psychological health strategy	1. *Discussion (6.6)* 2. *Consciousness Raising (6.7)* 3. *Arguments (6.9)*	1. *ELM* 2. *HBM, PAPM, TTM* 3. *CPM, ELM*	1. *Share one of the barriers the participant chose with another participant and explain how this affected their strategy.* 2. *Point to consequences of not selecting personal barriers that relate to their own psychological health (e.g., suboptimal outcomes) and their wellbeing profile.* 3. *Provide rationale for the importance of selecting personal resources and barriers.*
	K1.10.1, K2.10.1	– Personal criteria for successful execution of psychological health strategy	1. *Persuasive Communication (6.5)*	1. *CPM, ELM, DIT*	1. *Provide information on the importance of determining what success looks like, how to measure it and how to use it in the context of the programme.*
	**Demonstrates ability to**				
	S1.1, S2.1, S2.2	– Process information on psychological health	1. *Facilitation (6.5)* 2. *Set Graded tasks (6.11)*	1. *SCT* 2. *SCT, TSR*	1. *Facilitate the creation of workbooks and the opportunity to access the materials. Provide sufficient time to allow ability to master information. Ensure access to in-person resources and space to participate in the training.* 2. *Gradually build complexity of information in programme to facilitate better processing of information.*
	S1.2, S2.3, S2.4b	– To critically interpret evidence on interventions to improve psychological health	1. *Facilitation (6.5)* 2. *Guided practice (6.11)*	1. *SCT* 2. *SCT, TSR*	1. *Create access to evidence on interventions to improve psychological health in slides and course material. Transform scientific formats into laymen friendly resources. Provide access to scientific resources where possible.* 2. *Demonstrate how to interpret evidence related to personal situation. Use an example to get the participants to interpret the evidence.*
	S1.3, S1.6, S1.7, S2.4a, S2.6, S2.7	– Reflect on personal characteristics that apply to the individual	1. *Facilitation (6.5)* 2. *Provide contingent rewards (6.11)* 2. *Modelling (6.5)*	1. *SCT* 2. *TL, TSR* 3. *SCT, TL*	1. *Provide safe opportunity and space to reflect on individual characteristics without causing resistance of the individual. Create exercises that are specifically designed to reflect on personal lives.* 2. *Praise and encourage participation even though it may require people to reflect on difficult subjects.* 3. *Use trainer examples to highlight how reflection is done in the context of the programme.*
	S1.6.1	Which resources and challenges can be managed or improved by themselves	1. *Facilitation (6.5)*	1. *SCT*	1. *Use online measurement to indicate whether symptom levels are higher than should be self-managed. Get participants to link resources and challenges to symptoms level.*
	S1.10.3c	Compare the objectives of existing programme to other services	1. *Facilitation (6.5)*	1. *SCT*	1. *Provide overview of focus areas for the programme, what is in and out of scope and point to other services to handle out of scope objectives.*
	S1.7, S2.7	– Identify personal motivators to improve psychological health and resilience	1. *Tailoring (6.5)* 2. *Modelling (6.5)* 3. *Consciousness Raising (6.7)* 4. *Framing (6.7)* 5. *Discussion (6.5)* 6. *Arguments (6.9)* 7. *Facilitation (6.5)*	1. *TTM, PAPM, PMT, CPM* 2. *SCT, TL* 3. *HBM, PAPM, TTM* 4. *PMT* 5. *ELM* 6. *CPM, ELM* 7. *SCT*	1. *Ask participants to reflect on personal circumstances and motivators, and reflect on what motivates them in general life.* 2. *Let trainers demonstrate their own motivators and drivers in life.* 3. *Indicate the consequence of not identifying motivators, i.e., they can still do the course, but the results will be suboptimal.* 4. *Use a gain frame to indicate that identifying motivators will lead to positives and that not identifying it will come at a cost.* 5. *Ask participants to share their own motivators.* 6. *Provide scientific rationale for the importance of selecting personal resources and barriers* 7. *Provide resources to reflect on personal motivators, e.g., self-reflection exercises*
	S1.5b, S2.5b	– Ability to interpret scores on psychological health assessment methods	1. *Facilitation (6.5)*	1. *SCT*	1. *Facilitate access to assessment criteria and their interpretation to ensure the participant understand current profile, without the need for a professional to help explore scores. Provide clear explanation via slides on how to interpret scores when in-person training is taught.*
	S1.8d	– Match intervention activities to personal needs	1. *Tailoring (6.5)* 2. *Facilitation (6.5)*	1. *TTM, PAPM, PMT, CPM* 3. *SCT*	1. *Create tailored recommendations for interventions based on psychological profile and identified needs* 2. *Facilitate resources (online/offline) that match recommendations with intervention recommendations. Match booklet recommendations to wellbeing profile generated by platform, e.g., activity finders.*
	S1.10.1a, S2.11.1	– Reflect on whether strategy activities are leading to change	1. *Self-monitoring (6.11)* 2. *Facilitation (6.5)*	1. *TSR* 2. *SCT*	1. *Patient is prompted to keep track of use of strategy (e.g., in diary) on weekly basis and to reflect on their personal experience with the strategies.* 2. *The training will provide resources to enable reflection and self-monitoring, including access to a self-monitoring too (i.e. the online platform).*
	S1.10.2b	– Explain psychological health strategy to social supporter	1. *Modelling (6.5)* 2. *Facilitation (6.5)*	1. *SCT, TL* 2. *SCT*	1. *Use personal stories where the trainer or models explain how they talked to their relationships.* 2. *Provide a specific exercise that gets participants to share their strategy with their social supporter.*
	S1.10.3b, S2.11.3	– Reach out to professional support	1. *Facilitation (6.5)* 2. *Set graded tasks (6.11)*	1. *SCT* 2. *SCT, TSR*	1. *Provide access to support contact information in training and resources.* 2. *Tell participants to come up to trainer in case they are unsure of how to broach problems or where to go with challenges.*
	S2.10	– Recognise stress when faced with it	1. *Self-monitoring (6.11)* 2. *Facilitation (6.5)*	1. *TSR* 2. *SCT*	1. *Allow function where participant can monitor stressors over a set period when revising and checking their strategy.* 2. *Provide for self-monitoring functionality in course content as well as measurement platform. Allow participants to practice recognising stressors.*
	S2.6.1	– To identify stressors which can and cannot be managed personally	1. *Self-monitoring (6.11)* 2. *Facilitation (6.5)*	1. *TSR* 2. *SCT*	1. *Allow diary function where participant can monitor stressors over a monthly period when revising and checking their strategy.* 2. *Provide for self-monitoring functionality in course content.*
	S1.5a, S2.5a	Can complete psychological health assessment methods	1. *Facilitation (6.5)*	1. *SCT*	1. *Embedding the psychological health assessment as part of the training in an online environment that can be accessed with any device that adheres to modern web standards.*
	Practices				
	S1.8a, S2.8a	– The use of psychological health activities during the training	1. *Modelling (6.5)* 2. *Feedback (6.5)* 3. *Reinforcement (6.5)* 4. *Facilitation (6.5)* 5. *Guided practice (6.11)* 6. *Verbal persuasion (6.11)*	1. *SCT, TL* 2. *TL, GT, SCT* 3. *TL, SCT* 4. *SCT, TSR* 5. *SCT, TSR* 6. *SCT, TSR*	1. *The trainer displays certain activities during the training. Videos with appropriate models are embedded within the activities were possible.* 2. *The trainer provides feedback on execution or practice of tasks.* 3. *The trainer provides praise to general group after completing an activity.* 4. *Individual practices activities during training time, guided by explanations or by modelling activities by the trainers. Examples are provided in course material.* 5. *Participants are asked to highlight tasks they have difficulty with and can act as models in a simulation to both get feedback and provide information to other participants.* 6. *Provide information about the fact that all skills are designed to be used by anyone, regardless of their individual knowledge and skill level.*
	S1.8b, S2.8b	– The use of psychological health activities after training	1. *Modelling (6.5)* 2. *Feedback (6.5)* 3. *Reinforcement (6.5)* 4. *Facilitation (6.5)* 5. *Guided practice (6.11)* 6. *Verbal persuasion (6.11)*	1. *SCT, TL* 2. *TL, GT, SCT* 3. *TL, SCT* 4. *SCT, TSR* 5. *SCT, TSR* 6. *SCT, TSR*	1. *Trainer gives examples on how they used the activities outside of the training. They provide examples of how they embedded the activities within their own life.* 2. *The trainer provides feedback on execution or practice of tasks at subsequent sessions.* 3. *The trainer provides praise after successfully practicing the activities during previous weeks. Emails are sent as reinforcement.* 4. *Provide course materials and activity sheets to allow practicing at home. Provide tips and tricks on how to embed activities within their own life.* 5. *Implementation within normal life of skills is stimulated.* 6. *The trainer provides examples of how they practiced skills within their normal life.*
	S1.8.1b, S2.11.2	Develops strategy to overcome barriers to using psychological health activities	1. *Reinforcement (6.5)* 2. *Facilitation (6.5)* 3. *Planning coping responses (6.11)*	1. *TL, SCT* 2. *SCT, TSR* 3. *ATRPT, TSR*	1. *Provide verbal reinforcement to continue to work through barriers that are encountered during the programme* 2. *Provide resources to allow participants to reflect on barriers.* 3. *Provide potential examples that participants can consider when devising a plan to overcome the barriers. Provide exercise that gets participants to reflect on future barriers.*
	S1.8c	Develops competency in use of psychological health activities in day-to-day life	1. *Reinforcement (6.5)* 2. *Facilitation (6.5)* 3. *Guided practice (6.11)*	1. *TL, SCT* 2. *SCT, TSR* 3. *SCT, TSR*	1. *Provide praise throughout the course when activities and exercises are completed. Allow reflection after each session to reinforce progress.* 2. *Provide resources that permit rehearsing activities.* 3. *The trainer selects specific skills and demonstrates it in the course. The coursebook refers to multimedia context that further explains skills so the participant can practice.*
	S1.9b	Recognises when to use specific activities to improve psychological health	1. *Guided practice (6.11)* 2. *Facilitation (6.5)* 3. *Self-monitoring (6.11)*	1. *SCT, TSR* 2. *SCT* 3. *TSR*	1. *Participant practices recognising symptoms or activities that warrant them using their strategy.* 2. *The course content will prompt participants to match activities to life events, stressors and outcomes (symptoms).* 3. *The course material provides ability to reflect on specific triggers that warrant the use of activities.*
	Express positive attitude				
	BI1.1, BI1.2, BI2.2	- Toward learning about psychological health and learning about different interventions to build psychological health	1. *Belief selection (6.5)* 2. *Consciousness Raising (6.7)* 3. *Personalise Risk (6.7)* 4. *Framing (6.7)* 5. *Self-reevaluation (6.7)* 6. *Environmental reevaluation (6.7)*	1. *TPB, RAA* 2. *HBM, PAPM, TTM* 3. *PACM* 4. *PMT* 5. *TTM* 6. *TTM*	1. *Ask participants to determine why the training would have personal value and follow this with examples of benefits of attending the training in each potential category.* 2. *Indicate the scientific evidence on the importance of good psychological health for individuals, their work, their family and other drivers. Provide evidence on malleability of psychological health.* 3. *Relate information on psychological health back to the participant’s personal situation.* 4. *Explain that not participating in the training will lead to a loss, whereas participating will lead to a gain.* 5. *Ask participants to reflect on how their life would benefit if they were to learn about psychological health.* 6. *Ask participants why participating in the training would be beneficial for their loved ones.*
	BI1.3, BI2.4	– Toward interrogating personal characteristics and needs	1. *Consciousness Raising (6.7)* 2. *Modelling (6.5)*	1. *HBM, PAPM, TTM* 2. *SCT, TL*	1. *Provide scientific information and personal examples to highlight the benefits of interrogating personal needs. Indicate that this leads to better results in the programme.* 2. *Let trainers provide examples of where reflection on personal needs led to benefits for the trainer.*
	B1.5a, BI2.5a	– Toward validity of psychological health assessment methods	1. *Persuasive Communication (6.5)* 2. *Consciousness Raising (6.7)*	1. *CAPM, ELM, DIT* 2. *HBM, PAPM, TTM*	1. *Provide scientific evidence on reliability and validity of assessment tools and their use in everyday life. Explain how they are used in other health settings.* 2. *Provide overview of benefits of using the assessment tools in relationship to the course and their general life.*
	B1.5b, BI2.5b	– Toward measuring their psychological health profile over time	1. *Verbal Persuasion (6.11)* 2. *Consciousness Raising (6.7)* 3. *Using Imagery (6.6)*	1. *SCT, TSR* 2. *HBM, PAPM, TTM* 3. *TIP*	1. *Provide information on fluctuations of outcomes throughout life and the need to repeat measurements.* 2. *Provide overview of benefits of using the assessment tools in relationship to the course and their general life.* 3. *Use analogy of physical health or weight to show how we fluctuate over time. Or alternatively use the weather and climate analogy.*
	BI1.7a, BI2.7a	– That psychological health *training* will be beneficial	1. *Persuasive Communication (6.5)* 2. *Consciousness Raising (6.7)*	1. *CAPM, ELM, DIT* 2. *HBM, PAPM, TTM*	1. *Provide evidence on how training can lead to important benefits in people’s life across a number of domains.* 2. *Get participants to reflect on why training will be relevant to their own personal life and motivators.*
	BI1.8a, BI2.8a	– That psychological health *strategy* will be beneficial	1. *Consciousness raising (6.7)* 2. *Self-reevaluation (6.7)*	1. *HBM, PAPM, TTM* 2. *TTM*	1. *Provide information on benefits of executing psychological health activities.* 2. *Encourage the participants to think about the benefits of enacting the strategy and what personal loss it would be to not complete the strategy.*
	BI1.6	– Expanding social support	1. *Consciousness raising (6.7)* 2. *Self-reevaluation (6.7)*	1. *HBM, PAPM, TTM* 2. *TTM*	1. *Provide information on the importance of social support for our mental health and wellbeing.* 2. *Encourage participants to reflect on the social supporters that have been there for them in important times, and how they can help them in the future*
	BI2.6.1	– Toward reaching out or using other mental health and wellbeing services	1. *Consciousness raising (6.7)*	1. *HBM, PAPM, TTM*	1. *Get participants to reflect on how professional services play an important role in other health areas, and how they do the same for mental health and wellbeing.*
	B1.10.2a, B1.10.4, BI2.11.4	– Toward effectiveness of re-evaluated strategy after implementation	1. *Reattribution training (6.11)* 2. *Persuasive communication (6.5)* 3. *Direct Experience (6.9)*	1. *ATRP, TSR* 2. *CAPM, ELM, DIT* 3. *TL*	1. *Aim to get the participant to relate unsuccessful strategies to external events, not the individual participant.* 2. *Provide scientific information on the need for trial and error and personalisation.* 3. *By integrating experimentation throughout the course, the participant will become more confident that the strategy becomes stronger over time.*
	B.1.10.3, BI2.11.3	– Toward the use of professional support	1. *Belief selection (6.5)* 2. *Personalise Risk (6.7)* 3. *Framing (6.7)* 4. *Self-reevaluation (6.7)* 5. *Persuasive Communication (6.5)*	1. *TPB, RAA* 2. *PACM* 3. *PMT* 4. *TTM* 5. *CAPM, ELM, DIT*	1. *Provide positive messages toward the effectiveness of professional support.* 2. *Provide information on consequences of not using professional support in terms of losses and gains.* 3. *Use gain and loss frames when promoting use of professional support.* 4. *Try and stimulate participant to think of themselves with and without the use of professional support and the consequences it may have.* 5. *Provide scientific data on the benefit of professional services when symptoms become too much.*
	B1.2b, BI2.3b	Judges that psychological health and resilience is malleable *via* different interventions	1. *Persuasive communication (6.5)* 2. *Modelling (6.5)*	1. *CPM, ELM, DIT* 2. *SCT, TL*	1. *Provide scientific information and personal anecdotes of how psychological training can improve mental health.* 2. *Use videos etc. of role model or experiences of the trainer to create belief in malleability of psychological health.*
	BI1.7b, BI2.7b	Beliefs that personal identity is congruent with focus of psychological health training	1. *Persuasive communication (6.5)* 2. *Normative influence (6.10)*	1. *CAPM, ELM, DIT* 2. *TPB, RAA, SCT*	1. *Provide scientific information and personal anecdotes of how psychological training can improve mental health for different people.* 2. *Provide information on approval of other participants or people who are similar to the target group via testimonials and interaction with other participants.*
	BI1.7c, BI2.7c	Relates personal motivators to importance of engaging in psychological health activities	1. *Consciousness Raising (6.7)* 2. *Personalise Risk (6.7)* 3. *Framing (6.7)* 4. *Self-reevaluation (6.7)*	1. *HBM, PAPM, TTM* 2. *PACM* 3. *PMT* 4. *TTM*	1. *List different motivator types and provide information on why they will benefit from training* 2. *Place the benefits of engaging in health activities in relation to motivators.* 3. *Explain that not participating in the training will lead to a loss, whereas participating will lead to a gain* 4. *Ask participants to reflect on how their motivators would benefit if they were to actively participate in working on their mental health.*
	BI1.7d, BI2.7d	Demonstrate a (positive shift toward) a ‘growth’ identity	1. *Persuasive communication (6.5)* 2. *Repeated Exposure (6.6)*	1. *CPM, ELM, DIT* 2. *TL*	1. *Provide overview of growth and fixed mindsets, and the scientific evidence of adopting a growth mindset.* 2. *Repeat information on the importance of a growth mindset throughout the programme, both explicitly and implicitly.*
	BI1.8c	Beliefs they can identify social supporters within or outside of the programme	1. *Facilitation (6.5)*	1. *SCT*	1. *Provide activity that gets participants to select a supporter. Get other participants or trainers to volunteer in case someone is lacking a supportive social environment.*
	BI1.10.1	Demonstrates self-compassion in the case their strategy is not leading to desired outcomes	1. *Persuasive Communication (6.5)* 2. *Enactive Mastery Experiences* 3. *Modelling* 4. *Self-reevaluation*	1. *CPM, ELM, DIT* 2. *SCT, TSR* 3. *SCT* 2. *TTM*	1. *Explain concept of self-compassion and how it relates to self-criticism. Practice self-compassion strategies as part of the fundamental principles in the programme.* 2. *Get participant to practice self-compassion in another related area and refer to the need for self-compassion at each point of experimentation.* 3. *Trainer demonstrates self-compassion throughout course.* 4. *Get participant to think of how they would improve in various life domains if they practiced self-compassion.*
	BI2.3b	Beliefs that growth can happen after stress and difficult circumstances	1. *Persuasive Communication (6.5)* 2. *Modelling*	1. *CAPM, ELM, DIT* 2. *SCT*	1. *Use examples to show that growth does not happen in linear ways. Provide scientific evidence to point to ability to grow after stress (e.g., post-adversity growth).* 2. *Trainer provides examples of themselves growing after stressful times.*
	**Expresses confidence**				
	BI1.9, BI2.9	– In implementing activities in day-to-day life	1. *Planning coping responses (6.8)* 2. *Guided practice (6.11)*	1. *ATRPT, TGDB* 2. *SCT, TSR*	1. *Ask the persons to reflect on the exact methods on how to overcome barriers and list these explicitly.* 2. *Get the participant to practice implementing the activities in day-to-day life and ask them to reflect on how the implementation is going, followed by positive reinforcement.*
	BI1.10.2b, BI2.12.2c	– In discussing psychological health strategy with social actor	1. *Modelling (6.5)*	1. *SCT, TSR*	1. *Provide exercises that get participants to practice how to broach the psychological health strategy with social actor. Where possible let the trainer model this out and share between participants.*
	BI2.10a	– In effectiveness of strategy in dealing with stress	1. *Persuasive communication (6.5)*	1. *CPM, ELM, DIT*	1. *Provide evidence-based information about the merit of psychological interventions in dealing with adversity and highlight its limitations. Provide information on the fact that stress is often result of our perception and provide examples of activities that are specifically beneficial to work on stress (e.g. via activity finders).*
	BI2.10b	– Usefulness of reaching out to health professional when needed	1. *Persuasive communication (6.5)* 2. *Framing (6.7)*	1. *CPM, ELM, DIT* 2. *PMT*	1. *Provide information about the merit of professional interventions in dealing with adversity and highlight its limitations.* 2. *Provide a gain frame when discussing that professional support has merit and that not reaching our leads to a loss. Where the setting permits, ask participants to share positive experiences with professional support.*
	BI2.8c	– That certain stressors can be managed personally	1. *Persuasive communication (6.5)* 2. *Belief selection (6.5)*	1. *CPM, ELM, DIT* 2. *TPD, RAA*	1. *Provide scientific information on ability to self-manage certain stressors and their intensity level.* 2. *Strengthen belief that individual has capacity to manage certain stressors by designing an activity that targets values and resources.*
	BI2.2a	Accepts that stressors are part of everyday life	1. *Persuasive communication (6.5)* 2. *Active Learning (6.5)*	1. *CPM, ELM, DIT* 2. *ELM, SCT*	1. *Provide information on common stressors and their impact throughout the life course.* 2. *Ask participants to reflect on which stressors might apply their own life and how they have an impact on their wellbeing and mental health.*
	BI2.2b	Accepts that stressors can be mitigated against by developing a strategy to cope	1. *Persuasive communication (6.5)* 2. *Modelling (6.5)*	1. *CPM, ELM, DIT* 2. *SCT, TL*	1. *Provide information on how we can deal with a number of stressor types.* 2. *Let trainer provide an example on how they mitigated a stressor by using an activity from the strategy.*
	**Develops**				
	GB1.4	– Commitment to actively construe a strategy to build psychological health	1. *Planning coping responses (6.8)* 2. *Public commitment (6.8)* 3. *Facilitation (6.5)* 4. *Direct Experience (6.9)*	1. *ATRPT, TGDB* 2. *TAIHB* 3. *SCT* 4. *TL*	1. *Develop a clear list of barriers and enablers that can help hinder or improve the commitment to set a strategy.* 2. *Ask person to commit to sharing the formulation of their strategy to fellow participants, their social network and the trainers, throughout the programme.* 3. *Provides activities that allow the development of the strategy including the development of habits, e.g. via formation of habit statements.* 4. *Get participants to develop a strategy over the course of 5 weeks and experience the process of gradual improvement and benefit.*
	GB1.8, GB2.8	– Personal goals for the implementation of strategy in day-to-day life	1. *Set graded tasks (6.11)* 2. *Planning Coping Responses (6.8)* 3. *Facilitation (6.5)*	1. *SCT, TSR* 2. *ATRPT, TGDB* 3. *SCT*	1. *Develop simple and easy subtasks that lead to the creation of personal goals. Build these goals in complexity over time.* 2. *Get participants to think about the necessary steps to attain a goal and barriers that may come up.* 3. *Provide exercise to set goals at each stage of the programme. Relate goals to values where possible.*
	**Monitors**				
	GB1.6, GB2.6a	– Personal resources and challenges to psychological health	1. *Facilitation (6.5)* 2. *Self-monitoring of behaviour (6.11)* 3. *Persuasive communication (6.5)*	1. *SCT* 2. *TSR* 3. *CAPM, ELM, DIT*	1. *Provide ability to monitor resources and challenges within course material on weekly basis (via self-reflection exercises). Provide resources to allow participant to self-monitor beyond course.* 2. *Stimulate self-monitoring throughout the course and provide praise/feedback on various moments in course* 3. *Provide scientific arguments for why monitoring resources and challenges frequently is required.*
	GB1.10.1a	– Adherence to psychological health strategy	1. *Facilitation (6.5)* 2. *Self-monitoring of behaviour (6.11)*	1. *SCT* 2. *TSR*	1. *Provide ability to monitor progress in strategy throughout course material. Provide resources to allow participant to self-monitor beyond course.* 2. *Stimulate self-monitoring throughout the course and provide praise/feedback on various moments in course.*
	GB1.10.1b	– Training goal attainment	1. *Facilitation (6.5)* 2. *Self-monitoring of behaviour (6.11)*	1. *SCT* 2. *TSR*	1. *Provide ability to monitor training goal attainment throughout course material. Provide resources to allow participant to self-monitor beyond course.* 2. *Stimulate self-monitoring throughout the course and provide praise/feedback on various moments in course.*
	GB1.9, GB2.9	Schedules time to engage in psychological health activities to ensure goal attainment	1. *Planning Coping Responses (6.8)* 2. *Goal-setting (6.11)* 3. *Implementation Intentions (6.8)* 4. *Facilitation* 5. *Nudging (6.5)*	1. *ATRPT, TGDB* 2. *GST, TSR* 3. *TGDB, TAIBB* 4. *SCT* 5. *TAIHB*	1. *Set up plan for implementation into daily life and think of ways to improve this implementation.* 2. *Participant develops a goal to engage in participating in psychological health activities.* 3. *Provide space to write down implementation intention (habit statements) to create time for enacting strategy.* 4. *Provide calendar in training to schedule activities.* 5. *Teach participants to set prompts and nudges for training. Build in reminders.*
	GB1.10.2a, GB2.11.2a	Recognises ineffective elements of personal wellbeing strategy	1. *Facilitation (6.5)* 2. *Self-monitoring of behaviour (6.11)*	1. *SCT* 2. *TSR*	1. *Construct exercise that allows people to identify and think about ineffective elements of their strategy.* 2. *Get participants to actively monitor behaviour throughout the training.*
	GB1.10.2b, GB2.11.2b	Identifies new activities to be included in strategy	1. *Facilitation (6.5)*	1. *SCT, TSR*	1. *Construct exercise that allows people to identify new activities throughout training. Allow participants the space to reflect and recreate their strategy.*
	GB1.10.4	Adjust personal goal for strategy implementation in day-to-day life	1. *Tailoring (6.5)* 2. *Facilitation (6.5)* 3. *Personalise risk (6.7)*	1. *TTM, PAPM, PMT, CPM* 2. *SCT* 3. *PAPM*	1. *Allow participants to reflect on their personal strategy and adjust their goals in line with learnings over the training course.* 2. *Ensure that the programme material has a spot where participants can revise their goal.* 3. *Write information that informs the participant about risks of not performing adjustment of goal to implement strategy into life.*
	GB2.6b	Reflects on potential future resources and challenges to resilience	1. *Persuasive communication (6.5)* 2. *Facilitation (6.5)*	1. *CPM, ELM, DIT* 2. *SCT*	1. *Provide information on importance of identifying resources and challenges in the future.* 2. *Provide specific reflection exercise to identify future resources and challenges.*
	GB2.10	Reflect on personal life to determine presence of stressors or adversity	1. *Reinforcement (6.5)* 2. *Facilitation (6.5)*	1. *TL, SCT* 2. *SCT*	1. *Provide encouragement for participants to reflect on their personal life to determine presence of stressors or adversity.* 2. *Construct exercise that gets people to reflect on personal life and presence of stressors and adversity.*
	GB2.11.1a	Recall personal psychological health and resilience strategy	1. *Facilitation (6.5)*	1. *SCT*	1. *Provide space to reflect on strategy at each week and offer resources to continue doing this after the programme has finished.*
	GB1.11.1b	Monitors training goal attainment	1. *Repeated exposure (6.9)* 2. *Self-monitoring (6.11)*	1. *TL* 2. *TSR*	1. *Repeatedly prompt participants to monitor their goal attainment throughout course* 2. *Facilitate ability to reflect on goal attainment via reflection exercises.*
	GB2.11.1c	Review personal resilience plan at regular intervals	1. *Self-monitoring (6.11)*	1. *TSR*	1. *Monitor progress throughout the course by providing exercises at regular interval that prompts them to investigate the active strategy components.*
	GB2.11.c	Compares resilience and psychological health scores from before until after adversity	1. *Facilitation (6.5)*	1. *SCT*	1. *Allow access to online measurement report that gets participants to compare their scores from before and after the training.*
	SOC1.6a, SOC2.6a	Investigates social support for implementation of psychological health strategy	1. *Facilitation (6.5)* 2. *Persuasive communication (6.5)* 3. *Shifting perspective (6.9)* 4. *Info on normative approval (6.10)*	1. *SCT* 2. *CPM, ELM* 3. *TSD* 4. *TPB, RAA, SCOT*	1. *Provide opportunity for participant to reflect on social support by creating an exercise in programme* 2. *Provide scientific evidence on importance of social support in mental health and wellbeing.* 3. *Ask participants to consider why the social supporter would think it is important to be engaged in the participant’s psychological health.* 4. *Provide insight into the fact that social supporters are never far away, even in the case of social isolation (e.g., other training participants).*
	SOC1.6b, SOC2.6b	Determines influence of social identity to form psychological health strategy	1. *Persuasive Communication (6.5)* 2. *Individualisation (6.5)* 3. *Facilitation (6.5)*	1. *CPM, ELM* 2. *TTM* 3. *SCT*	1. *Provide scientific evidence on the role of social identity in promoting or inhibiting behaviour that promoted psychological health.* 2. *Allow participant to reflect on their own situation and question their own social identity.* 3. *Provide opportunity within working book to reflect on role of social identity.*
	SOC1.8, SOC 2.8	Involves social support in development of psychological health strategy	1. *Facilitation (6.5)* 2. *Consciousness raising (6.7)* 3. *Repeated exposure (6.7)* 4. *Personalise risk and Framing (6.7)*	1. *SCT* 2. *HBM, PAPM, TTM* 3. *TL* 4. *PAPM*	1. *Provide resources that are targetting social support and provide instructions to the participant on how to engage the social supporter.* 2. *Provide scientific information on why support from social actor is beneficial.* 3. *Repeat information on the important role of a social support network throughout course.* 4. *Provide information on the risk of not involving social support.*
	SOC1.10.1, SOC2.11.1	Communicates with social relationships whether positive changes can be noted	1. *Verbal persuasion (6.11)* 2. *Modelling (6.5)* 3. *Public Commitment*	1. *SCT, TSR* 2. *SCT, TL* 3. *TAIHB*	1. *Participants are encouraged to think that talking to social support is within their capability.* 2. *Provide example on how to broach the conversation with social support.* 3. *Stimulate pledge to discuss changes with social supporter by including it as an exercise in the programme.*
	SOC.1.10.2a, SOC2.11.2a	Schedule time to engage with social actor	1. *Goal setting (6.11)* 2. *Implementation intentions (6.8)*	1. *GST, TSR* 2. *TGDB, TAIHB*	1. *Provide exercise that actively gets participants to schedule time to discuss the programme.* 2. *Include implementation intention exercise in booklet that prompts participants to set a time to engage with social actor*
	**Describes**				
	K3.1a, K3.4	– Mental health and the positive effects of engaging in psychological health training	1. *Elaboration (6.6)* 2. *Using Imagery (6.6)* 3. *Arguments (6.9)* 4. *Facilitation (6.5)*	1. *IPT, ELM* 2. *TIP* 3. *CPM, ELM* 4. *SCT*	1. *Participants provides can provide facts about psychological health, its prevalence and how it affects everyone on a day-to-day basis (includes definitions).* 2. *Participants can use physical fitness and physical health as analogy to relationship between MI and Wellbeing* 3. *Participant can talk about the scientific evidence that underpins the programme and point out this information in the programme material.* 4. *Guide participants to the programme material that allows them to gain an understanding of the positive effects of training. Create a resource for the social supporter.*
	K3.1b	– The components of the training participant’s psychological health strategy	1. *Facilitation (6.5)* 2. *Direct Experience (6.9)*	1. *SCT* 2. *TL*	1. *Get training participant to write down what their strategy is about and provide an exercise that lists out the training components, which the participant can show to the supporter.* 2. *Supporter is actively involved in the strategy by practicing various evidence based mental health activities with the training participant.*
	K3.1c	Professional support contact information	1. *Facilitation (6.5)*	1. *SCT*	1. *Provide resource for social supporter that lists professional support numbers, and explains how, when and why to reach out to them.*
	K3.3	– Strategy activities of individual that benefit from social supports engagement	1. *Facilitation (6.5)* 2. *Direct Experience (6.9)*	1. *SCT* 2. *TL*	1. *Get training participant to write down what their strategy is about and provide an exercise that lists out the training components, which the participant can show to the supporter.* 2. *Participant is actively involved in the strategy by practicing various evidence based mental health activities with the training participant.*
	K3.6	*Understands that they can access measurement and training themselves*	1. *Persuasive Communication (6.5)* 2. *Facilitation (6.5)*	1. *CAPM, ELM, DIT* 2. *SCT*	1. *Create information material which indicates how to access the training and the measurement platform.* 2. *Create support material for training participant to hand out to supporter.*
life about Capabilities & identity	**Express positive attitude**				
	BI3.1	– That engaging in psychological activities will be beneficial to individual’s psychological health	1. *Persuasive Communication (6.5)* 2. *Consciousness Raising (6.7)* 3. *Direct Experience (6.9)*	1. *CAPM, ELM, DIT* 2. *HBM, PAPM, TTM* 3. *TL*	1. *Participant to provide information on how training can lead to important benefits in people’s life across a number of domains in easy-to-understand resource.* 2. *Get social supporter to reflect on why training would be relevant to their own personal life and motivators, and whether they have seen other people improve after actively working on their mental health.* 3. *Get social supporter directly involved in activities, thereby aiming to see noticeable changes in both participant and themselves.*
	BI3.2a	That reflection on personal psychological health profile will be beneficial to themselves	1. *Consciousness raising (6.7)* 2. *Persuasive Communication (6.5)* 3. *Framing (6.7)*	1. *HBM, PAPM, TTM* 2. *CAPM, ELM, DIT* 3. *PMT*	1. *Get participants to reflect on why they think taking regular health check-ups is a good idea, but why they don’t do this for their mental health.* 2. *Provide short resource on evidence-base behind the measurement tool.* 3. *Try and use a loss and gain frame to highlight the benefits behind the measurement tool.*
	GB3.3, 3.4	Develops personal goal for monitoring strategy use of individual	1. *Facilitation* 2. *Planning Coping Responses (6.8)* 3. *Goal-setting (6.11)* 4. *Implementation Intentions*	1. *SCT* 2. *ATRPT, TGDB* 2. *GST, TSR* 3. *–*	1. *Facilitate an exercise for supporters to set a goal to help the participant.* 2. *Get supporter to think about potential barriers to executing the goal and ways to overcome them.* 3. *Supporter develops a goal to engage in participating in psychological health activities.* 4. *Create space for implementation intentions in material.*
	GB3.1	Schedules time to learn strategy from training participant	1. Facilitation	1. SCT	1. Create a specific task for the supporter to schedule time.

*Notes: Change objectives are coded in line with their determinants, with determinants being coded as follows: Knowledge (K), skills (S), beliefs about capabilities and consequences, and identity (BI), goals and behavioural regulation (GB) and social influences (SOC). Each change objective stems from their matrices of change and can be found using the number after the letter it belongs to. When multiple change objectives belong to the same determinant and performance objective, change objectives are separated by alphanumeric symbols. ATRPT = Attribution Theory and Relapse Prevention Theory, CPM = Communication-Persuasion Matric, DIT = Diffusions of Innovation Theory, ELM = Elaboration Likelihood Model, HBM = Health Belief Model, GT = Goal-setting Theory, IPT = Information processing theories, PAPM = Precaution Adoption Process Model, PMT = Protection Motivation Theory, RAA = Reasoned Action Approach, SCT = Social Cognitive Theory, SCOT = Social Comparison Theory, TAIHB = Theories of Automatic, Impulsive and Habitual Behaviour, TGDB = Theories of Goal-Directed Behaviour, TIP = Theories of information processing, TL = Theories of Learning, TPB = Theory of Planned Behaviour, TSD = Theories of Stigma and Discrimination, TSR = Theories of Self-regulation, TTM = Trans-Theoretical Model.*

### Step 4: Design of the Programme

#### Programme Content

The Be Well Plan programme aims to teach participants to start developing their own tailored wellbeing plan. The standard programme is delivered over 5 weeks, allowing participants to develop, implement, and experiment with activities in their plan to suit their specific situation. Delivering sessions over several weeks was hypothesised to lead to larger effects than a short but intensive programme, as research shows that wellbeing programmes are more efficacious when delivered over a longer period of time (Bolier et al., 2013). While this contradicts with for instance brief intensive exposure literature (Öst and Ollendick, 2017) which focuses more on distinct situations rather than the development of more complex behavioural repertoires, it is in line with evidence from related areas such as memory formation and skill acquisition, which usually take time and practice to consolidate (Eichenbaum, 2011). Each session builds on the other, and gradually introduces more complexity. An overview of the each of the five sessions is provided in Table 3. Examples of the programme content described in the table can be found in Supplementary Appendix 1.

**TABLE 3:** Table describing the overall content for the individual sessions of the Be Well Plan.

Pre-programme	Participants are sent an invite to complete a pre-programme measurement that measures outcomes of mental wellbeing, resilience, and distress due to mood problems, anxiety, and stress. This results in an online report, which provides explanation on the findings and points participants to resources they can explore.
Session 1: getting on the same page	• Introduction to facilitators and the group norms. If presented online, particular focus will be placed on explaining the software. • Participants self-reflect on the reasons for participating in the programme and reflect on their personal drivers. Facilitators provide insight into their own drivers to work on their mental health by sharing them with the group. • Participants share their personal drivers with other group members in small groups. • Participants acquire basic knowledge on mental health and definitions for key concepts such as mental health and resilience to create a common language and understanding. • Facilitators delineate scope of the programme: focus on building mental health not treating mental illness. • Participants explore importance of believing in malleability of mental health and the need to have a growth mindset. Evidence on malleability is presented. • Participants are asked to reflect on most surprising thing they learned so far. Participants do a small group sharing exercise where they discuss their choice. • The evidence for different psychological interventions is presented. Participants learn that finding activities that work for their specific situation is key. • Participants are introduced to the fact that over the course of the programme they will practice different ways of making activities work for them. • Participants are introduced to a number of easy mindfulness activities and are asked to choose one to practice during the week based on their own personal preference (the first way to tailor activities). • Participants are asked to set a goal and are introduced to the concept of tiny habits/implementation intentions as a technique to improve the chance of goal-attainment. • *Homework: complete measurement if participants have not completed it before the training*.
Session 2: using your mental health profile	• Participants reflect on their first week of using their plan and how their mindfulness activity worked during the past week. They reflect on whether they need to adjust their plan. Participants share reflections in small groups. • Participants get familiar with the concept of self-compassion (as opposed to self-criticism) and how it can be used to learn from failure and shape our thinking patterns. • Participants practice a self-compassion activity and share their reflections in small groups. • Participants interrogate their measurement result stemming from the integrated measurement. Facilitators can share their own results with the group. • They identify areas they can improve on and select one outcome (wellbeing, resilience, mood, anxiety, and stress) they want to focus on for this session. • Participants are introduced to activity finders: flow charts that map evidence-based activities to each of the activities. • Participants use the activity finders to explore activities they can add to their plan focused on their outcome of choice. Tailoring activities to their outcome of choice is the second way of tailoring that is presented in the programme. • Participants pick one activity from the activity bank to add to their Be Well Plan and will set new goals for the week. • Participants are introduced to the use of prompts and reminders as another method to increase goal attainment. *Homework: complete a survey that allows participants to identify their own values.*
Session 3: your resources and challenges	• Participants reflect on week 2 and make changes to their plan if needed. Participants share reflections in small groups. • Participants work with (and are reminded of) existing resources to their own mental health *via* two practical activities. • The first activity gets participants to choose pictures that display sources of meaning in their life. Participants share the pictures in small groups. Facilitators show their own pictures to start the activity. • The second activity gets participants to identify core values that can be used to guide their life decision and their goals. Participants share which values are important to them. Facilitators share their own values. • Participants then use a custom questionnaire to identify a key resource or challenge they want to work on for the next week. These resources and challenges can be psychological, Interpersonal, health behavioural or external. • Participants are introduced to a new activity finder that maps evidence-based activities to each of the challenges and resources. • Participants explore new activities mapped to the resources and challenges and pick one new activity from the activity bank to add to the Be Well Plan. This is the third way participants are taught to tailor activities. • Participants finish the session with adjusting their Be Well Plan and are reminded of the importance to celebrate small wins related to their mental health (i.e., when they practice activities in line with their Be Well Plan. *Homework: Participants are asked to choose and reach out to a social supporter as part of their weekly activities.*
Session 4: stress, coping, and resilience	• Participants reflect on week 3, adjust their plan if needed and share their reflections in small groups. • The concept of stress and eustress is introduced, and participants learn the effect of stress on our mind and body. • Participants learn about coping strategies (avoidance-focused coping versus more helpful ways, e.g., problem-focused coping). They complete an activity where they reflect on when they used different coping strategies and what impact it had on them. • Participants are then walked through various ways of effective coping using psychological techniques, including identification of cognitive traps and the use of thought defusion. • Participants complete example activities related to cognitive traps and thought defusion in their own life. They share their reflections with other participants in small groups. Facilitators provide examples of their own life. • Participants learn about the importance of asking for help, both from their social support network and professional services. • Participants then choose one new activity specifically focussing on coping using a final activity finder. • They add the new activity to their Be Well Plan. *Homework: participants are asked to complete another measurement, the results of which will be used during the next session.*
Session 5: future-proofing your Be Well Plan	• The participants reflect on the past 4 weeks, what has worked and what has not. Participants share reflections in small groups. • Participants are asked to investigate how their measurement results have changed over the 4 weeks. • The facilitator will introduce the concept of realistic optimism, growth, the fact that progress comes with ups and downs and that it is a slow and gradual process to see change. • They are introduced to Positive Reframing as a technique to deal with setbacks. • Participants will then build their final Be Well Plan, which aims to summarise key learnings from the previous weeks into a standalone plan. • Participants summarise what their best possible mental health looks like. They share their best possible mental health with group members. • Participants highlight their unique drivers and motivations, and existing resources and challenges in their life. They write down the values that are important to them. • Participants set a longer-term mental health goal. • Participants choose the activities they wish to add to their “final” Be Well Plan. They identify their key supporters and reflect on what support services they need in case of emergency.

Each session relies on four key components. Firstly, in each session participants reflect on their personal situation and motivations that are related to their mental health. The participant is asked to perform various self-reflection exercises, for instance reflecting on specific drivers to work on their mental health (session 1), determining which mental health outcomes (e.g., mood, anxiety, and wellbeing) they want to work on (session 2), identifying existing resources and challenges to their wellbeing (session 3), determining which social supporters are present within their life (session 4). In the final session participants reflect on the best version of themselves related to mental health and wellbeing. In other words, the participant is stimulated to create a better understanding of their “self” (Kyrios, 2016) which in turn is used to help determine which psychological activities may be most relevant for them implement within their day-to-day life.

Second, each session introduces participants to *at least* one psychological concept that is considered to be beneficial or “helpful” to building mental health. These include Mindfulness in session 1 (Shapiro et al., 2006), Self-Compassion in session 2 (Neff et al., 2007), Values and Strengths in session 3 (Dahlsgaard et al., 2005), Psychological Flexibility in session 4 (Kashdan and Rottenberg, 2010), and Realistic Optimism in session 5 (Schneider, 2001). The use of these techniques are contrasted with less helpful psychological processes, biases or patterns (e.g., self-compassion versus excessive self-criticism). The aim was to get participants to experience helpful and practical activities related to the psychological concepts, the aim was not to provide a deep dive into each concept. Activities from these specific approaches were chosen as they underpin leading therapeutic models (e.g., CBT, ACT, etc.), are supported by a robust evidence-base, and highlight the malleability of mental health. By teaching these techniques the programme intends to improve participant’s confidence in positively changing their mental health. While the Be Well Plan holds activities from various therapeutic streams other than the ones mentioned above, and aims to stimulate participants to experiment with different activities, highlighting a minimum set of concepts aimed to ensure that participants who were not motivated to experiment would still experience a variety of different activities.

Thirdly participants use the learning from the self-reflection exercises to find different evidence-based psychological activities to implement in their everyday life. The evidence-based activities are collated in an “activity bank,” which were identified by investigating the literature on wellbeing interventions with a systematic review and meta-analysis (van Agteren et al., 2021b). The systematic review identified which intervention types (e.g., CBT, ACT, and positive psychology) were impactful at changing mental wellbeing. The author team then interrogated articles contributing to impactful intervention types and incorporated activities that were included in multiple studies (e.g., though defusion for ACT). Activities were added regardless of the therapeutic or theoretical background, see Supplementary Appendix 1 for a list of the activities. This resulted in a programme that was “theory-agnostic”: activities were chosen based on their demonstrated effectiveness to improve mental wellbeing, rather than their therapeutic background.

Activities can be practiced in two ways, common and personalised. Common activities are matched to specific self-reflection exercises and practised by *each* participant over the course of the five sessions. For instance, after exploring the topic of self-criticism, every participants completes a self-compassion exercise which asks them to “treat yourself as you would treat your friends” (Neff et al., 2007). Personalised activities are activities that are suggested based on individual answers to the self-reflection exercises. These are therefore specific to the participant, meaning that each participant will use a different set of activities in the programme. Over the programme, the participant tries a different way to personalise the activities using so-called activity finders, which are visual aids that link activities to specific topics. In session 1 participants get taught that activities come in different formats, asking participants to try out different formats of mindfulness, e.g., mindful walking, body scan, or deep breathing (Keng et al., 2011). In session 2 participants match activities to a mental health outcome they want to work on, in session 3 they select resources to work on (e.g., self-esteem) and in session 4 they select activities based on a coping style they wish to use. The role of the facilitator throughout the sessions is to model how to use the activity finders, allowing participants to master different ways to tailor activities to their needs.

The fourth principle taught in each session is the basics behind planning and habit formation. At the end of each session, participants are required to choose at least one new activity to practice during the week. Once an activity is selected, participants are guided to refine and personalise the implementation of that activity by developing explicit statements on when and where activities are practiced. The participant first sets a clear goal related to the activity they will practice during the week, which aims to help motivate participants to execute a behaviour. The participant is then asked to form a habit statement, which is derived from the work of Fogg (2019) on **“**Tiny Habits**”** and the concept of implementation intentions (Gollwitzer and Sheeran, 2006). The development of a personalised plan and a focus on implementation means this process is couched in a language of experimentation, where participants try out multiple activities and adjust their plan based on trial-and-error, as they determine which activities work for them, both from a likeability perspective and from their ability to improve the outcome they decided to work on (Proyer et al., 2015). Ultimately this means that each participant will have a different plan consisting of different evidence-based techniques and activities at the end of the programme, matched to their personal situation.

#### Delivery Format and Style

The standard programme was designed to be delivered over five sessions, in-person and online. The programme relies on facilitators using presenter slides, an extensive workbook and supporting video material. The Be Well Plan programme was designed to be deliverable in various formats. First and foremost, it was designed to be delivered as group-based training, where participants interact with one another and share reflections, led by a facilitator. The proposed group size is about 25–30 participants, to balance engagement, feelings of social support, and logistics with cost-effective implementation. The activities were designed to be conducted in pairs and small groups (size ranging between 2 and 5 people) providing flexibility in the way it may be implemented, either in small classrooms or larger settings. Secondly, the programme can be delivered online *via* video conferencing technology (Taylor et al., 2011), which can facilitate all individual components including the sharing exercise, e.g., *via* breakout rooms in conferencing software.

The programme was designed to be delivered without the clear requirement of clinical staff, with the programme utilising a Train-the-Trainer methodology to ensure reach and scalability (Pearce et al., 2012). The facilitators are trained over the course of several week, with a minimum total training time of 26 h. Facilitators in the programme model each of the common activities that are practiced in sessions. This involves facilitators walking participants through the activity, sharing their own experiences of the activity and how they have integrated it into their life. This strategy aims to facilitate a connection between facilitators and participants, in alignment with the importance of the therapeutic relationship in psychological interventions (Clarkson, 2003).

Finally, the programme has an active integration with technology. At the start of the programme participants fill out an online mental health measurement looking at outcomes of mental wellbeing, resilience, and distress, which results in a tailored report. Individual reports serve two purposes: improving the wellbeing literacy (Green et al., 2011) of participants, as well as providing a sense of agency over mental health changes (Schroder et al., 2017). Participants complete the assessment at the start and the end of the programme, allowing them to track their outcomes of interest and “test” whether their personalised strategy has had the desired effect. They also use the results to select an outcome they want to work on during session 2.

## Discussion

This article outlines the use of an intervention development framework to guide the design and development of a mental health intervention. Significant detail about the development process and the intervention itself is provided to allow transparency for end-users, researchers, practitioners and policy makers who may wish to access, evaluate or replicate the programme. At the same time, it serves to illustrate a methodology that allows for improving the reporting of development and design processes for psychological interventions. Firstly, we will discuss the Be Well Plan in the context of other existing mental health interventions. Secondly, we will discuss the implications of using this reporting approach which provides an extensive descriptions of intervention methodology and design, and compare its strengths and limitations to other approaches.

Although a plethora of mental health interventions exist, the needs analysis that underpins the Be Well Plan led it to be designed differently to most other interventions in various ways, e.g., the need for a focus beyond mental illness, the need to personalise, and the need to focus on behaviour change. Firstly, most existing mental health interventions are focused on treating mental *illness* (Das et al., 2016) and not necessarily building or promoting mental *health* (Keyes, 2007). Simply relying on techniques designed to treat symptoms of illness could be limiting for a mental health promotion intervention that aimed to be suitable for clinical and non-clinical populations, considering the existence of differential antecedents for mental illness and wellbeing (Kinderman et al., 2015). For example, simply extrapolating techniques that were developed to address maladaptive thought patterns might only be relevant for a proportion of participants, whose maladaptive thoughts patterns are the cause of their challenges, rather than for instance a lack of purpose or positive social relations. Similarly, traditional “wellbeing” interventions such as positive psychology interventions are typically designed to target positive constructs, and do not necessarily address the potential maladaptive antecedents of poor mental health (Seligman et al., 2005). A notable exception can be found in ACT-based interventions as they address both states (Fledderus et al., 2012), although they are still typically applied in the context of mental *illness* rather than promotion of *wellbeing* (Doorley et al., 2020).

The Be Well Plan is “theory agnostic” and explicitly deviates from existing interventions that are underpinned by a set therapeutic paradigms. A broad variety of interventions based on CBT, ACT or positive psychology exist (Hofmann et al., 2012; Öst, 2014; Carr et al., 2020). While they have demonstrated, on average, significant impacts on mental health outcomes, there is no decisive evidence to suggest that these are the only valid approaches to improving mental health, particularly when the focus is on mental health promotion and not simply the treatment of mental illness (Slade, 2009). Rather, the Be Well Plan includes a set of empirically derived psychological activities from across paradigms targetting various antecedent, with which the participant experiments with, drawing a parallel with process-based therapies (Hofmann and Hayes, 2019). Future studies that focus on outcome evaluation are planned to validate whether this approach will lead to cause the hypothesised positive impact on mental health outcomes.

Furthermore, a key aim for the programme is to create lasting behavioural change for participants, using guidance from behaviour change taxonomies (Kok et al., 2016). Instead of providing participants with activities and leaving it up to participants to decide which activities can be used as part of their life journey to good mental health, the programme encourages participants to match and experiment with activities to their needs, which may be driven by distress or illness, by wellbeing needs or by both. This approach is in line with a personal recovery approach to mental health promotion, which is captured by Anthony (1993) as “a deeply personal, unique process of changing one’s attitudes, values, feelings, goals, skills, and/or roles. It is a way of living a satisfying, hopeful and contributing life, even within the limitations caused by illness.” The focus of the intervention is to guide participants to develop a sustainable wellbeing plan and provide them with tools to monitor their mental health over the life-course. This required the integration with an online assessment that facilitated real-time reporting. While tracking of change as a result of interventions is common in e-health solutions, particularly those focussing on Ecological Momentary Assessment (Shiffman et al., 2008), the integration of reporting capability in group-based interventions is uncommon. It follows the growth in popularity of outcome monitoring (Carlier et al., 2012; Boswell et al., 2015), where health practitioners are able to monitor treatment progress, and expands this by providing this same real-time capability to participants; a principle which is not typically seen in group-based programmes. This fundamentally aims to provide self-agency and gives the participant ownership over and accountability on their own mental health, now and in the future (Clarke et al., 2014).

Detailed outcome evaluation will be needed to determine the impact of the approach chosen in the Be Well Plan. Two studies have, at time of writing this manuscript, been completed, with further studies underway. The first completed study was an uncontrolled intervention study aimed at determining the initial impact of the intervention, finding significant improvements in outcomes of wellbeing, resilience, and psychological distress, most notably for those with more problematic mental health scores at baseline (van Agteren et al., 2021a). Preliminary findings of a randomised controlled study are replicating the positive findings of the first study, with a manuscript currently being prepared. The Be Well Plan evaluation is ongoing, with future studies focussing on investigating who benefits most from the intervention and investigating the impact of different formats of the Be Well Plan (e.g., face-to-face versus online) as well as its longer term impact, including comparing its impact to other psychological intervention types.

### Improving the Reporting Standards for Mental Health Intervention Research

The article aimed to provide a foundation for anyone who seeks more detailed information about the Be Well Plan’s scientific foundations. Using an extensive intervention development process such as IM to document intervention design allows for detailed replication of the theoretical approach to the programme. By doing so, IM provides a specific methodology to improve attempts at reproducibility and replicability, following other positive developments in reporting standards for interventions and research. One example of such a development is the more frequent use of checklists such as the TIDieR checklist (Hoffmann et al., 2014). While TIDieR asks detailed questions regarding theoretical underpinnings, materials, procedures, tailoring, and iterations, it lacks a focus on describing the individual detailed components of the intervention such as the one reported in Table 2. Merely requesting researchers to explain that their intervention was based on for instance CBT-based principles or the Theory of Planned Behaviour does not provide sufficient details about the exact design principle of intervention components. A more detailed approach, *via* the use of taxonomies and ontologies to break down intervention components into active building blocks, is becoming more frequent (Larsen et al., 2017). The development of matrices of change, use of BCTs and guidance from the TDF provides an in-depth explanation of each component of the intervention, which can provide an extra safeguard at achieving intervention impact.

The use of IM or similar approaches such as the Behaviour Change Wheel (Michie et al., 2011b) also protects against a limitation of reporting checklists. These checklists are often used *after* the intervention has been designed, even if they were supposed to be used to guide design and studies. By using intervention development frameworks, the exact steps of the *development* process are captured throughout the entire project (Eldredge et al., 2016). This extensive process does come with its own limitations (Peters et al., 2015), including their requirement of resources. This ultimately also influenced the way the Be Well Plan was developed as it mainly on a small “core” group of contributors (JA, MI, GF, and KA) who guided the large majority of the development work, while the larger multi-disciplinary group provided input at half a dozen meetings and at key touch points. This was mainly the result of practical constraints (e.g., availability to contribute in-kind on top of existing workloads) and on lack of familiarity with the process, which poses a general limitation to methods such as IM (Eldredge et al., 2016). If programmes require rapid development with limited resources, using the current framework might at first glance not be favoured over a more pragmatic approach. The ultimate effectiveness of programmes that are designed, developed and implemented within short periods of time without adequate methodological considerations, may however be suboptimal in their ability to change outcomes and limit our ability to advance psychological science and improve mental health research. Adoption of rigorous development methodologies and investing resources in their use, such as is demonstrated in the case of the Be Well Plan, may be a way to counter this, pushing us another right step in the direction of scientific rigour in psychological intervention research (Prager et al., 2019).

## Data Availability Statement

The original contributions presented in the study are included in the article/Supplementary Material, further inquiries can be directed to the corresponding author/s.

## Ethics Statement

The studies involving human participants were reviewed and approved by underpinning data stems from a variety of studies approved by the Flinders University Social and Behavioural Research Ethics Committee, project numbers (PN) 7834, 7891, 7350, 7358, 7221, 7218, and 8579. The patients/participants provided their written informed consent to participate in this study.

## Author Contributions

JA and MI: project methodology, needs analysis, programme objectives, theoretical framework for programme, programme material development, and manuscript write-up. KA: project methodology, programme objectives, theoretical framework for programme, programme material development, and manuscript write-up. DF: programme objectives, theoretical framework for programme, and manuscript write-up. GF, LW, and AH: programme objectives, theoretical framework for programme, programme material development, and manuscript write-up. MK: project methodology, guidance of process and clinical input, and manuscript write-up. All authors contributed to the article and approved the submitted version.

## Conflict of Interest

The South Australian Health and Medical Research Institute which employs JA and MI, receives financial compensation from providing the Be Well Plan to organisations and the community. The remaining authors declare that the research was conducted in the absence of any commercial or financial relationships that could be construed as a potential conflict of interest.

## Publisher’s Note

All claims expressed in this article are solely those of the authors and do not necessarily represent those of their affiliated organizations, or those of the publisher, the editors and the reviewers. Any product that may be evaluated in this article, or claim that may be made by its manufacturer, is not guaranteed or endorsed by the publisher.
