# Supplementary material for: Using the Intervention Mapping Approach to Develop a Mental Health Intervention: A Case Study on Improving the Reporting Standards for Developing Psychological Interventions
Source: Front Psychol. 2021 Oct 5;12:648678. doi: 10.3389/fpsyg.2021.648678 (PMC8524131; doi:10.3389/fpsyg.2021.648678)
Supplement: Supplementary file 1 [file Data_Sheet_1.docx]

**Supplementary appendix to**

**“Improving the reporting standards for developing psychological interventions: utilising the Intervention Mapping approach to design a mental health intervention.”**

**Authors: Joep van Agteren (JA), Matthew Iasiello (MI), Kathina Ali (KA), Daniel Fassnacht (DF), Gareth Furber (GF), Lydia Woodyatt (LW), Alexis Howard (AH), Michael Kyrios (MK).**

Table of Contents

[Section 1. Development process 3](#_Toc78964613)

[Section 2. Needs Analysis Supplement (step 1) 4](#_Toc78964614)

[Determinants for change choice justification 4](#_Toc78964615)

[Collated feedback from previous training programs 7](#_Toc78964616)

[Section 3. Matrices of Change (step 2). 13](#_Toc78964617)

[Section 4. Be Well Plan program examples (step 4) 21](#_Toc78964618)

[Short overview of the five sessions in the Be Well Plan 21](#_Toc78964619)

[Examples to core components to individual Be Well Plan sessions 22](#_Toc78964620)

[Index of activities 23](#_Toc78964621)

[Examples of self-reflection activities 25](#_Toc78964622)

[Examples of sharing exercises between participants 29](#_Toc78964623)

[Examples to highlight core psychological principles participants develop 30](#_Toc78964624)

[Examples of the evidence-based psychological activities 32](#_Toc78964625)

[Planning fundamentals and behaviour change 38](#_Toc78964626)

[Example of the measurement report 40](#_Toc78964627)

[References for appendix 44](#_Toc78964628)

# Section 1. Development process

**1. Project governance**

Project development responsibilities were split into three levels. The initials below refer to the main authors of the manuscript.

- Level 1: the project leads. The main responsibility for guiding the project was placed with JA and MI, who together coordinated the development of the training and its implementation. They were overall responsible for the needs analysis, creation of the majority of program materials and the scientific rational.
- Level 2: the project group. The project leads worked closely together with the project group (DF, LW, KA, MK, GF, AH) who supported the project leads in development of material and the validation of the design process for all the Intervention Mapping steps.
- Level 3: the stakeholder groups. The training was developed by involving two stakeholder groups. The first stakeholder group consisted of professional staff working for the South Australian Health and Medical Research Institute (SAHMRI) Wellbeing and Resilience Centre (WRC), who deliver mental health training and provide wellbeing assessment to community members. They brought expertise in delivery and implementation of mental health training in various population cohorts (older adults, disadvantaged youth, workforces etc) and played a crucial role in ensuring that the new program can be implemented and scaled outside of a research setting. The second stakeholder group consisted of a multi-disciplinary team of academics and professionals working at Flinders University (Adelaide, Australia) who specialised in psychology, social work, education, group-work, student services, wellbeing research and physical health behaviours. These groups were actively used to underpin step 1-3 via serious of meetings to help shape the steps and validate them once the project group finalised the content to each step.

**2. Design process**

Firstly, the project leads verified the need to create a new training program in 2018 after conducting formative research and established the requirement to use Intervention Mapping (1) to guide the project. The formative research involved a preliminary needs analysis and determined the need to conduct a systematic review to determine effective intervention components, see main manuscript for the needs analysis findings. The project leads went on to engage the first stakeholder group to test the initial draft needs analysis and program objectives (IM steps 1 and 2). After verifying that the focus was in line with expectations, the project leads worked on finalising the needs analysis, and drafted the initial program objectives and draft matrices of change.

Parallel to this process, the project group and the second stakeholder groups were engaged in 5 general meetings and various sub-meetings to provide input into and validate the results of IM step 1 to 2. The second stakeholder group was furthermore involved in validating step 3, ultimately resulting in a theoretical backbone to the training. The project leads used the input from these meetings to further develop the theoretical intervention components and the draft content into a preliminary program. First a program delivery framework was created, outlining the proposed sessions (the Be Well Plan was designed to take place over multiple weeks, which we refer to as sessions), the underpinning rationale for each of the sessions and the delivery format for the training. This theoretical framework for the Be Well Plan was evaluated and approved by both stakeholder groups. The design process which solidified the theoretical rationale took a total of 10 months.

The project group, led by the project leads, went on to develop a written version of the program in the form of a program booklet. This had the purpose of creating the narrative for the program and allowed for the booklet to be used as an underpinning resource, e.g. to be used in case participants would miss a session. The program booklet was subsequently used to underpin the session structure for the interactive training and its materials, most notably the workbook, the presentation slides and the supporting trainer notes. The Be Well Plan was aimed to be delivered using multiple formats including in-person delivery and online facilitated delivery (over platforms such as Zoom (2)). The team set out to first test and iterate the in-person delivery.

**4 testing and iteration**

The subgroup completed a first draft of each session and the accompanying materials and performed two small-scale pilot tests: one in university students (n=30) and one in colleagues of the project team members (n=7). During these pilot tests several improvement areas were noted, including the need for simplification of materials and the creation of program materials that was suitable for vision impaired participants; the colour scheme and various examples caused issues for a participant with severe vision impairment. The project team met on various occasions to improve the training materials in response to the pilot over the course of January to February 2020 and involved a professional designer in the final program material designs.

After initial testing of the in-person version, further in-person testing was placed on hold as a result of the COVID-19 pandemic, which constrained in-person delivery within Adelaide (Australia), as restrictions on gatherings came into place in March 2020, see van Agteren et al. (3) for a brief description on the impact of COVID-19 in South Australia. As a result, testing of online delivery of the training was fast-tracked. The training was re-formatted to fit the online setting (e.g., certain activities would not work on online formats) and the training was recorded in full as a contingency for when online facilitated delivery would not be feasible. This had the added benefit that participants who missed a session could continue in the program without missing important educational content. The online delivery was predominantly performed via the online conference software Zoom (version 5.3). Zoom was chosen as it allowed the training to stay true to some of its core principles including the need to share in small groups (via the breakout room function), the ability to access Zoom without an account and the visual lay-out working well for groups of maximum 30 participants.

# Section 2. Needs Analysis Supplement (step 1)

### Determinants for change choice justification

The below table describes the justification for choice of behaviour change determinants underpinning the logic model for change. The table lists the determinants as described in the Theoretical Domains Framework (TDF) (4) and provides a definition for the determinant. It then provides a judgement of the relative perceived importance in relation to the Be Well Plan and the judged changeability of the determinant in the context of the training. It then provides a simple judgement of inclusion and a brief written rationale to underpin the judgement.

| **Determinants** | **Definition in IM** | **Importance** | **Changeability** | **Include** | **Why** |
| --- | --- | --- | --- | --- | --- |
| Knowledge | An awareness of the existence of something | ++ | +++ | YES | Having an understanding of the importance of mental health and wellbeing, how you can influence it and how this intervention can help improve it is. It is a pre-requisite for the intervention components to work and to build motivation for change. |
| Skills | An ability or proficiency acquired through practice | ++ | ++ | YES | Competence in dealing with situations that can threaten mental health and wellbeing is necessary by practicing skills or activities. Mastery of skills is essential to the program. |
| Social/professional role and identity | A coherent set of behaviours and displayed personal qualities of an individual in a social or work setting | + | + | NO | The main focus is to design an intervention that works regardless of social identity or social roles. It is not the main focus of the intervention, but may be considered in the implementation phase for project delivery. |
| Beliefs about capability | Acceptance of the truth, reality or validity about an ability, talent or facility that a person can put to constructive use | +++ | ++ | YES | Empowerment and sense of agency are desired states to ensure participants feel adequate in being able to deal with stressors and adversity when it arises. Increasing self-efficacy is an important precondition to learn behaviours that are associated with a stigmatised outcome (such as mental health outcomes). |
| Optimism | The confidence that things will happen for the best or that desired goals will be attained | + | + | No | The intervention will rely on beliefs about capabilities and consequences, and goal setting to ensure that end-states are achieved. Specifically targeting optimism is out of scope. The topic of realistic optimism is however touched upon when investigating participant’s changes in mental health levels. |
| Beliefs about consequences | Acceptance of the truth, reality, or validity about outcomes of a behaviour in a given situation | +++ | ++ | YES | The intervention will place emphasis on explaining how and why techniques used in the interventions are beneficial for health and wellbeing, thereby positively influencing beliefs and outcome expectancies. The belief that mental health is changeable is key to program impact. |
| Reinforcement | Increasing the probability of a response by arranging a dependent relationship, or contingency, between the response and a given stimulus | + | +++ | NO | Reinforcement is not the aim of the intervention content, but will be included in the design of the different 'vehicles’ of the intervention in step 4, e.g. mobile applications. |
| Intentions | A conscious decision to perform a behaviour or a resolve to act in a certain way | + | ++ | NO | The intervention will aim to help the individual set a strategy and give them the ability to perform all steps in the strategy. The main goal of the intervention is not to act in a specific way, but rather to reach a goal and adjust the behaviours until that goal is achieved. |
| Goals | Mental representations of outcomes or end states that an individual wants to achieve | +++ | +++ | YES | In order to develop an overarching mental health strategy, the participant is expected to set gradient goals to help build the overarching outcomes. Will also include implementation intentions and action planning, |
| Memory, Attention and decision processes | The ability to retain information, focus selectively on aspects of the environment and choose between two or more alternatives | + | + | NO | No explicit focus is placed on memory and attention processes in the content or the underpinning construct. The construct will be taken into account when thinking of delivery format. |
| Environmental context and resources | Any circumstance of a person’s situation or environment that discourages or encourages the development of skills and abilities, independence, social competence and adaptive behaviour | ++ | + | NO | The environment is powerful in changing someone's behaviours. The aim of the intervention is to design a universal solution regardless of the presence of environmental blocks. When the intervention gets implemented within specific environments, this determinant can be addressed, but other than the impact of social relationships, will be a focus for the implementation stage. |
| Social influences | Those interpersonal processes that can cause individuals to change their thoughts, feelings, or behaviours | ++ | ++ | YES | Peer relationships are closely related to our mental health, but are often ignored in interventions. The existing intervention will focus on bringing in loved ones, to ensure a supportive environment where possible |
| Emotion | A complex reaction pattern, involving experiential, behavioural, and physiological elements, by which the individual attempts to deal with a personally significant matter or event | + | ++ | No | While emotion is an outcome of the study, it is not specifically a direct determinant of action. Where possible, affective states will be improved in order to ensure optimal engagement with the intervention. |
| Behavioural regulation | Anything aimed at managing or changing objectively observed or measured actions | ++ | ++ | Yes | Application of behavioural regulation, which includes self-monitoring is relevant for physical activity behaviours underpinning the health component. |

### Collated feedback from previous training programs

Feedback recorded below summarises participant responses to the question what they liked about previous mental health group training and what they did not like. A number of academic papers have been written on the outcomes of the training that these participants took part in (5-8). The first column provides a code to identify different respondents. Each response is given a general topic and the implications for the Be Well plan are indicated. In the final column the actual anonymised response can be found. Any identifiers are redacted.

| Responder ID | Overall topic | Implications for Be Well Plan | Quotes |
| --- | --- | --- | --- |
| WRC54 | Facilitation | - Ensure trainer professionalism - incorporate sharing of personal reflections | [Trainers] worked very well together and transitioned from topic to topic very well. Both were passionate about the subject area. Both also prepared to be vulnerable and share personal experience relating to the topic. |
| WRC55 | Facilitation | - Ensure trainer professionalism - incorporate sharing of personal reflections | [Trainers were] experienced, engaging, funny, with relevant stories |
| WRC2 | Facilitation | - Ensure trainer professionalism - Build safe environment for sharing | Trainers had many effective skills but could be more effective with some tweaking and coaching on their presentation strengths and areas for improvement. Very engaging and passionate and made the audience feel comfortable. |
| WRC1 | Facilitation | - Ensure trainer professionalism - Build safe environment for sharing | They were both great facilitators sharing genuinely good examples on each of the skills. They never made anyone in our group feel uncomfortable about sharing if they didn’t wish to, very respectful. The training was also done at a nice pace " |
| WRC56 | Facilitation | - incorporate sharing of personal reflections | The personal experiences were very poignant, and added a lot of value - whether the story was big or small. |
| WRC3 | Facilitation | - Trainer qualities - Build safe environment for sharing | [Trainers had] Complementary styles, established an atmosphere of comfort/trust, genuine, fun, human!! |
| WRC6 | Facilitation | - Build safe environment for sharing | [The Trainers] Created a great environment where it was reasonably easy to interact with others. |
| WRC4 | Facilitation | - incorporate sharing of personal reflections | I appreciate the sharing of trainers personal stories as it made me realize ‘it’s not just me! |
| WRC5 | Facilitation | - incorporate sharing of personal reflections | <3 Very approachable. Provided real life examples based on their own experiences. Made the material more tangible |
| WRC58 | Facilitation | - Select diverse trainers where possible | I thought their diversity of style and delivery was engaging and interesting |
| WRC14 | Format | - Create clear and simple resources | A spare booklet for note taking, the book isn’t in order and note taking pages were limited. |
| WRC2 | Format | - Ensure adequate session length and program timing | Some sessions … were too short for exercises. More time speaking with strength group on all top 5 so I guess we need [more days] |
| WRC15 | Format | - Create clear and simple resources | A copy of slides or alternate notes Ways to consolidate training to become ‘hard wired’ |
| WRC16 | Format | - Ensure adequate session length and program timing | Option for a course to be spread out over a longer period of time. More relatable examples used by some trainers. More take home materials or references |
| WRC18 | Format | - Ensure that the sessions are engaging and offer a mix of content | mix up delivery methods as there is a bit of death by Powerpoint happening. You may find value in Dr. John Medina's work and Garr Reynolds Presentation Zen and Harvard's Making Thinking Visible. Consider drawing more upon the wisdom of participants even while teaching the concepts, not just in debrief. |
| WRC19 | Format | - Create clear and simple resources | [Provide] Some take away que cards. Reminders of what we learned |
| WRC20 | Format | - Ensure adequate session length and program timing - Ensure that the sessions are engaging and offer a mix of content | [Provide] More practical examples. [Provide] More time to practice the exercises |
| WRC25 | Format | - Ensure adequate session length and program timing - Ensure that the sessions are engaging and offer a mix of content - Build safe environment for sharing | Less reflection, more funny videos to represent concepts with group discussion. Talking one on one with people we don’t know about topics is confronting and at times uncomfortable. Course is very long, could be condensed |
| WRC26 | Format | - Allow sharing of personally relevant experiences | I wasn’t particularly interested with sharing personal examples in a group setting and it felt trivial discussing [smaller adversities] |
| WRC30 | Format | - Ensure adequate session length and program timing | I had a bit of afternoon fatigue but not sure how to avoid that. |
| WRC33 | Format | - Ensure that the sessions are engaging and offer a mix of content | More time up and out of our seats doing activities with different people. I know not everyone likes being active in trainings but I think it could have added another element for different learning styles. |
| WRC34 | Format | - Build in sufficient opportunity to ask clarifying questions | Expand more on areas of ambiguity, like what was done when difficult questions were asked (very well handled btw) |
| WRC37 | Format | - Provide opportunities for follow-up after the program - Streamline registration process | Some type of a follow up program to track how people put it in practice. Registration / initial tests were somewhat messy. Two different websites, messages from different people etc |
| WRC36 | Format | - Clearly indicate scope of the program - Provide clear background to role of biopsychosocial factors | I strongly disagreed with the idea that wellbeing begins with the individual, I don't believe this is an evidence based concept and in my work as a social worker what I have learnt is that relationships and communities and societies have an enormous impact on our wellbeing, so I think it's important to acknowledge this and to acknowledge the things we can and cannot control, to avoid the danger of being overly individualistic. We have to be very careful not to tell people that they are responsible for their own adversity or failures. It is not possible to CBT yourself out of structural disadvantage or complex trauma. So, acknowledging limitations of this course is important I think. I also found the slide at the beginning about mental disorder being at the opposite end of flourishing to be slightly misleading and problematic as someone with a diagnosis can also be flourishing at the same time! |
| WRC40 | Format | - Ensure that the sessions are engaging and offer a mix of content - Provide opportunities for follow-up after the program | More interaction with other participants. More activities for experiential learning. Definitely requires follow up program. |
| WRC42 | Format | - Build safe environment for sharing | Advance notice that multiple scenarios required Adversities "Interpersonal problems" …. forewarned is forearmed |
| WRC54 | Format | - Ensure that the sessions are engaging and offer a mix of content - Ensure adequate session length and program timing | 1. Move the groups around a bit more during the day to encourage people to work with different people and get even more perspectives on issues. 2. Option for a walking lunch - walk and talk around the block. take a theme and share it with a partner. new perspective outside the building 3. Sometimes the slides moved very quickly - hard to take notes. Would be great to have a copy of these. |
| WRC55 | Format | - Ensure that the sessions are engaging and offer a mix of content - Ensure adequate session length and program timing | Materials could be more comprehensive. Slides have lots of info and there isn’t enough time to write them all down. More moving around and working with other people than predominantly with the same people. |
| WRC13 | Format | - Build safe environment for sharing | I found it awkward when people started raising big picture issues or problems in their lives. This course tried to just deal with the small or medium issues but when people raise significant issues from their lives the conversation gets heavy and it feels like the presenters are not sure where to go with it. Hard balance to keep the conversation light but still meaningful and useful. |
| WRC23 | Format | - Clearly indicate scope of the program | I think the course was very good as an introduction, I was anticipating something more in-depth and challenging, it did not offer me the depth I was looking for, but I take responsibility for not considering it more carefully. |
| WRC47 | Format | - Ensure adequate session length and program timing - Provide opportunities for follow-up after the program | There was a lot of content covered that it could almost be made into a longer course to allow time to fully delve into the skills. The course can be a bit emotionally challenging but not much you can do about that…There could be a shorter follow-up course to allow further integration of skills and discussion. |
| WRC49 | Format | - Clearly indicate scope of the program - Aim for a balance between depth and reach | Would have enjoyed more time and some greater depth - but wouldn't fit into this course in the format, will try to read further myself or do you run other courses with longer / more details / more exploration of these ideas? I missed some people's comments - could not hear without microphone I would prefer examples given to use in ETR, ACR |
| WRC50 | Format | - Aim for a balance between depth and reach - Ensure that the sessions are engaging and offer a mix of content | I really [the] training and I have a lot to apply. For me (personally) I believe the content could have been covered over one day with smarter breaks. I also found that the workbook relies on note taking of most topics …. I found the exercises in the workbook to be repetitive and I felt certain participants in my group didn't take them as seriously as two days went on and may have got a little bored of this style of repeat exercises. |
| WRC51 | Format | - Ensure adequate session length and program timing - Aim for a balance between depth and reach | Not really enough time for group discussions to talk between one another. I felt that I already practice a lot of these skills so would be interested in learning more about turning around conversations from people who are not resilient. The course focused very much on improving my own wellbeing and less on that which surprised me. |
| WRC52 | Format | - Ensure adequate session length and program timing | Really enjoyed the course, thank you, but sometimes felt the discussion sessions were a bit rushed. |
| WRC53 | Format | - Ensure adequate session length and program timing | At times a little more time to discuss with partner to understand limits. Good materials of own reflections to keep as ongoing reference material. |
| WRC60 | Format | - Ensure that the sessions are engaging and offer a mix of content - Clearly indicate scope of the program | I think there could have been more group activities which involved moving into different groups and interacting with other staff members. There was a lot of "reading from the powerpoint" The presenters were very enthusiastic. Some aspects of the program could cause tress to people who have been through traumatic events in their lives. Some traumas do not lead the person involved to "find a positive" and can make them feel guilty that they are not coping as well as they "should" |
| WRC61 | Format | - Create clear and simple resources - Ensure adequate session length and program timing | The booklet would be most helpful if it followed the sequence of the training. Understand pace to fit all content but not really enough time to really think through and record goals/plans activities. |
| WRC66 | Format | - Ensure adequate session length and program timing | like any [Professional Development] day I find myself falling off past 2pm. |
| WRC68 | Format | - Ensure adequate session length and program timing | Just wondering if it would be better to split the [training] up as I found the [format] a bit tiring. |
| WRC69 | Format | - Provide opportunities for follow-up after the program & embedding during the program | This training program is really useful for an individual. But noticed some people do not implement in their life and behaviour. |
| WRC44 | Format | - Provide opportunities for follow-up after the program & embedding during the program | [Provide] Opportunity to practice over time. Split training |
| WRC28 | Format | - Provide opportunities for follow-up after the program & embedding during the program | I enjoyed it. Two full days. Might be better one day a week for two weeks. To avoid the overload feeling and allow time to process the information before being given more information |
| WRC1 | Improvement points | - Create clear and simple resources - Ensure a follow-up process | More space to write in our booklets (I didn’t bring any note paper) … Not sure if it’s coming - but maybe an email reminder down the track to remind us of the skills so we can try continuing regularly using them |
| WRC7 | Improvement points | - Ensure a follow-up process | Implement a follow up session / webinar to check in and share our journey. |
| WRC8 | Improvement points | - provide examples to improve reflection | [Provide] some scenarios for people within the booklet who struggle to come up with their own situations ... That’s all I can think of! |
| WRC17 | Trainers | - provide examples to improve reflection | The trainers were great at interacting and connecting with the audience (me), the examples that they provided in every … topic was reflective which help[s] strengthen the understanding of each skill topic. |
| WRC38 | Trainers | - Use easy-to-understand language | Delivery, facilitators were amazing. Also, the consistent and simple to understand language that is used. |
| WRC39 | Trainers | - Use easy-to-understand language | Facilitators were passionate but also didn’t over talk. Delivery kept it interesting and easy to keep attention. |
| WRC62 | Training | - Provide a safe space for participants - Ensure group norms are clear up front | As someone with a childhood (teenage years) trauma (sexual assault) I found [an example] too confronting so I deeply appreciated how you provide space @ "permission " for people to leave the room from the start. To be honest, if I hadn't been seeing a psychologist every 2 weeks or so for the past 18 months, most of this would have been too much for me to process. |
| WRC14 | Training strengths | - Ensure that the sessions are engaging and offer a mix of content | Beneficial for both personal and professional use. A relaxed and open minded setting where everyone feels comfortable to speak their voice. Break times with yoga stretches were awesome! |
| WRC7 | Training strengths | - Allow reflection, practice and sharing - Highlight evidence-base | Based on research, practical application with lots of opportunities to practice. |
| WRC8 | Training strengths | - Allow reflection, practice and sharing - Highlight evidence-base | That they had examples and visuals to support what they were saying. The course was evidence based and provided opportunities to take down names of studies to continue to read at a later date. |
| WRC3 | Training strengths | - Provide a safe space for participants | Atmosphere of trust & enjoyment, meeting new people & hearing of different experiences & perspectives. Applying all skills to real life. Encouragement gained from course. |
| WRC6 | Training strengths | - Ensure that the sessions are engaging and offer a mix of content | As I don't like role-play, I found the group work/discussions etc to be a less confronting experience |
| WRC10 | Training strengths | - Aim for a balance between depth and reach | Relatable to all walks of life. Well-rounded course with each section linking well to the next. |
| WRC21 | Training strengths | - Ensure that the sessions are engaging and offer a mix of content | A great balance of theory and individual reflection/ practice |
| WRC24 | Training strengths | - Provide a safe space for participants | Calm and peaceful trainers/atmosphere, honesty, applicable material personally and professionally |
| WRC27 | training strengths | - Ensure that the sessions are engaging and offer a mix of content - Provide clear and simple resources | Great organisation and team-work. Great balance of information and activities. Really appreciate folder with booklet and gratitude book. Beautiful delicious food. |
| WRC35 | training strengths | - Ensure that the sessions are engaging and offer a mix of content | Good mix of slides, videos, facts, quotes, examples and group work. Enabled content to be reinforced. |
| WRC41 | Training strengths | - Balance group size | Interactions, group work. Groups were large enough for interactions but small enough for inclusion. |

# Section 3. Matrices of Change (step 2).

The below section provides the matrices of change for the Be Well Plan, looking at the behavioural and environmental outcomes.

| Table S1  Matrices of change for behavioural outcome 1 showing the change objectives of each determinant and performance objective (PO). | | | | | | |
| --- | --- | --- | --- | --- | --- | --- |
| **Behavioural outcome 1: Engages in regular activities that are known to increase the mental health and wellbeing of the individual** | | | | | | |
| **Code** | **Performance Objective** | Determinants | | | | |
|  |  | Knowledge | Skills | Beliefs in capabilities & consequences | Goals and Behavioural regulation | Social influence |
| **PO 1.1** | **Creates understanding of mental health, and its relationship to mental illness and wellbeing** | **K1.1a** Defines mental health, wellbeing and mental illness **K1.1b** Lists behavioural and non-behavioural factors that are associated with psychological health outcomes  **K1.1c** List positive outcomes associated with good psychological health | **S1.1** Demonstrates ability to process entry-level information on psychological health | **BI1.1** Expresses positive attitude towards learning about psychological health |  |  |
| **PO 1.2** | **Understands that good psychological health can actively be achieved via different intervention types, regardless of physical or mental illness** | **K1.2a** Lists evidence-based interventions to build psychological health  **K1.2b** Explains the way intervention types can influence psychological health  **K1.2c** Explains current evidence-status for individual intervention types on improving psychological health | **S1.2** Demonstrates capacity to determine whether or not interventions are based on evidence | **B1.2a** Expresses positive attitude towards learning about different interventions to build psychological health  **B1.2b** Judges that psychological health is malleable via different interventions  **B1.2c** Beliefs in one’s own ability to judge evidence on interventions and to relate back to own experience |  |  |
| **PO 1.3** | **Understand that personal characteristics influence which interventions participant should be considering to improve mental health** | **K1.3a** Explains that personal characteristics and context has an impact on personal impact of interventions  **K1.3b** Understands that personal characteristics and contexts for an individual may change, which can influence the impact of interventions | **S1.3** Demonstrates capacity to reflect on personal characteristics that apply to the individual | **BI1.3** Expresses positive attitude towards interrogating personal characteristics |  |  |
| **PO 1.4** | **Understands that good psychological health requires a life-course approach** | **K1.4a**  recalls evidence that discusses fluctuations in psychological health outcomes throughout life  **K1.4b** understands that psychological health over the life-course requires a committed approach |  |  | **GB1.4** Develops commitment to actively construe a strategy to build psychological health |  |
| **PO 1.5** | **Is aware of personal psychological health profile (wellbeing, resilience and psychological distress)** | **K1.5a** Understands how to access measurement tools in the program to understand individual mental health  **K1.5b** understands that psychological health consists of different personal outcomes | **S1.5a** Demonstrates ability to complete psychological health assessment methods  **S1.5b** Demonstrates ability to interpret scores on psychological health assessment methods | **B1.5a** Expresses positive attitude towards validity of psychological health assessment methods  **B1.5b** Expresses positive attitude towards measuring their psychological health profile over time |  |  |
| **PO 1.6** | **Creates overview of resources and challenges for their psychological health** | **K1.6a** Lists common resources and challenges for good psychological health  **K1.6b** Identifies personal and contextual resources for their psychological health  **K1.6c** Identifies personal and contextual barriers to their psychological health  **K1.6d** Describes importance of social relationships | **S1.6** Demonstrates ability to self-reflect on personal and contextual resources and challenges | **BI1.6** Has positive attitude towards expanding social support to build psychological health | **GB1.6** Monitors personal resources and challenges to psychological health over time | **SOC1.6a** Investigates social support for implementation of psychological health strategy  **SOC1.6b** Determines influence of social identity to form psychological health strategy |
| **PO 1.6.1** | **Determines which resources and challenges are currently present, which can be improved on using the program and which ones are out of scope** | **K1.6.1a** Recalls scope of the program  **K1.6.1b** Describes types of challenges that cannot be altered by a psychological skills training  **K1.6.1c** Understands where to find help for psychological issues out of scope of the program | **S1.6.1** Demonstrates ability to determine which resources and challenges can be managed or improved by themselves | **BI1.6.1** Demonstrates positive attitude towards reaching out or using other mental health and wellbeing services |  |  |
| **PO 1.6.2** | **Determines when other programs or services for mental health and mental wellbeing need to be considered** |  | **S1.6.2** Demonstrates ability to compare personal outcomes to target areas for the training |  | **GB1.6.2** Set goal to seek help for any psychological or social issues outside of the scope of the program |  |
| **PO 1.7** | **Determines personal motivators for wanting to engage in activities that promote psychological health** | **K1.7a** Names list of motivators that drive human (health) behaviour  **K1.7b** Describes the role that values play in steering positive human behaviours  **K1.7c** Describes what a growth mindset is and how it aids in mental health improvement | **S1.7** Demonstrate ability to identify motivators to work on psychological health | **BI1.7a** Express positive attitude that psychological health *training in general* will be beneficial  **BI1.7b** Beliefs that personal identity is congruent with focus of psychological health training  **BI1.7c R**elates personal motivators to importance of engaging in psychological health activities  **BI1.7d** Demonstrate a (positive shift towards) a ’growth’ identity |  | **SOC1.7** Determines the reinforcing or inhibiting impact of their personal social environment on motivation for participation in the program |
| **PO 1.8** | **Develops a personal psychological health strategy** | **K1.8a** Describes the importance of developing a strategy of sufficient intensity  **K1.8b** Explains how to access activities that can be used improve psychological health  **K1.8c** Lists specific strategies that contribute positively to their psychological health | **S1.8a** Practices the use of psychological health activities during training  **S1.8b** Practices the use of psychological health activities after training  **S1.8c** Develops competency in use of psychological health activities in day-to-day life  **S1.8d** Develops the ability to match intervention activities to personal needs | **BI1.8a** Express positive attitude that psychological health *strategy* will be beneficial  **BI1.8b** Express positive attitude to integrating personal psychological health strategy in day-to-day life  **BI1.8c** Beliefs they can identify social supporters within or outside of the program | **GB1.8** Develop personal goal for strategy implementation in day-to-day life | **SOC1.8a** Identifies social support within personal circumstances  **SOC1.8b** Involves social support in development of psychological health strategy |
| **PO 1.8.1** | **Overcomes barriers to implementing a personal psychological health strategy** | **K1.8.1** Explains how barriers can influence successful execution of the psychological health strategy | **S1.8.1a** Identifies personal barriers to enacting psychological health strategy  **S1.8.1b** Develops strategy to overcome barriers to using psychological health activities |  |  |  |
| **PO 1.9** | **Maintains use of personal wellbeing strategy over time** | **K1.9** Demonstrate understanding that improving or maintaining psychological health requires an ongoing commitment | **S1.9a** Performs activities on regular basis according to individual strategy  **S1.9b** Recognises when to use specific activities to improve psychological health | **BI1.9** Express confidence in implementing activities in day-to-day life | **GB1.9** Schedules time to engage in psychological health activities to ensure goal attainment |  |
| **PO 1.10** | **Evaluates implementation of wellbeing strategy** | / | / | / | / | / |
| **PO 1.10.1** | **Judges whether psychological health strategy is being executed successfully** | **K1.10.1a** Understands that different people require different strategies to see effective change in outcomes  **K1.10.1b** describes criteria for successful execution of psychological health strategy | **S1.10.1a** Demonstrates ability to reflect on whether strategy activities are leading to change  **S1.10.1b** Demonstrates ability to determine why strategies are not leading to positive change | **BI1.10.1** Demonstrates self-compassion in the case their strategy is not leading to desired outcomes | **GB1.10.1a** Recall personal psychological health strategy  **GB1.10.1b** Monitors training goal attainment | **SOC1.10.1** Communicates with social relationships whether positive changes can be noted |
| **PO 1.10.2** | **Re-evaluates strategy if not effective at achieving personal outcomes** |  | **S1.10.2a** Practices additional psychological health strategies when other activities do not work out  **S1.10.2b** Demonstrate ability to explain psychological health strategy to social connection | **BI1.10.2a** Demonstrate positive attitude towards effectiveness of re-evaluated psychological health strategy after implementation  **BI1.10.2b** express confidence in discussing wellbeing strategy with social actor | **GB1.10.2a** Recognises ineffective elements of personal wellbeing strategy  **GB1.10.2b** Identifies new activities to be included in strategy | **SOC1.10.2** Schedule time to engage with social actor |
| **PO 1.10.3** | **Contacts professional care when mental health and wellbeing symptoms impact personal life** | **K1.10.3a** Describes personal situation/outcome that warrants professional support  **K1.10.3b** Names contact information for professional support | **S1.10.3a** Illustrates how distress can be monitored to ensure professional support is warranted  **S1.10.3b** Demonstrates capability to reach out to professional support  **S1.10.3c** Compare the objectives of existing program to other services | **BI1.10.3** Demonstrates positive attitude towards the use of professional support | **GB1.10.3** Sets clear goal around what to do when symptoms warrant professional support |  |
| **PO 1.10.4** | **Adjusts strategy and returns to PO 1.8** | **K1.10.4** Understands that adjusting a strategy may lead to better outcomes over the life-course |  | **BI1.10.4** Demonstrate positive attitude towards effectiveness of re-crafted psychological health strategy | **GB1.10.4** Adjust personal goal for strategy implementation in day-to-day life |  |
| *Notes.* Each change objective is coded according to the determinant, i.e. Knowledge (K), skills (S), beliefs about capabilities and consequences, and identity (BI), goals and behavioural regulation (GB) and social influences (SOC), and the performance objective (PO), as depicted by the number after the letter, it belongs to. When multiple change objectives belong to the same determinant and performance objective, change objectives are separated by alphanumeric symbols. | | | | | | |

| Table S2  Matrices of change for behavioural outcome 2 showing the change objectives of each determinant and performance objective (PO). | | | | | | |
| --- | --- | --- | --- | --- | --- | --- |
| **Behavioural outcome 2: Implements a personal resilience plan to prepare for adversity** | | | | | | |
| Code | Performance Objective | Determinants | | | | |
|  |  | Knowledge | Skills | Beliefs in capabilities & consequences | Goals and Behavioural regulation | Social influence |
| **PO 2.1** | **Understands the concept of resilience and its relationship to psychological health** | **K2.1a** Describes the concept of stress and their consequences both positive and negative  **K2.1b** Describes resilience as an outcome  **K2.1c** List positive outcomes associated with improved resilience | **S2.1** Demonstrates ability to process entry-level information on psychological health | **BI2.1** Expresses positive attitude towards learning about resilience and related constructs |  |  |
| **PO 2.2** | **Understands the impact of different stressors types or adversities on psychological health (chronic vs acute, foreseen vs unforeseen)** | **K2.2a** Defines the concept of stressors  **K2.2b** Understands how stressors can be appraised differently and how this impacts stress levels  **K2.2c** Explains how stress can lead to growth | **S2.2** Demonstrates ability to process information on psychological health | **BI2.2a** Accepts that stressors are part of everyday life  **BI2.2b** Accepts that stressors can be mitigated against by developing a strategy to cope |  |  |
| **PO 2.3** | **Understands how effective use of psychological health strategies can lead to improved resilience after difficulty** | **K2.3a** Understands that resilience can be grown through difficulty  **K2.3b** List evidence-based activities that can build resilience  **K2.3c** List positive effects associated with engaging in resilience activities |  | **BI2.3a** Recognise malleability of resilience  **BI2.3b B**eliefs that growth can happen after stress and difficult circumstances |  |  |
| **PO 2.4** | **Understand that their personal characteristics influence which interventions they should be considering to build resilience** | **K2.4** describes role of biology, psychology and social circumstances on resilience | **S2.4** Demonstrates capacity to reflect on personal characteristics | **BI2.4** Expresses positive attitude towards interrogating personal characteristics |  |  |
| **PO 2.5** | **Is aware of personal resilience status** | **K2.5** Explains how to access resilience assessment methods | **S2.5a** Demonstrates ability to complete resilience measures  **S2.5b** Demonstrates ability to interpret scores on psychological health assessment methods | **BI2.5a** Expresses positive attitude towards validity of resilience assessment methods  **BI2.5b** Expresses positive attitude towards measuring their resilience over time |  |  |
| **PO 2.6** | **Identifies potential resources and challenges for their resilience** | **K2.6a** Lists common resources and challenges for resilience  **K2.6b** Explains that resources and barriers for resilience can change over time  **K2.6c** Identifies personal barriers to build resilience  **K2.6d** Identifies personal resources to build resilience | **S2.6** demonstrates capacity to reflect on personal characteristics |  | **GB2.6a** Monitors personal resources and challenges to resilience  **GB2.6b** Reflects on potential future resources and challenges to resilience | **SOC2.6a** Investigates social support for implementation of resilience strategy  **SOC2.6b** Determines influence of social identity to form resilience strategy |
| **PO2.6.1** | **Determines which resources and challenges are currently present, which can be improved on using the program and which ones are out of scope** | **K2.6.1a** Recalls scope of the program  **K2.6.1b** Lists types of resources and challenges that cannot be altered by a psychological skills training  **K2.6.1c** Understands where to find help for psychological issues out of scope of the program | **S2.6.1** Demonstrates ability to identify stressors which can and cannot be managed personally | **BI2.6.1** Demonstrates positive attitude towards reaching out or using other mental health and wellbeing services |  |  |
| **PO 2.7** | **Creates overview of personal motivators for regularly engaging in resilience strategy** |  | **S2.7a** Demonstrate ability to self-reflect on personal situation  **S2.7b** Identifies personal motivators to work on building a resilience strategy | **BI2.7a** Express positive attitude that psychological health *training* will be beneficial to improve resilience  **BI2.7b** Beliefs that building resilience is congruent with identity  **BI2.7c** Relates personal motivators to importance of engaging in resilience activities  **BI2.7d** Demonstrate a (positive shift towards) a ’growth’ identity |  |  |
| **PO 2.8** | **Develops a personal resilience plan** | **K2.8a** List activities that can be used for personal resilience strategy  **K2.8b** Defines what stressor types can and cannot be managed individually | **S2.8a** Practice resilience strategies during training  **S2.8b** Demonstrate practicing of resilience activities after training  **S2.8c** Identifies professional support for out-of-scope symptom levels | **BI2.8a** Express positive attitude that psychological health *strategy* will be beneficial for resilience  **BI2.8b** Express positive attitude to integrating personal psychological health strategy into own identity  **BI2.8c** Express confidence that certain stressors can be managed personally | **GB2.8a** Develop personal goal for strategy implementation in day-to-day life  **GB2.8b** Determines potential barriers to executing goals | **SOC2.8** Involves social support in resilience strategy |
| **PO 2.9** | **Practice resilience strategies on regular basis regardless of presence of stressors** |  |  | **BI2.9** Expresses confidence in implementing resilience practice in day-to-day life | **GB2.9** Schedule time to engage in resilience activities to ensure goal attainment |  |
| **PO 2.10** | **Uses resilience strategies when facing personal stress** | **K2.10a** Describes the mental and physical health symptoms associated with unhealthy reactions to stress  **K2.10b** Demonstrates understanding of individual differences in capacity to deal with stress  **K2.10c** Explains that individual judgement may need to be supplemented with input from social environment | **S2.10** Recognise stress when faced with it | **BI2.10a** Express confidence in effectiveness of strategy in dealing with stress  **BI2.10b** Express confidence in usefulness of reaching out to health professional when needed | **GB2.10** Reflect on personal life to determine presence of stressors or adversity |  |
| **PO 2.11** | **Evaluates implementation of resilience strategy** | / | / | / | / | / |
| **PO 2.11.1** | **Judges whether resilience plan is being executed successfully** | **K2.11.1a** Identifies criteria for successful execution of psychological health strategy  **K2.11.1b** Identifies personal strength and growth in dealing with adversity | **S2.11.1** Demonstrates skills to reflect on whether strategy is executed properly |  | **GB2.11.1a** Recall personal resilience strategy  **GB1.11.1b** Monitors training goal attainment  **GB2.11.1c** Review personal resilience plan at regular intervals | **SOC2.11.1** Communicates with social actor after/leading up to stressor whether strategy seems to have positive effect |
| **PO 2.11.2** | **Re-evaluates resilience strategy if not effective at achieving personal outcomes** | **K2.11.2** Lists barriers to executing resilience strategies | **S2.11.2:** Develops strategy to overcome barriers to using training | **BI2.11.2a** Indicates the confidence to overcome barriers to implementing psychological health strategy  **BI2.11.2b** Demonstrate positive attitude towards effectiveness of re-evaluated plan  **BI2.11.2c:** Express confidence in discussing resilience strategy with social actor | **GB2.11.2a** Recognises ineffective elements of personal wellbeing strategy  **GB2.11.2b** Identifies new activities to be included in strategy  **GB2.11.c** Compares resilience and psychological health scores from before until after adversity | **SOC2.11.2** Schedule time to engage with social actor |
| **PO 2.11.3** | Contacts professional care when mental health and wellbeing symptoms impact personal life | **K2.11.3a** Describes symptoms that warrant professional intervention  **K2.11.3b** Names contact information for professional support | **S2.11.3** Demonstrate ability to reach out to professional support | **BI2.11.3** Demonstrates positive attitude towards the use of professional support |  |  |
| **PO 2.11.4** | Adjusts resilience strategy and returns to PO 1.8 |  |  | **BI2.11.4** Demonstrate positive attitude towards effectiveness of re-evaluated plan | **GB2.11.3:** Adjust personal goals for strategy implementation |  |
| *Notes.* Each change objective is coded according to the determinant, i.e. Knowledge (K), skills (S), beliefs about capabilities and consequences, and identity (BI), goals and behavioural regulation (GB) and social influences (SOC), and the performance objective (PO), as depicted by the number after the letter, it belongs to. When multiple change objectives belong to the same determinant and performance objective, change objectives are separated by alphanumeric symbols. | | | | | | |

| Table S3  Matrices of change for environmental outcome 1 showing the change objectives of each determinant and performance objective (PO). | | | | |
| --- | --- | --- | --- | --- |
| **Interpersonal outcome 1: Relationship supports training participant in striving for more wellbeing and resilience** | | | | |
| Code | Performance Objective | Determinants | | |
|  |  | Knowledge | Beliefs in capabilities & consequences | Goals and Behavioural regulation |
| **PO 3.1** | **Relationship develops understanding of the personal psychological health strategy of the training participant** | **K3.1a** Develops understanding of mental health and the positive effects of engaging in psychological health training  **K3.1b** Describes the components of the training participant’s psychological health strategy  **K3.1c** Describes professional support contact information |  | **GB3.1** Schedules time to learn strategy from training participant |
| **PO 3.2** | **Relationship participates in individual’s psychological health activities when requested** | **K3.2** Describes strategy activities of training participant that their help is wanted for | **BI3.2** Express positive attitude that engaging in psychological activities will be beneficial to individual’s psychological health |  |
| **PO 3.3** | **Relationship checks up if individual is practicing use of strategies over time** |  |  | **GB3.3** Develops personal goal for monitoring strategy use of individual |
| **PO 3.4** | **Relationship reminds individual of thinking about mental health strategies when stress or adversity hits** | **K3.4** Explains how using psychological health strategies can help in dealing with stress |  | **GB3.4** Develops personal goal for monitoring strategy use of individual |
| **PO 3.5** | **Relationship determines whether engaging in training may be beneficial for themselves** | **K3.5** Understands that they can access measurement and training themselves | **BI3.5** Express positive attitude that reflection on personal psychological health profile will be beneficial to themselves |  |
| *Notes.* Each change objective is coded according to the determinant, i.e. Knowledge (K), skills (S), beliefs about capabilities and consequences, and identity (BI), goals and behavioural regulation (GB) and social influences (SOC), and the performance objective (PO), as depicted by the number after the letter, it belongs to. When multiple change objectives belong to the same determinant and performance objective, change objectives are separated by alphanumeric symbols. | | | | |

# Section 4. Be Well Plan program examples (step 4)

This section provides examples of actual Be Well Plan content to illustrate the components highlighted in step 4. It will generally follow the flow of the text that accompanies step 4 in the main manuscript. This section only demonstrates a sample of the activities to ensure readability of this appendix; the workbook for the Be Well Plan alone comprises over 100 pages, mainly the result of visuals and the large activity bank. Access to program materials can be given upon reasonable request. Please contact the main author at [joep.vanagteren@sahmri.com](mailto:joep.vanagteren@sahmri.com).

## Short overview of the five sessions in the Be Well Plan

A description of the Be Well Plan sessions is provided in the main manuscript. In order to ensure readability of the appendix, the sessions are briefly summarised below.

- Session 1: participants explore the reasons for participating in the program, their personal drivers and acquire basic knowledge on mental health and its malleability. This aims to stimulate a mindset for change. They follow by exploring the evidence for different psychological interventions and start creating their first be well plan. They do this by choosing one of many formats of practicing mindfulness, and setting a goal on how to practice it during the week. They get introduced to the formation of habits/implementation intentions as a technique to improve the chance of goal-attainment.
- Session 2: participants reflect on week 1. They get introduced to the concept of self-compassion (as opposed to self-criticism) and how it can be used to learn from failure and shape our thinking patterns. They practice a self-compassion activity. They subsequently use their measurement result stemming from the integrated measurement to pinpoint an outcome they want to work on (wellbeing, resilience, mood, anxiety, stress) and are introduced to activity finders: flow charts that map evidence-based activities to each of the activities. They pick one activity to add to their Be Well Plan and will set new goals for the week. They will be introduced to the use of prompts and reminders as another method to increase goal attainment.
- Session 3: participants reflect on week 2. They will work with (and are reminded of) existing resources to their own mental health via two practical activities. The first one gets participants to choose pictures that display sources of meaning in their life, the second one gets participants to identify core values that can be used to guide goals. They then use a simple questionnaire to identify a key resource or challenge they want to work on. They are introduced to a second activity finder that maps evidence-based activities to each of the challenges and resources. They pick a new activity to add to the Be Well Plan.
- Session 4: participants reflect on week 3. This session focuses on stressful times and effective ways to cope (avoidance-focused coping versus more helpful ways, e.g. problem-focused coping). They are then walked through various ways of coping using psychological techniques and theories, including identification of cognitive traps and the use of thought defusion. They are asked to identify social supporters for when times get too tough and are reminded of various professional services. They then choose one new activity specifically focusing on stress and resilience. They also are actively asked to reach out to a social supporter as part of their weekly activities.
- Session 5: participants reflect on the past 4 weeks. They are asked to complete a new measurement and investigate how their outcomes have changed over the four weeks. The trainer will introduce the concept of realistic optimism, growth and the fact that progress comes with ups and downs. They practice positive reframing as a way to deal with setbacks. They will then build their final Be Well Plan, which aims to summarise key learnings from the previous weeks into a standalone plan. They summarise what their best possible mental health looks like, highlight their unique drivers and motivations, and existing resources and challenges in their life. They set a longer-term goal and choose the activities they wish to add to their Be Well Plan. They identify their key supporters and reflect on what support services they need in case of emergency.

The sessions are designed to be taught over five weeks. Implementation in daily life is a core principle in the program to allow for experimentation with different activities, which means the training is not recommended to be rolled out as an intensive back-to-back course.

## Examples to core components to individual Be Well Plan sessions

The following pages highlight actual example pages for Be Well Plan activities, structured according to the four core principles of self-reflection, sharing between participants, developing core psychological principles, evidence-based psychological activities, planning fundamentals and the integration of the measurement report. It starts with the activity index as it currently stands.

### Index of activities


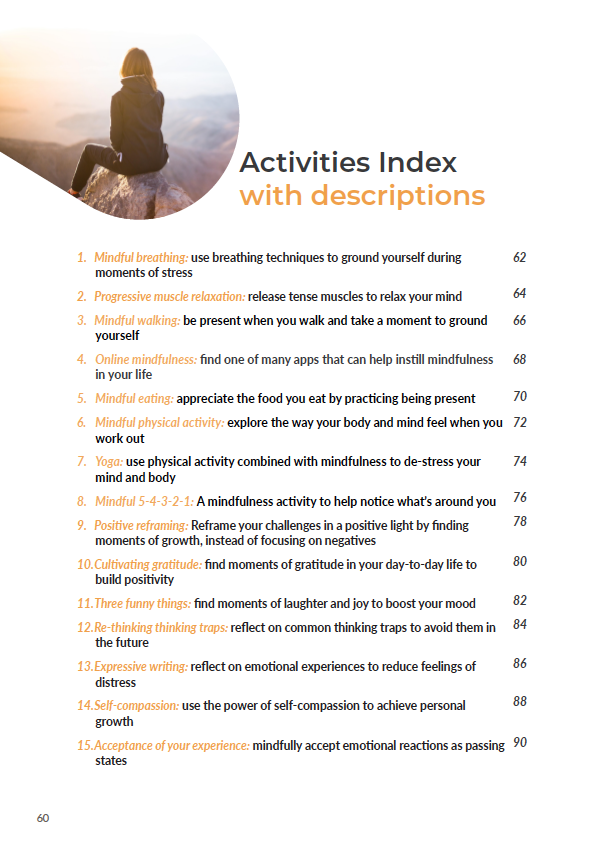


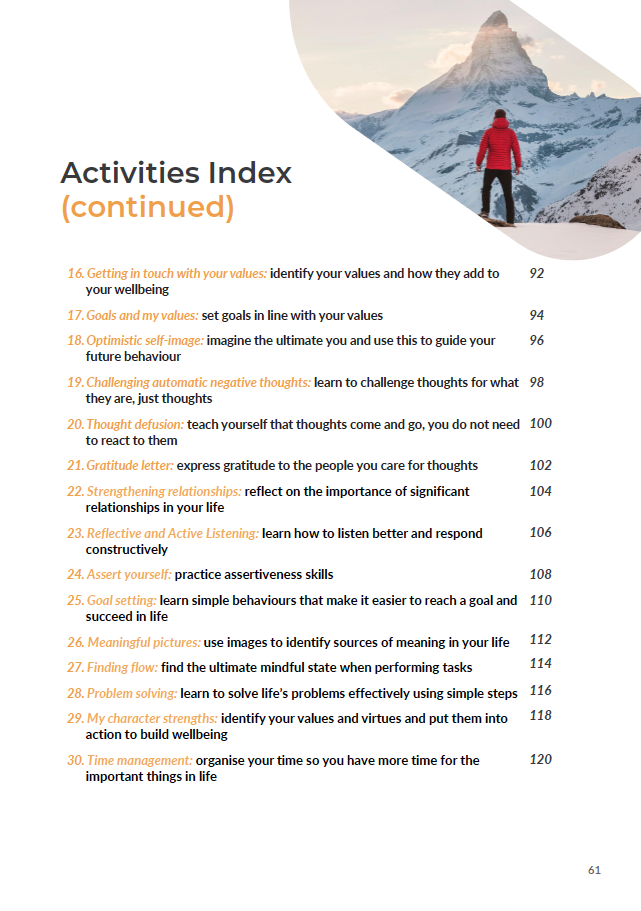


### Examples of self-reflection activities


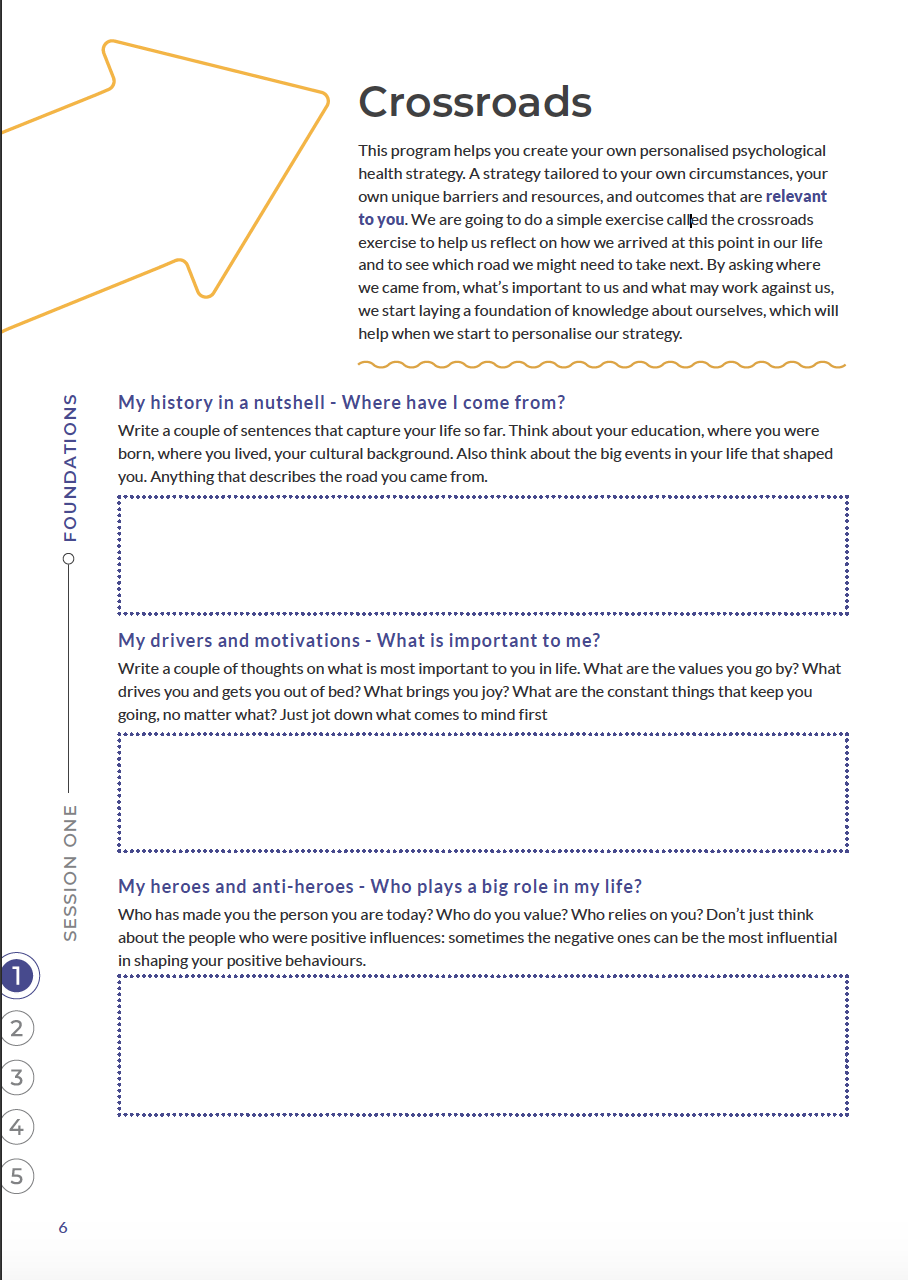


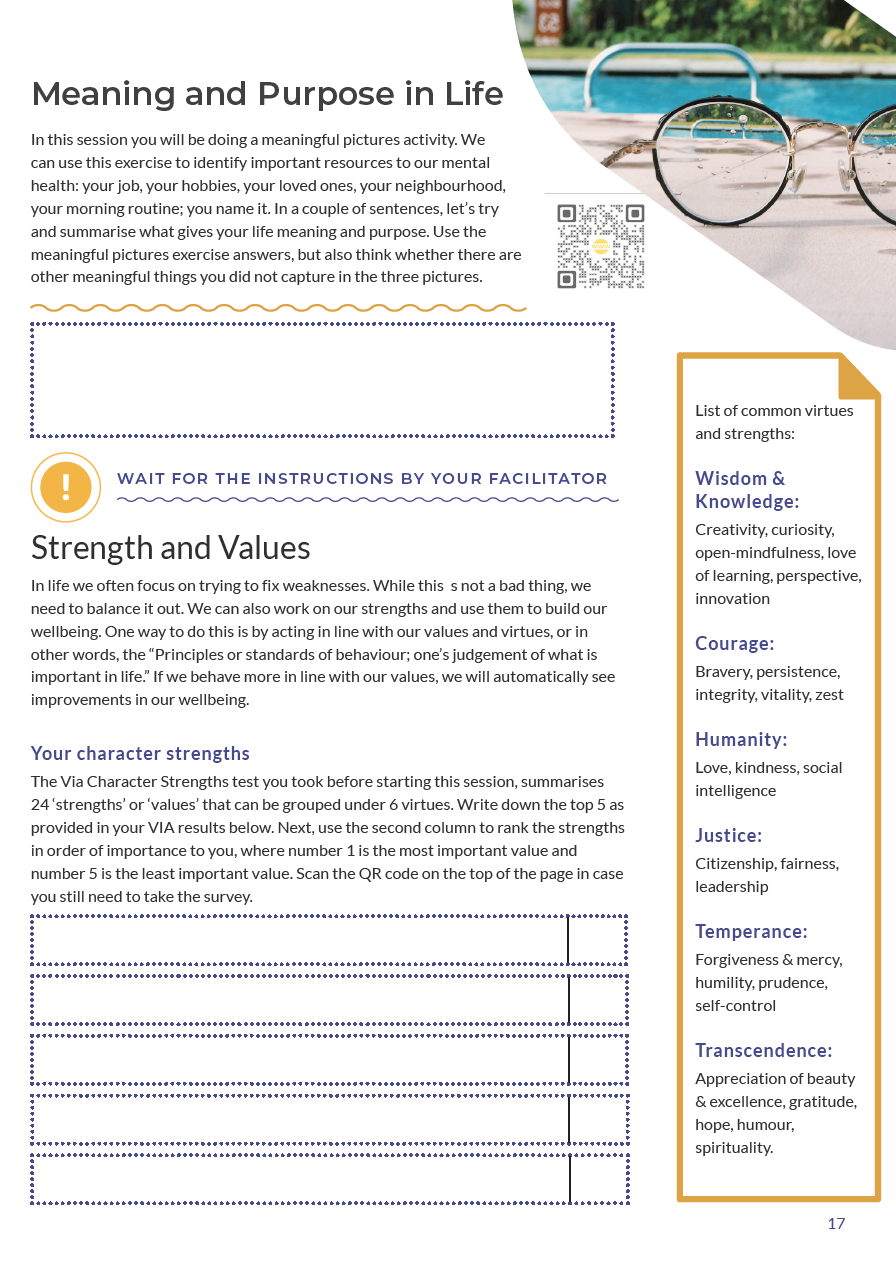


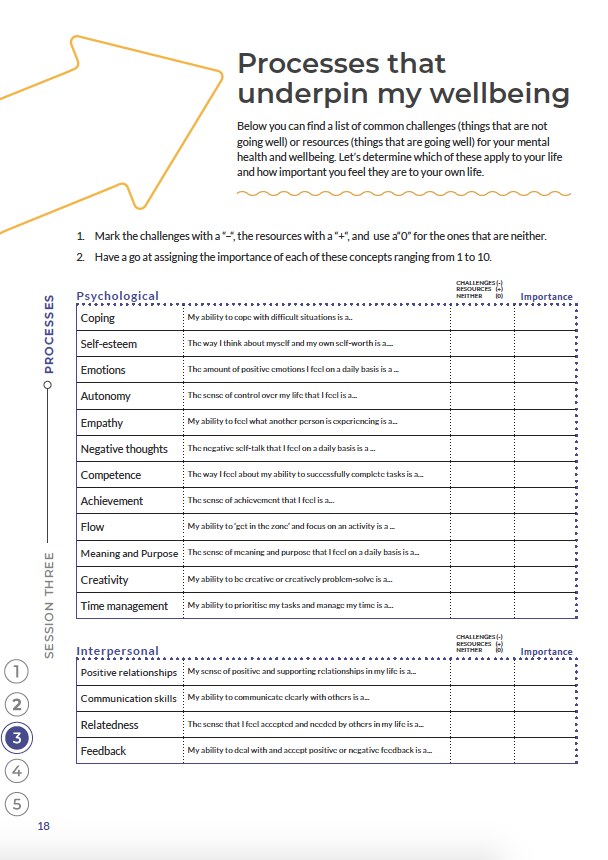


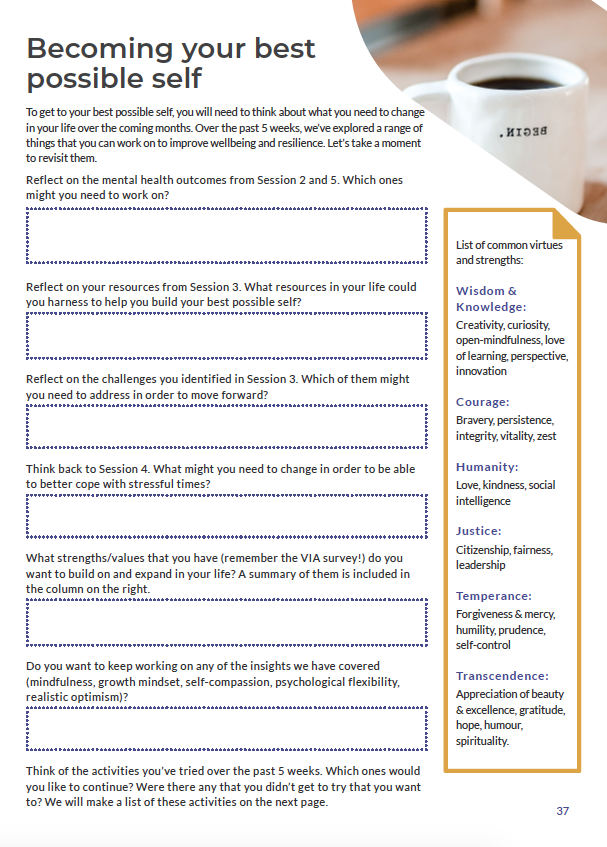


### Examples of sharing exercises between participants


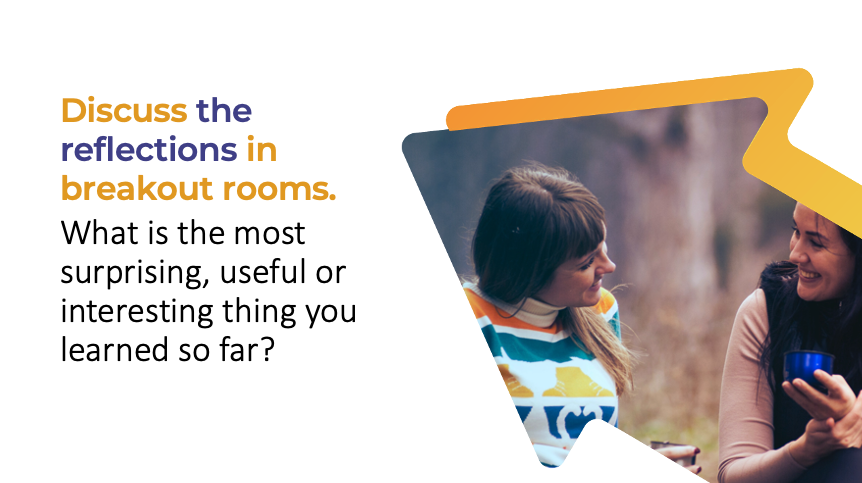


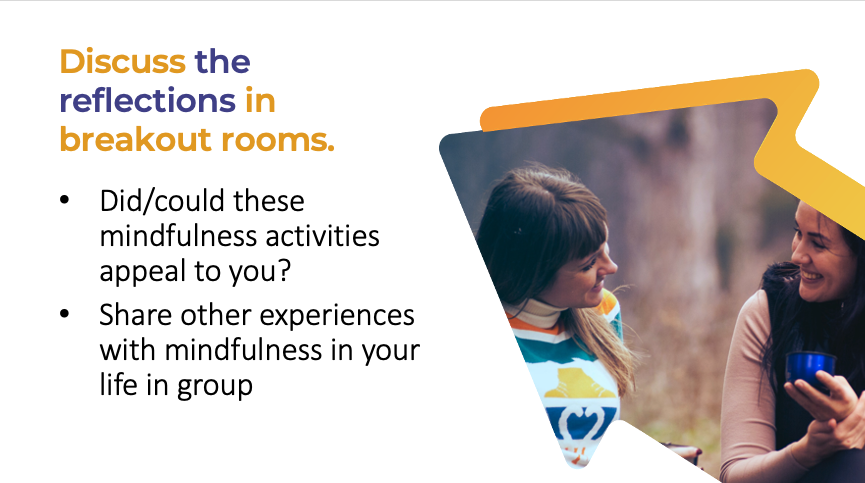


### Examples to highlight core psychological principles participants develop


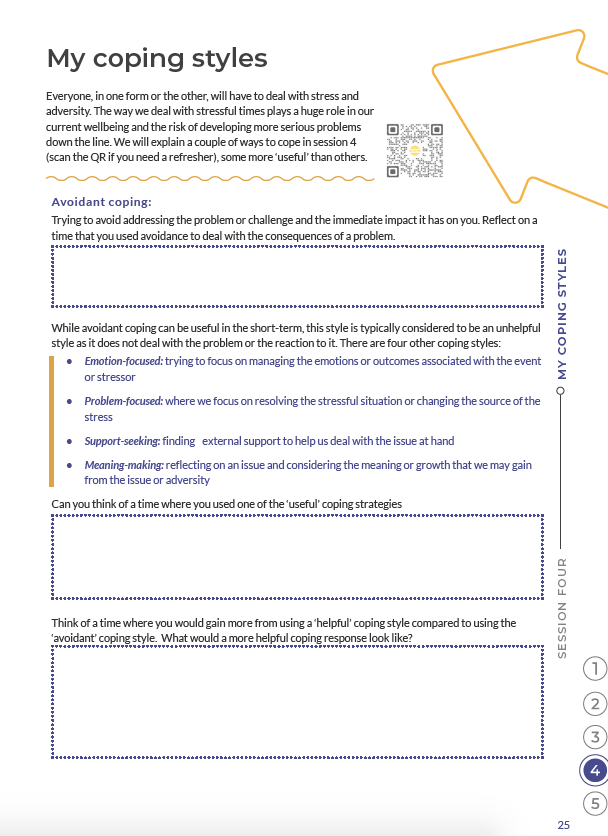


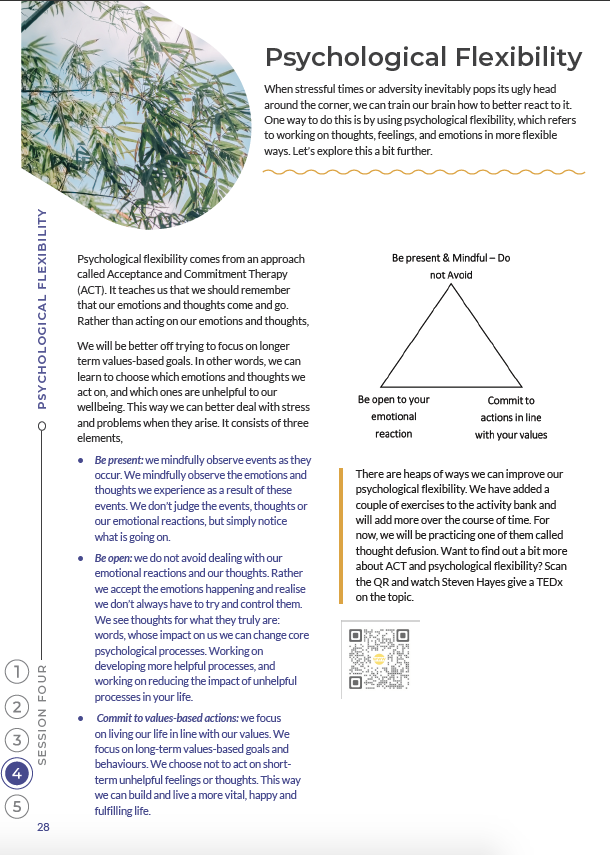


### Examples of the evidence-based psychological activities


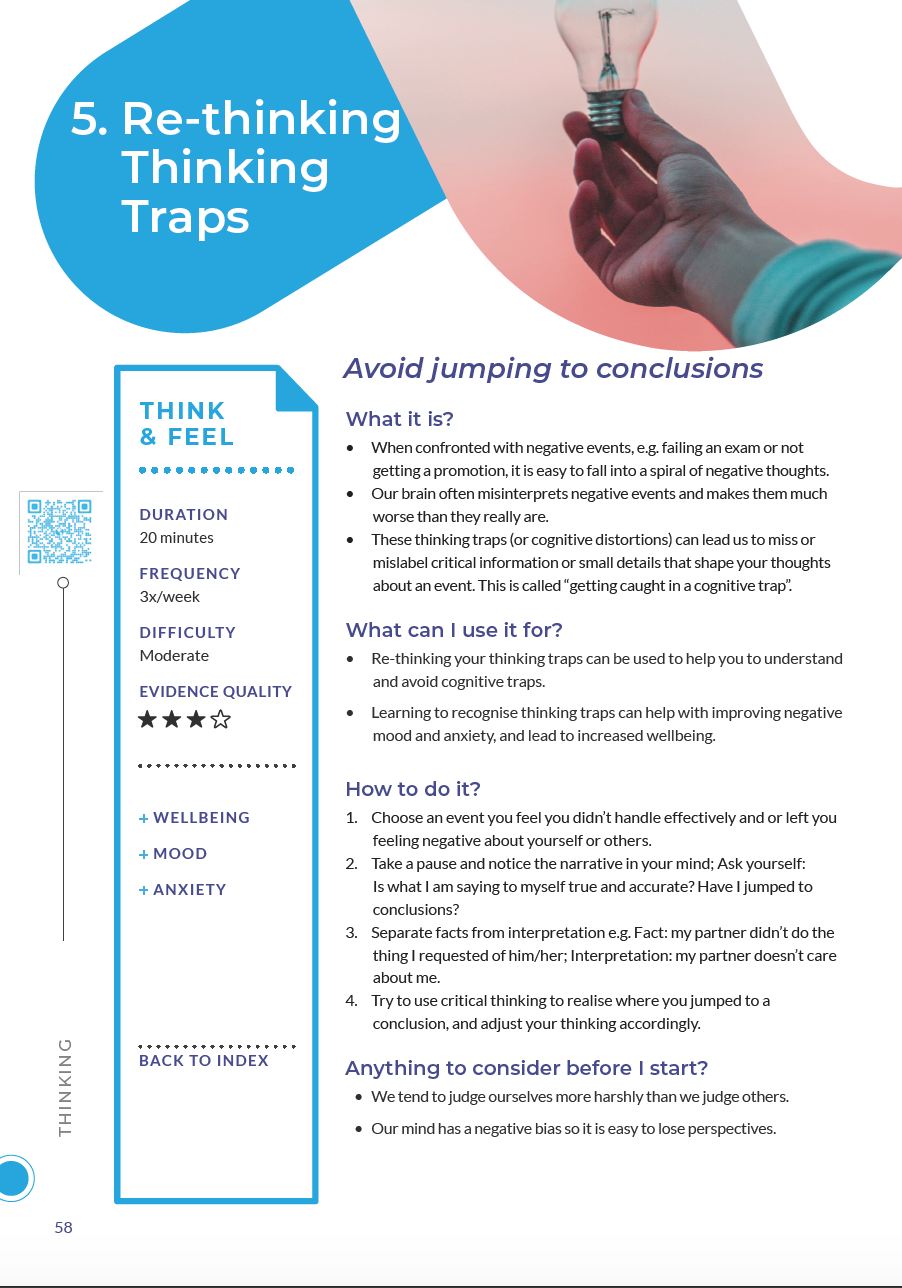


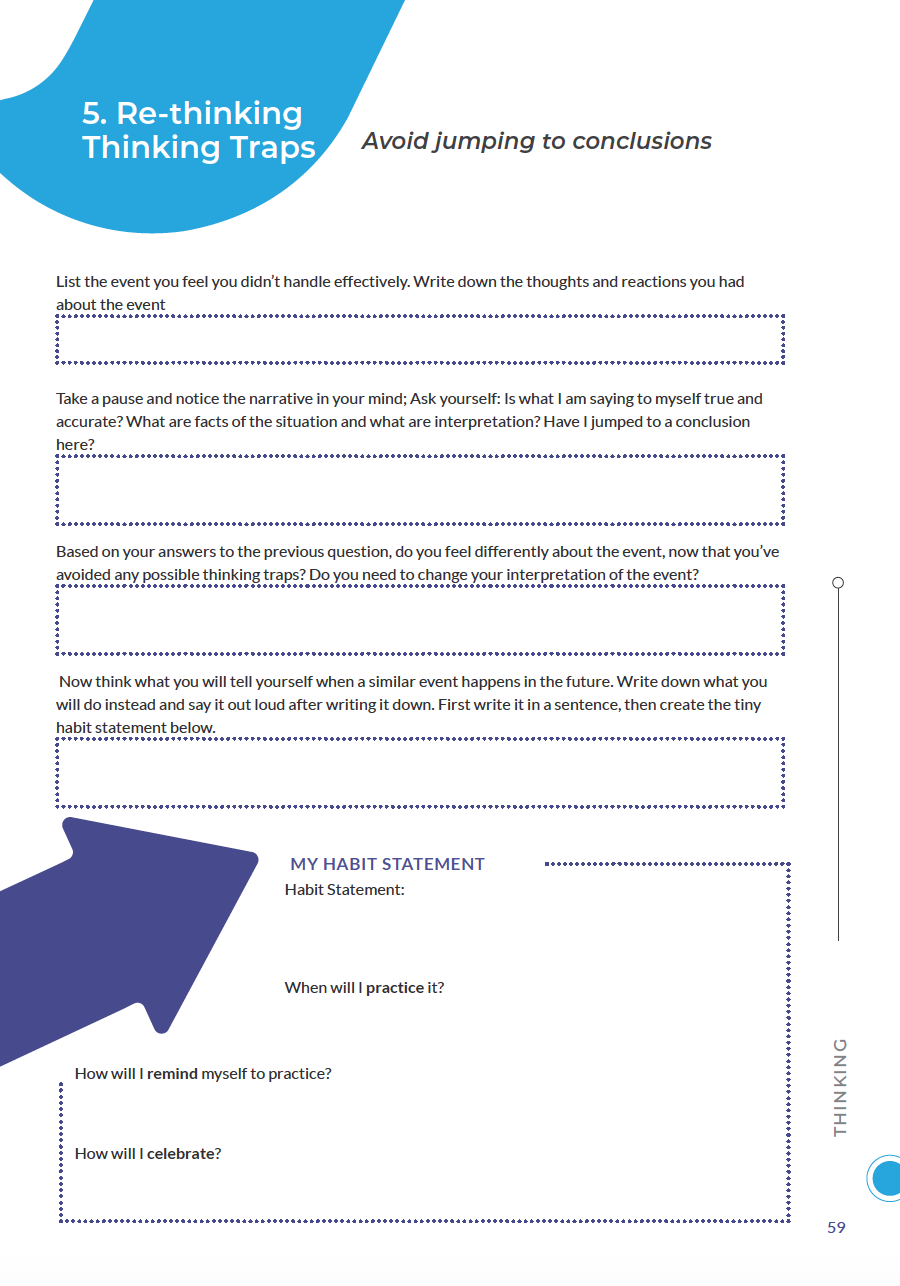


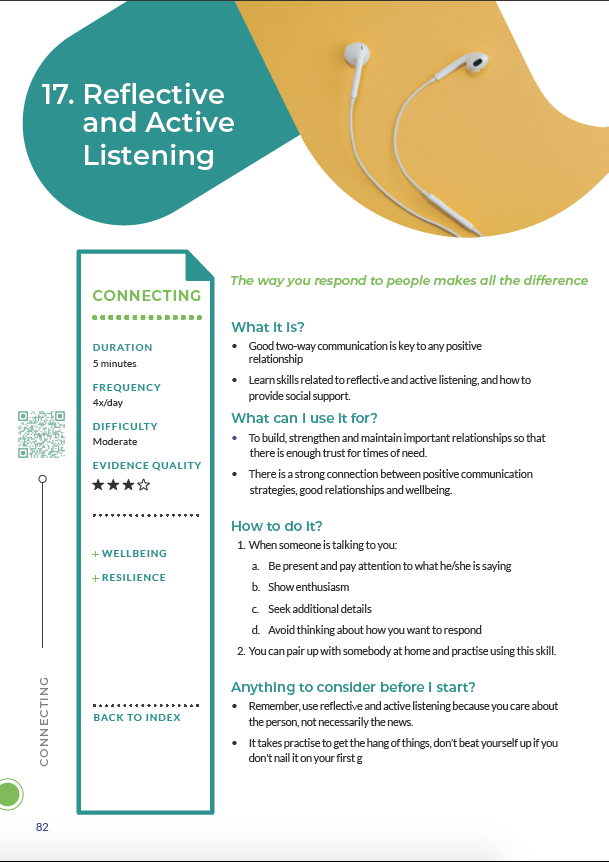


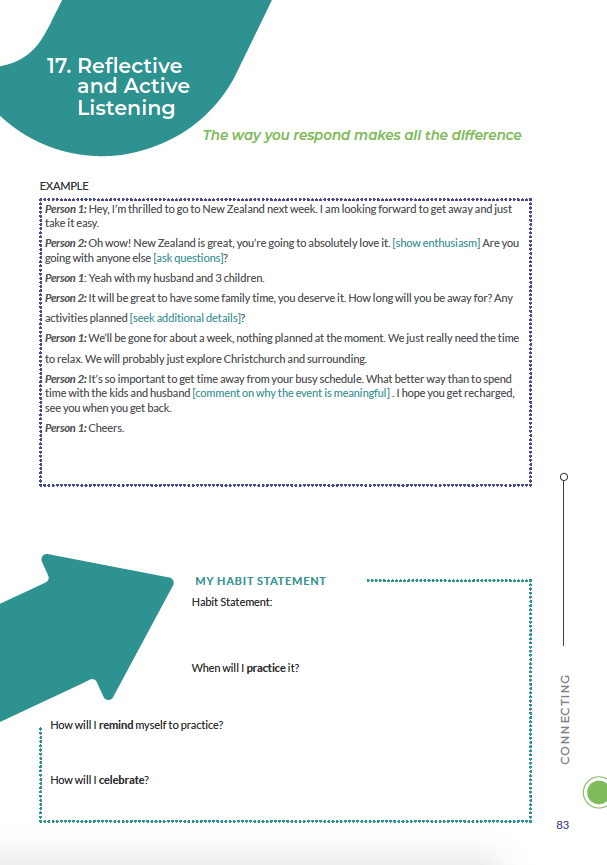


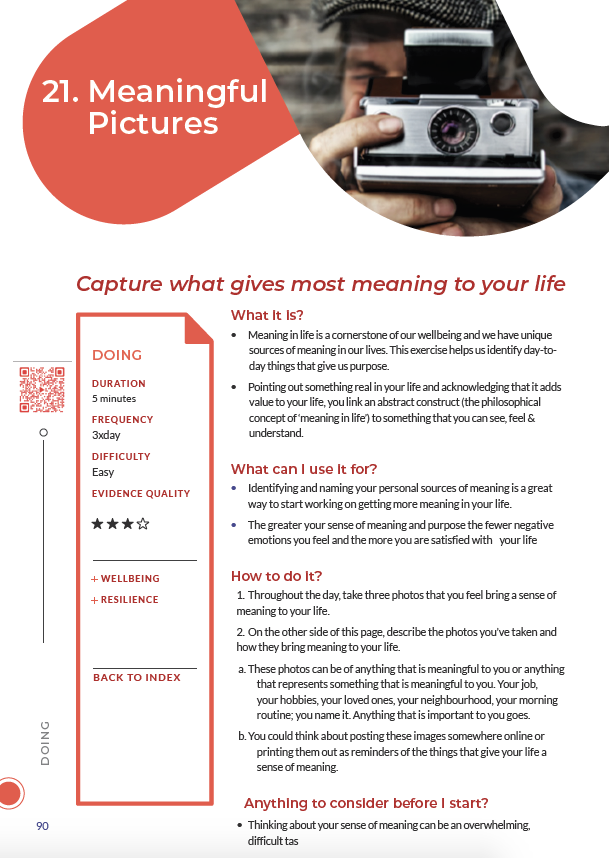


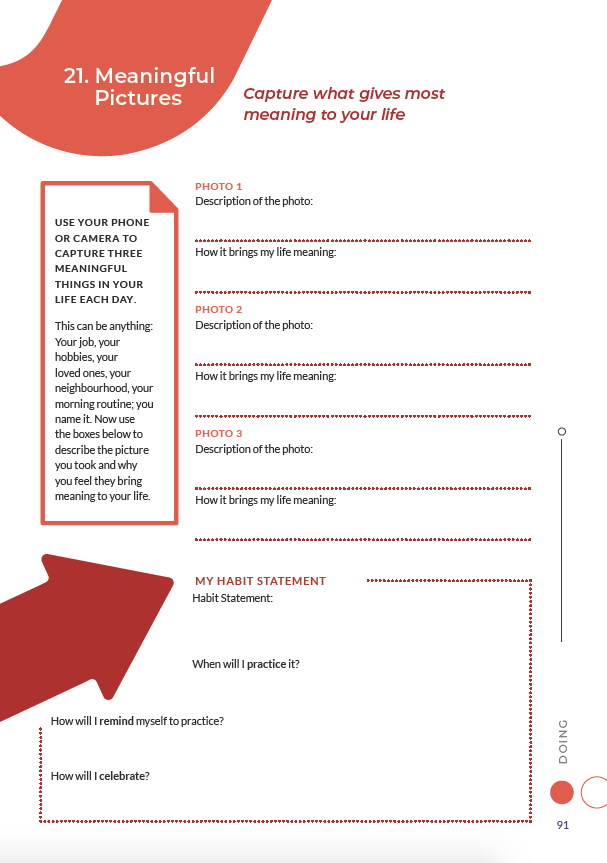


### Planning fundamentals and behaviour change


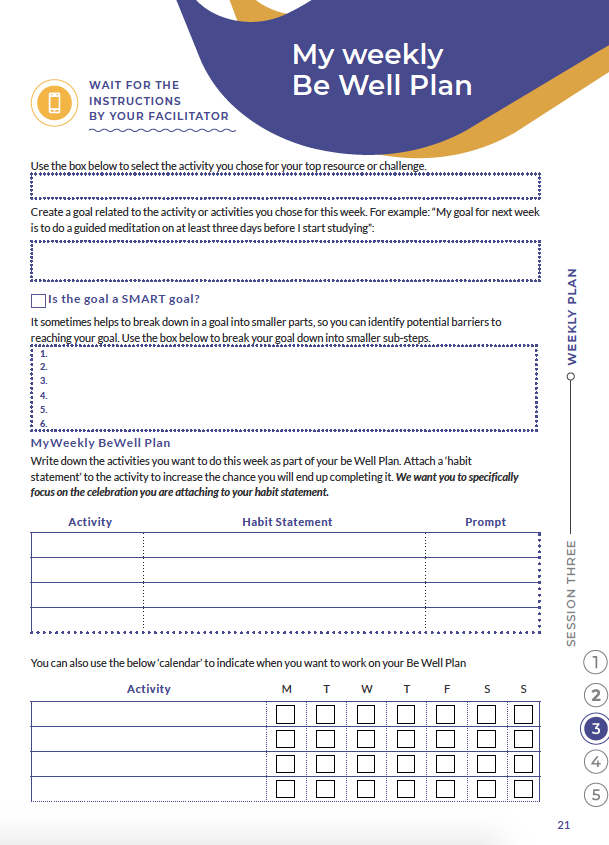


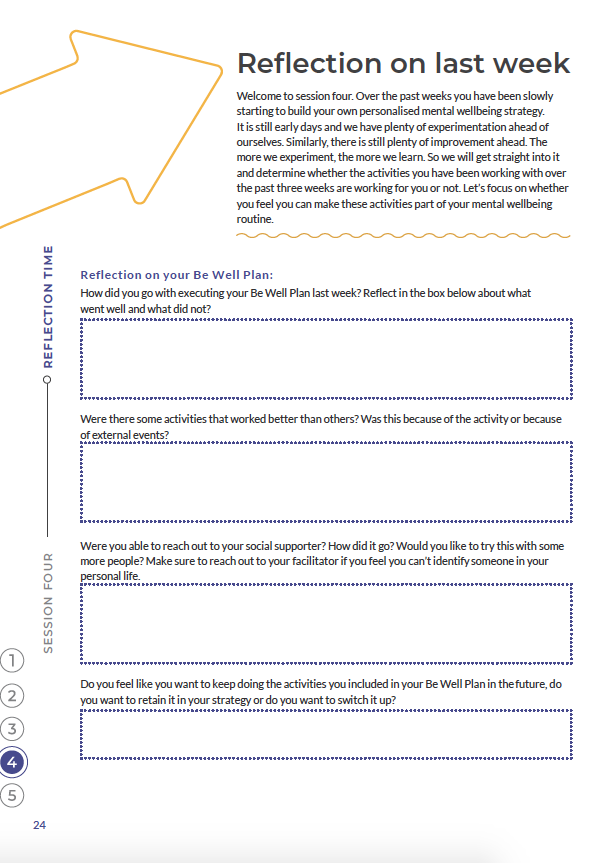


## Example of the measurement report

Participants complete a measurement that provides a summary of their scores on six key domains. The participants interact with the report to improve their strategy over time, via a series of exercises in the workbook.


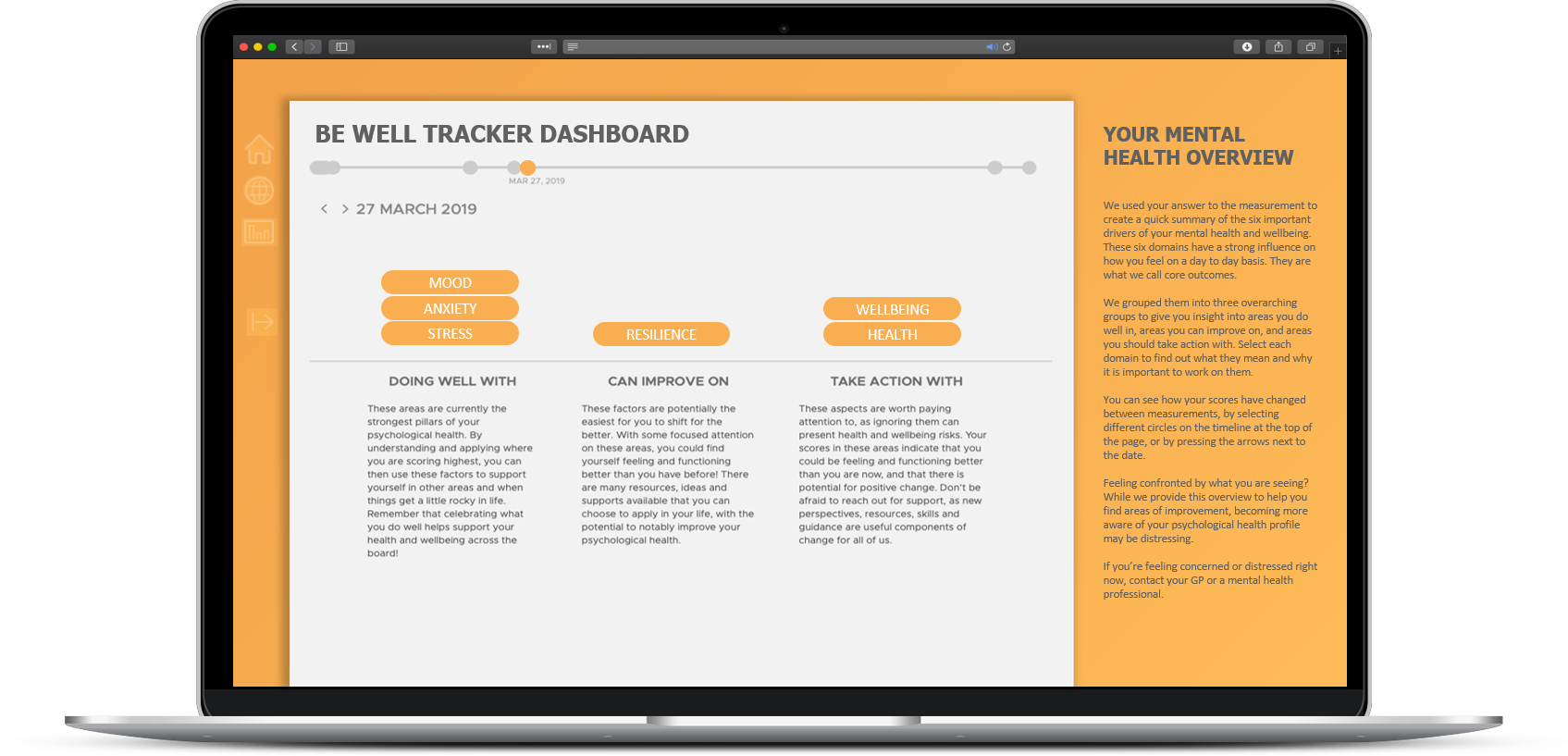


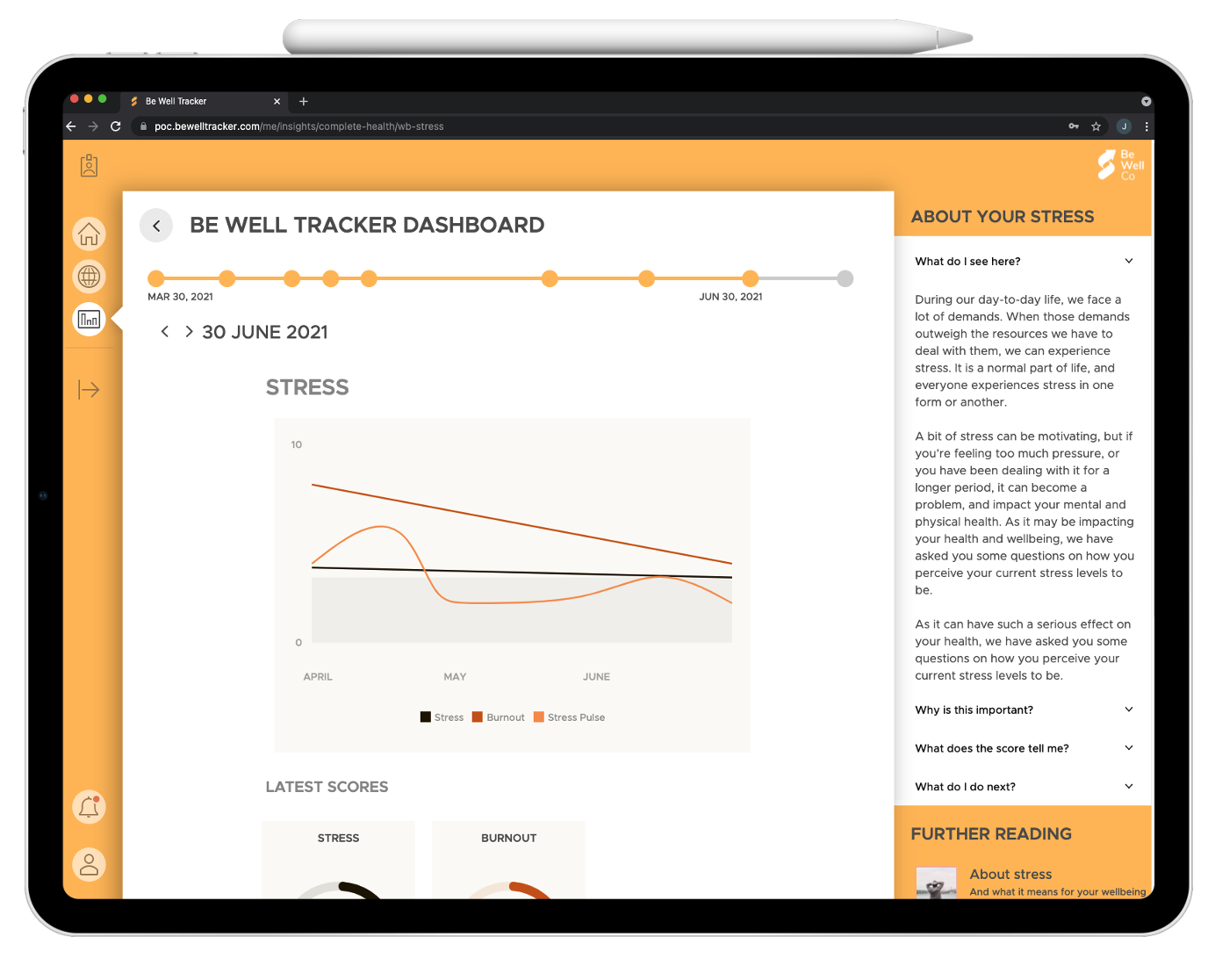


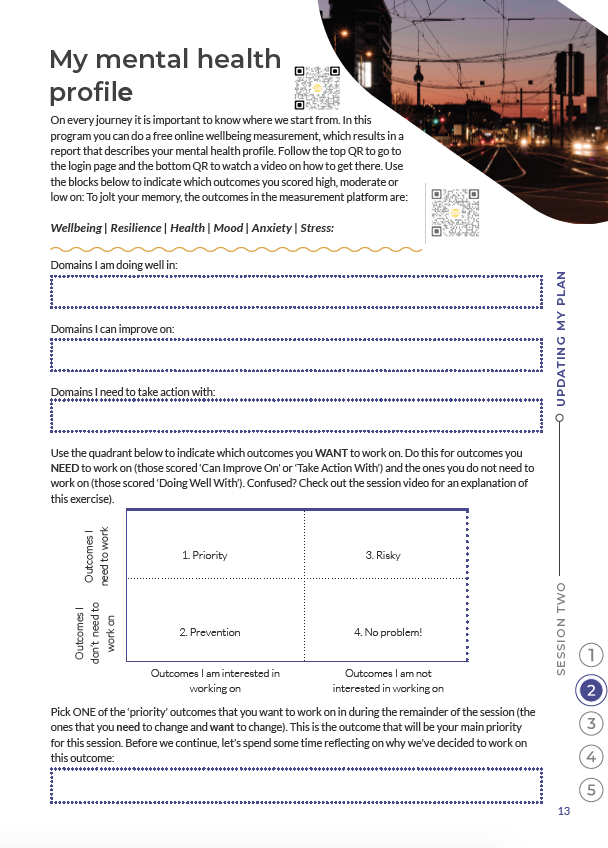


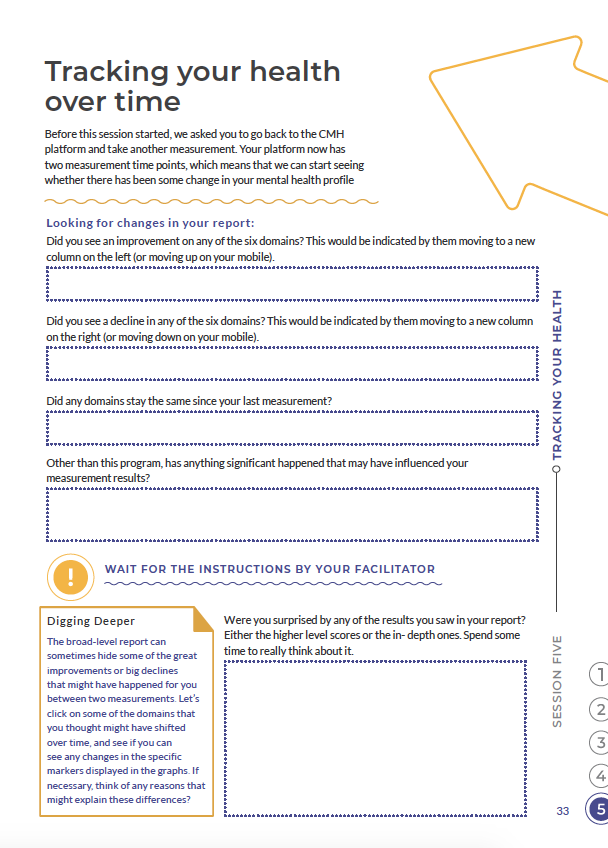


## References for appendix

1. Bartholomew LK, Parcel GS, Kok G, Gottlieb NH, Schaalma HC, Markham CC, . . . Mullen PDC. (2006). Planning health promotion programs: an intervention mapping approach: Jossey-Bass.

2. Zoom version 5.3 [computer software]. (2020). San Jose, California.

3. van Agteren, J., Bartholomaeus, J., Fassnacht, D. B., Iasiello, M., Ali, K., Lo, L., & Kyrios, M. (2020).

Using Internet-Based Psychological Measurement to Capture the Deteriorating Community Mental Health Profile During COVID-19: Observational Study. JMIR Mental Health, 7(6), e20696.

4. Cane J, O’Connor D, & Michie S. (2012). Validation of the theoretical domains framework

for use in behaviour change and implementation research. 7(1), 37.

5. Bartholomaeus JD, Van Agteren J, Iasiello M, Jarden A, & Kelly D. (2019). Positive aging: The impact of a community wellbeing and resilience program. Clinical gerontologist, 42(4), 377-386.

6. Raymond IJ, Iasiello M, Jarden A, & Kelly D. (2018). Resilient futures: An individual and

system-level approach to improve the well-being and resilience of disadvantaged young Australians. Translational Issues in Psychological Science, 4(3), 228.

6. Raymond I, Iasiello M, Kelly D, & Jarden A. (2019). Program logic modelling and complex positive

psychology intervention design and implementation: the ‘Resilient Futures’ case example. International Journal of Applied Positive Psychology, 3(1-3), 43-67.

8. Van Agteren J, Iasiello M, & Lo L. (2018). Improving the wellbeing and resilience of health services

staff via psychological skills training. BMC research notes, 11(1), 924.
